# Systematic Review of Studies That Have Evaluated Screening Tests in Relatives of Patients Affected by Nonsyndromic Thoracic Aortic Disease

**DOI:** 10.1161/JAHA.118.009302

**Published:** 2018-07-31

**Authors:** Giovanni Mariscalco, Radoslaw Debiec, John A. Elefteriades, Nilesh J. Samani, Gavin J. Murphy

**Affiliations:** ^1^ Department of Cardiovascular Sciences University of Leicester and National Institute for Health Research Leicester Cardiovascular Biomedical Research Centre Leicester United Kingdom; ^2^ Aortic Institute at Yale‐New Haven Yale University of Medicine New Haven CT

**Keywords:** aortic disease, genetic testing, mortality, screening, Cardiovascular Disease, Clinical Studies, Aortic Dissection, Aneurysm, Quality and Outcomes

## Abstract

**Background:**

Nonsyndromic thoracic aortic diseases (NS‐TADs) are often silent entities until they present as life‐threatening emergencies. Despite familial inheritance being common, screening is not the current standard of care in NS‐TADs. We sought to determine the incidence of aortic diseases, the predictive accuracy of available screening tests, and the effectiveness of screening programs in relatives of patients affected by NS‐TADs.

**Methods and Results:**

A systematic literature search on PubMed/MEDLINE, Embase, and the Cochrane Library was conducted from inception to the end of December 2017. The search was supplemented with the Online Mendelian Inheritance in Man database. A total of 53 studies were included, and a total of 2696 NS‐TAD relatives were screened. Screening was genetic in 49% of studies, followed by imaging techniques in 11% and a combination of the 2 in 40%. Newly affected individuals were identified in 33%, 24%, and 15% of first‐, second‐, and third‐degree relatives, respectively. Familial NS‐TADs were primarily attributed to single‐gene mutations, expressed in an autosomal dominant pattern with incomplete penetrance. Specific gene mutations were observed in 25% of the screened families. Disease subtype and genetic mutations stratified patients with respect to age of presentation, aneurysmal location, and aortic diameter before dissection. Relatives of patients with sporadic NS‐TADs were also found to be affected. No studies evaluated the predictive accuracy of imaging or genetic screening tests, or the clinical or cost‐effectiveness of an NS‐TAD screening program.

**Conclusions:**

First‐ and second‐degree relatives of patients affected by both familial and sporadic NS‐TADs may benefit from personalized screening programs.

**Clinical Perspective**

**What Is New?**

- Imaging and/or genetic screening is not the current standard of care in relatives of patients affected by nonsyndromic thoracic aortic diseases (NS‐TADs).
- Genetic and/or imaging screening of relatives of patients affected by NS‐TAD can detect more than 30% of patients newly affected by thoracic aortic diseases.

**What Are the Clinical Implications?**

- Routine imaging and genetic testing of relatives of patients affected by nonsyndromic aortopathies should be encouraged.
- The evidence suggests that screening of first‐ and second‐degree relatives of patients affected by familial NS‐TAD and first‐degree relatives of those affected by sporadic NS‐TADs will result in significant numbers of patients with otherwise undiagnosed disease.
- Personalized screening programs determined by the subtype of NS‐TAD and its related genetic mutation have the potential to benefit these patients.

## Introduction

Diseases of the thoracic aorta are increasing in prevalence, accounting for 1% to 2% of all deaths in Western countries.^1^, ^2^, ^3^, ^4^, ^5^ In the United States, diseases of the aorta account for more than 40 000 deaths per year.^1^, ^4^ Thoracic aortic diseases (TADs) are often silent entities with a mortality of almost 80% when presenting as life‐threatening emergencies.^3^, ^6^ Therefore, early diagnosis and treatment are likely to improve long‐term survival. TADs may be syndromic, associated with disorders involving other organs such as Marfan syndrome, or more commonly nonsyndromic, with manifestations restricted to the thoracic aorta.^4^, ^5^ Nonsyndromic TADs (NS‐TADs) may be familial, characterized by the presence of a family history and an autosomal dominant inheritance, or sporadic.^4^, ^5^, ^6^, ^7^ Unlike syndromic TADs, NS‐TADs are not evident from external physical features and abnormalities of other organ systems and are characterized by silent aneurysm formation and dissection.^4^, ^5^ Screening of first‐degree relatives (FDRs) of patients affected by NS‐TAD is therefore recommended for early detection and treatment of asymptomatic disease.^4^, ^5^ However, existing guidelines are based predominantly on the consensus of expert opinion, rather than high‐quality evidence, and the testing modality, frequency, and extent (FDRs versus second‐degree relatives [SDRs]) of screening are not defined.^4^, ^5^ As a consequence, there is widespread variation in the screening of family members of patients with NS‐TADs. To address this area of uncertainty, we performed a systematic review of the evidence for screening in the relatives of patients affected with NS‐TADs with reference to the prevalence of aortic disease, the predictive accuracy of genetic and imaging screening tests, and the effectiveness of screening programs in this high‐risk population.

## Methods

The data, analytic methods, and study materials are available to other researchers for purposes of reproducing the results or replicating the procedure (Supplemental Material).

### Protocol, Registration, and Search Strategy

The search strategy, objectives, study selection and eligibility, data collection, and assessment of study quality are published online and registered in the PROSPERO International Prospective Register of Systematic Reviews (PROSPERO registry—CRD42017064598).^8^ The protocol of the present systematic review is fully reported in Data S1. The review adhered to PRISMA (Preferred Reporting Items for Systematic Reviews and Meta‐Analyses) guidelines (Table S1).^9^

We searched electronic databases (PubMed/MEDLINE, Embase, and Cochrane Library) without date or language restriction from inception to the end of December 2017. A systematic search in the Online Mendelian Inheritance in Man (OMIM) database^10^ on December 31, 2017, was also accomplished. To supplement the electronic search, the “first‐generation” reference lists of pertinent articles were reviewed. Search criteria, adopted keywords, and MeSH terms used in relevant combinations are reported in Data S1.

### Participants

We included studies considering imaging and/or genetic screening tests in probands affected by diseases of the thoracic aorta (aneurysms and/or acute aortic syndrome), and their FDRs, SDRs, and third‐degree relatives (TDRs), with no restriction on ethnicity or age.

### Target Condition

The target condition was disease of the thoracic aorta (aneurysm and/or acute aortic syndrome) defined by the international guidelines on diagnosis and management of patients with TAD.^4^, ^5^ Only NS‐TAD forms were considered in the present review; syndromic TADs or other *forme fruste* of syndromic TAD related to the transforming growth factor β pathway were excluded. Familial NS‐TAD forms were defined as those occurring in families with ≥2 members with a known TAD, but without a clinical diagnosis or history of a syndromic TAD or any other connective tissue disease.^7^ Sporadic TADs were defined as those occurring in patients apparently without a family history of TAD or evidence of syndromic TAD.^4^, ^5^, ^7^

### Index Tests

For the purposes of the review, we included studies that phenotyped participants using the following imaging tests: transthoracic echocardiogram (TTE)/transoesophageal echocardiogram, computed tomography (CT), or magnetic resonance imaging (MRI) of the thoracic aorta, and genetic screening, individually or in combination with the acknowledgement that sensitivities and specificities of CT (100% and 100%, respectively) and MRI (95–100%) are higher when compared with those of transoesophageal echocardiogram and TTE (74–100% and 71–91%, respectively).^11^, ^12^, ^13^, ^14^, ^15^ In some studies, surgery for TAD, postmortem examination, or sudden death were used to assess the aortic phenotype. Molecular genetic testing approaches included a combination of gene‐targeted testing (multigene panel or single gene testing) and whole exome of genome sequencing.^16^, ^17^, ^18^

### Study Selection, Data Collection, and Extraction

Two investigators (G.M. and R.D.) independently reviewed titles, abstracts, and full‐text articles against the specified inclusion criteria for studies regarding screening of relatives of patients with NS‐TADs. Discrepancies were resolved through consensus and consultation with a third investigator (G.J.M.). One reviewer extracted key data from the included studies using a standard dedicated pro forma; a second reviewer checked the collected data for completeness and accuracy. The Tables report full details on study design and quality, setting and population, details, and results of screening. Key study characteristics include details of the patient population (NS‐TAD form, ethnicity, family identification), participants undergoing screening (relatives eligible for screening; family pedigree; total number of screened relatives; numbers of FDRs, SDRs, and TDRs), TAD characteristics (new diagnosis of aortic disease, number/rate of newly diagnosed thoracic aortic aneurysms and/or dissection, rate of unexplained sudden death, age and aortic diameters at dissection, sex preponderance, and aortic disease penetrance), additional concomitant phenotype/clinical features (types and rates), and type of adopted screening modality (imaging and genetic test used, validation processes). The definitions of the extracted variables are fully reported in Data S1.

### Quality Assessment, Data Synthesis, and Analysis

Two investigators (G.M., R.D.) independently appraised all articles that met inclusion criteria. Study quality was assessed using the Newcastle‐Ottawa Scale and the US Preventive Services Task Force.^19^, ^20^ The Cochrane Risk of Bias tool was also used to evaluate the methodological quality of all included studies.^21^

Because of the observational nature of the studies and their clinical heterogeneity, the analyses were largely descriptive, and a narrative and tabular synthesis of all included studies is provided. Inclusion and exclusion criteria for qualitative/quantitative analyses are summarized according to the PICOS (population, intervention, comparator, outcomes, and study design) approach (Table S2). Subgroup analysis considering type of NS‐TAD form, aortic disease (aneurysm and/or dissection), genetic mutation, and screening modality was also conducted. Categorical variables are reported as number and percentage, and continuous variables are reported as mean and SD or median and range, according to distribution. Analyses were performed with SPSS version 24.0 (IBM).

## Results

### Description of Studies and Quality Assessment

Of the 12 897 records identified, 53 studies were included in the systematic review, comprising a total of 2696 screened relatives. The studies were published between 1985 and 2017 (Figure S1).^22^, ^23^, ^24^, ^25^, ^26^, ^27^, ^28^, ^29^, ^30^, ^31^, ^32^, ^33^, ^34^, ^35^, ^36^, ^37^, ^38^, ^39^, ^40^, ^41^, ^42^, ^43^, ^44^, ^45^, ^46^, ^47^, ^48^, ^49^, ^50^, ^51^, ^52^, ^53^, ^54^, ^55^, ^56^, ^57^, ^58^, ^59^, ^60^, ^61^, ^62^, ^63^, ^64^, ^65^, ^66^, ^67^, ^68^, ^69^, ^70^, ^71^, ^72^, ^73^, ^74^ Regions of origin included North America (28 studies), Europe (17 studies), Asia (5 studies), and Australia (3 studies) (Table 1). No randomized trials were identified, and only 1 large cross‐sectional study was conducted including 581 at‐risk relatives.^58^ Study characteristics and collected outcomes are summarized in Tables S3 through S8 and study quality assessment in Table S9.

**Table 1:** Details of Studies Included in the Systematic Review

Study (Author/Y)	Country	NS‐TAD Form	Pedigree (Patients)					Relatives Affected		Penetrance, %	Inheritance (Modality)	Type of Screening	Related Gene
			Total, No.	FDRs, No.	SDRs, No.	TDRs, No.	Probands, No.	No.	%				
Barbier et al 2014^22^	France	FTAAD	40	14	14	0	2	7	18	60	AD	GEN+IMAG	MFAP5
Bee et al 2012^23^	United States	FTAA	54	37	3	0	9	12	22	100	···	GEN	ACTA2, MYH11, TGFBR2
Chamney et al 2015^24^	United Kingdom	FTAAD	14	8	3	0	1	5	36	100	AD	GEN+IMAG	ACTA2
Disabella et al 2011^25^	Italy	FTAAD	37	23	5	4	5	10	27	78	AD	GEN+IMAG	ACTA2
Disertori et al 1991^26^	Italy	FTAAD	30	13	15	0	2	2	7	na	···	IMAG	···
Dong et al 2014^27^	China	FTAAD	64	5	9	30	1	8	13	64	···	GEN+IMAG	TGFBR1
Francke et al 1995^28^	United States	FTAAD	26	15	9	0	1	9	35	67	AD	GEN+IMAG	FBN1
Gago‐Diaz et al 2014^29^	Spain	FTAAD	31	3	10	13	1	6	19	60	AD	GEN	TGFB2
Gago‐Diaz et al 2016^30^	Spain	FTAAD	30	12	14	3	1	10	33	88	AD	GEN	PRKG1
Guo et al 2001^31^	United States^a^	FTAAD	219	n/c	n/c	n/c	n/a	n/c	n/c	n/a	AD	GEN	Locus 5q13‐14^b^
Guo et al 2007^32^	United States^a^	FTAAD	212	n/c	n/c	n/c	n/a	n/c	n/c	48	AD	GEN	ACTA2
Guo et al 2009^33^	United States^a^	FTAAD	269	n/c	n/c	n/c	n/a	n/c	n/c	49	AD	GEN	ACTA2
Guo et al 2011^34^	United States^a^	FTAAD/pAA	28	7	9	6	1	8	29	75	AD	GEN	Locus 12q13‐14^b^
Guo et al 2013^35^	United States^a^	FTAAD	89	40	18	12	6	31	35	100	AD	GEN	PRKG1
Guo et al 2015^36^	United States^a^	BAV/TAA	48	10	14	15	1	7	15	44	AD	GEN	MATA2
Guo et al 2016^37^	United States^a^	FTAAD	65	21	22	13	6	15	23	86	AD	GEN	LOX
Hannuksela et al 2015^38^	Sweden	FTAAD	270	60	89	55	7	37	14	n/a	···	GEN+IMAG	···
Hannuksela et al 2016^39^	Sweden	FTAAD	46	n/c	n/c	n/c	1	n/c	n/c	45	···	GEN+IMAG	MYLK
Harakalova et al 2013^40^	Holland	TAAD/PDA	75	6	15	34	2	13	17	45	AD	GEN	MYH11
Hasham et al 2003^41^	United States^a^	FTAAD	69	4	5	39	1	16	23	75	AD	GEN+IMAG	TGFBR2
Kakko et al 2003^42^	Finland	FTAAD	213	n/c	n/c	n/c	n/a	n/c	n/c	n/a	···	GEN+IMAG	Locus 5q13‐14^b^
Kent et al 2013^43^	United States	BAV/TAA	129	73	21	19	14	34	26	n/a	AD	GEN+IMAG	NOTCH1
Keramati et al 2010^44^	United States	FTAAD	23	10	8	0	1	12	52	90	AD	GEN+IMAG	Locus 15q21 (FBN1?)
Khau Van Kien et al 2004^45^	France	FTAAD/PDA	68	13	21	24	1	7	10	n/a	AD	GEN+IMAG	···
Khau Van Kien et al 2005^46^	France	FTAAD/PDA	87	13	26	38	1	7	8	50	AD	GEN+IMAG	MYH11
Kuang et al 2016^47^	United States^a^	FTAAD	40	n/c	n/c	n/c	n/a	n/c	n/c	75	AD	GEN	FOXE3
Loscalzo et al 2007^48^	United States	BAV/TAA	194	72	37	65	13	44	23	88	AD	GEN+IMAG	···
Marwick et al 1987^49^	Australia	FTADiss	17	7	5	0	1	1	6	n/a	···	IMAG	···
McManus et al 1987^50^	United States	FTADiss	19	7	9	0	1	5	26	n/a	···	IMAG	···
Milewicz et al 1998^51^	United States^a^	FTAAD	123	44	44	7	6	24	20	n/a	AD	GEN+IMAG	···^c^
Morisaki et al 2009^52^	Japan	FTAAD	47	10	6	27	3	11	23	100	···	GEN	ACTA2
Pannu et al 2005^53^	United States^a^	FTAAD	235	18	35	121	4	54	23	79	AD	GEN+IMAG	TGFBR2
Pannu et al 2007^54^	United States^a^	FTAAD	27	16	4	0	2	4	15	45	···	GEN+IMAG	MYH11
Regalado et al 2011^55^	United States^a^	FTAAD/ICA	231	83	64	50	13^d^	43	19	n/a	AD	GEN	ACTA2, TGFBR1, TGFBR2
Regalado et al 2011^56^	United States^a^	FTAAD/ICA/pAA	106	n/c	n/c	n/c	n/a	n/c	n/c	65	AD	GEN	SMAD3
Regalado et al 2011^57^	United States^a^	FTAAD	29	18	6	0	5	10	34	n/a	···	GEN	FBN1
Renard et al 2013^58^	Belgium	FTAAD	97	34	30	7	8	21	22	n/a	AD	GEN	ACTA2, MYH11
Robertson et al 2016^59^	Australia	FTAAD	n/c	n/c	n/c	n/c	270	341	56	n/a	···	IMAG	···
Sherrah et al 2016^60^	Australia	FTAAD	n/c	n/c	n/c	n/c	n/a	n/c	n/c	n/a	···	IMAG	···
Takeda et al 2015^61^	Japan	FTAAD	17	5	6	2	1	4	24	75	···	GEN	MYH11
Teixidó‐Turà et al 2014^62^	Spain	FTAAD	36	8	5	15	1	2	6	10	···	GEN	ACTA2
Tortora et al 2017^63^	Italy	BAV/TAA	97	77	0	0	20	5	7	n/a	···	GEN+IMAG	···^e^
Tran‐Fadulo et al 2006^64^	United States^a^	FTAAD	153	14	45	63	3	18	12	n/a	···	GEN	···
Tran‐Fadulo et al 2009^65^	United States^a^	FTAAD	78	31	23	7	4^f^	26	33	70	AD	GEN	TGFBR1
Vaughan et al 2001^66^	United States^a^	FTAA	67	27	20	2	3	27	40	n/a	AD	GEN+IMAG	Locus 11q23.3‐24^b^
Wang et al 2010^67^	United States^a^	FTADiss	48	n/c	n/c	n/c	n/a	n/c	n/c	50	AD	GEN	MYLK
Wang et al 2013^68^	China	FTAAD	10	7	0	0	1	1	10	n/a	···	GEN	···
Ware et al 2014^69^	United States	FTAAD	7	4	0	0	2	0	0	100	···	GEN	ACTA2
Warnes et al 1985^70^	United States	FTAAD	6	4	0	0	2	0	0	n/a	···	IMAG	···
Weigang et al 2007^71^	Germany	FTAAD	26	n/c	n/c	n/c	n/a	n/c	n/c	n/a	AD	GEN+IMAG	···
Yoo et al 2010^72^	Korea	FTAAD	20	7	7	0	1	4	20	67	AD	GEN	ACTA2
Zhu et al 2006^73^	France	FTAAD/PDA	49	n/c	n/c	n/c	n/a	n/c	n/c	44	AD	GEN+IMAG	MYH11
Ziganshin et al 2015^74^, ^g^	United States	FTAAD	27	7	11	2	1	3	11	70	AD	GEN	MYLK
Ziganshin et al 2015^74^, ^g^	United States	FTAAD	17	6	8	0	1	6	35	70	···	GEN	TGFBR1

AD indicates autosomal dominant; BAV, bicuspid aortic valve; FDRs, first‐degree relatives; FTAA, familial thoracic aortic aneurysm; FTADiss, familial aortic dissection; FTAAD, familial thoracic aortic aneurysm and dissection; GEN, genetic; ICA, intracranial aneurysm; IMAG, imaging; n/a, not available; NS‐TAD, nonsyndromic thoracic aortic disease; n/c, not computable; pAA, peripheral artery aneurysm; PDA, patent ductus arteriosus; SDRs, second‐degree relatives; TAA, thoracic aortic aneurysm; TAAD, thoracic aortic aneurysm and/or dissection; TDRs, third‐degree relatives.

a Study performed at University of Texas.

b Mapped loci without identified gene.

c No linkage to FBN1 or TAAD2.

d Four probands not affected by aortic diseases (aortic aneurysm and/or dissections).

e No linkage with ACTA2.

f One proband not affected by aortic diseases (aortic aneurysm and/or dissection).

g Data of 2 different screened families obtained from the same study.

### Target Condition

Four main groups of familial NS‐TADs were identified: (1) those characterized by the presence of both aneurysms and dissections in the family pedigree (familial thoracic aortic aneurysm and dissection; 44 studies); (2) those characterized by aneurysmal disease only (familial thoracic aortic aneurysm; 2 studies); (3) those characterized by aortic dissection only (familial thoracic aortic dissection; 3 studies); and (4) thoracic aortic aneurysm forms associated with the presence of bicuspid aortic valve (4 studies). Among the familial thoracic aortic aneurysm and dissection forms, 3 additional subgroups were discovered based on the concomitant presence of patent ductus arteriosus (n=4), intracranial aneurysms (n=2), or peripheral arterial aneurysms (n=2) (Table 1).

### Index Tests

Screening for TAD was performed using 2‐dimensional TTE in 27 (51%) studies, of which 15 (28%) employed 2‐dimensional TTE alone and the remaining 8 (15%) used 2‐dimensional TTE in association with CT and/or MRI. In 5 (9%) studies only, imaging screening included the simultaneous employment of 2‐dimensional TTE, CT, and MRI. In a further 26 (49%) studies, aortic phenotype (presence of an aortic aneurysm and/or dissection) was defined by reported clinical events including acute aortic syndrome, diagnosis made during routine diagnostic clinical care, or postmortem examination. The aortic diameter cutoff used for defining a critical dilation of the aorta varied among studies as the aortic site where the measurements were made (Table S7).

No study reported the sensitivity, specificity, or other measures of diagnostic accuracy for the index tests. One study reported 10‐year longitudinal follow‐up for relatives of patients with NS‐TAD.^59^ In this study, relatives with evidence of aortic dilatation were offered annual follow‐up imaging with prescription of β‐blockers or angiotensin receptor blockers at maximal tolerated doses. Relatives with no evidence of aortic dilatation (unaffected) were subjected to clinical review every 3 years. In the affected relatives (n=114) with serial aortic measurements over 4.5±4.4 years, a mean rate of increase in the aortic diameter of 0.56±0.76 mm per year was observed. No difference in the rate of aortic dilatation was observed between males and females or in patients receiving β‐blockers or angiotensin receptor blockers. No correlation with the age at diagnosis, the initial aortic diameter, and the systolic or diastolic blood pressure was documented. During 10‐year follow‐up, 9% of newly diagnosed relatives were affected by an aortic dissection, and 18% underwent elective aortic surgery. Six relatives (of 368) originally diagnosed as unaffected (initial aortic diameter with a *Z* score <2) experienced a subsequent aortic dissection.^59^

### Results of Imaging Tests

**Figure 1:**
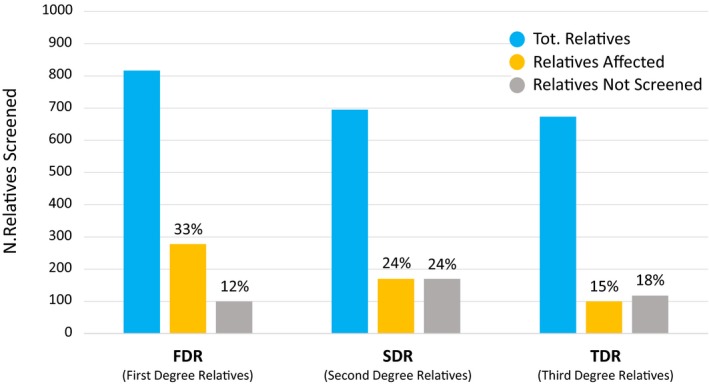
Relatives screened in the studies included in the systematic review. Details for newly affected and not screened individuals are provided for first‐, second‐, and third‐degree relatives (FDRs, SDRs, and TDRs, respectively).

A total of 1039 families underwent screening for NS‐TAD, with a median number of patients in each family pedigree of 48 (study range: 6–270) (Table S3). The proportion of potential eligible patients per family was 73% (study range: 50–100%), while the rate of relatives effectively screened was 54% (study range: 5–100%) (Table 2 and Table S4). FDRs, SDRs, and TDRs were variably screened throughout the studies. Twelve percent of FDRs, 24% of SDRs, and 18% of TDRs were not available for screening (Figure 1).

**Table 2:** Details of Newly Diagnosed Diseases of the Thoracic Aorta in the Screened Relatives

Study (Author/Y)	No. of Relatives Screened	Patients Affected^a^ (Aortic Aneurysm+Aortic Dissection)				Sudden Death (Unexplained)		Aortic Aneurysm^a^		Aortic Dissection^a^			
		No.	%	Male	%	No.	%	No.	%	No.	%	Age at Dissection, y	Range (Age, y)
Barbier et al 2014^22^	13	9	23	3	33	n/a	···	8	89	1	11	58	n/a
Bee et al 2012^23^	32	21	39	16	76	n/a	···	21	100	0	0	···	···
Chamney et al 2015^24^	6	6	43	4	67	0	0	3	50	3	50	49±10.4	37–55
Disabella et al 2011^25^	29	15	41	8	53	1	3	6	40	9	60	49.3±16.3	29–73
Disertori et al 1991^26^	14	4	13	4	100	n/a	···	2	50	2	50	46±2.8	44–48
Dong et al 2014^27^	39	9	14	7	78	1	2	6	67	3	33	39±6.9	35–47
Francke et al 1995^28^	23	10	38	6	60	n/a	···	8	80	2	20	55±14.1	45–65
Gago‐Diaz et al 2014^29^	12	7	23	5	71	n/a	···	5	71	2	29	37.5±4.9	34–41
Gago‐Diaz et al 2016^30^	14	11	37	6	55	1	3	5	45	6	55	34.2±12.9	15–48
Guo et al 2001^31^	121	73	33	47	64	n/a	···	n/a	···	n/a	···	···	···
Guo et al 2007^32^	130	53	25	33	62	n/a	···	8	15	45	85	37.3±13.9	13–67
Guo et al 2009^33^	163	66	25	39	59	n/a	···	n/a	···	n/a	···	···	···
Guo et al 2011^34^	18	9	32	9	100	n/a	···	8	89	1	11	32	n/a
Guo et al 2013^35^	39	37	42	16	43	n/a	···	15	41	22	59	31.1±10.3	17–51
Guo et al 2015^36^	34	8	17	5	63	1	2	8	100	0	0	···	···
Guo et al 2016^37^	21	21	32	17	81	2	3	17	81	4	19	44.8±15.1	25–60
Hannuksela et al 2015^38^	106	44	17	32	73	0	0	27	61	17	39	48^b^	15–75
Hannuksela et al 2016^39^	19	6	13	4	67	0	0	0	0	6	100	53.2±21.1	23–75
Harakalova et al 2013^40^	40	15	20	10	67	3	4	4	37	11	73	46.6±19.5	18–70
Hasham et al 2003^41^	52	17	25	14	82	n/a	···	9	53	8	47	45.4±21.5	14–72
Kakko et al 2003^42^	115	39	18	25	64	n/a	···	26	67	13	33	53.2±15.5	26–80
Kent et al 2013^43^	93	48	37	37	77	n/a	···	n/a	···	n/a	···	···	···
Keramati et al 2010^44^	15	13	57	6	46	n/a	···	10	77	3	23	n/a	n/a
Khau Van Kien et al 2004^45^	49	8	12	6	75	3	4	4	50	4	50	n/a	n/a
Khau Van Kien et al 2005^46^	78	8	9	6	75	2	2	4	50	4	50	n/a	n/a
Kuang et al 2016^47^	16	11	28	11	100	n/a	···	0	0	11	100	44.3±22.6	9–88
Loscalzo et al 2007^48^	138	57	29	42	74	n/a	···	n/a	···	n/a	···	···	···
Marwick et al 1987^49^	4	2	12	1	50	0	0	0	0	2	100	26.5±3.5	24–29
McManus et al 1987^50^	8	6	32	5	83	n/a	···	0	0	6	100	33.5±14.9	22–62
Milewicz et al 1998^51^	n/a	30	24	18	60	9	7	12	40	18	60	42.9±11.3	22–62
Morisaki et al 2009^52^	9	14	30	10	71	5	11	3	21	11	79	36.8±10.1	25–52
Pannu et al 2005^53^	72	58	25	39	66	n/a	···	27	46	32	54	46.1±16.3	14–73
Pannu et al 2007^54^	23	6	22	4	67	n/a	···	1	17	5	83	45±8.8	37–56
Regalado et al 2011^55^	12	52	23	35	67	7	3	9^c^	17	43^b^	83	50.8±13.7	25–76
Regalado et al 2011^56^	36	23	22	14	61	1	1	9	39	14	61	42^d^	25–54
Regalado et al 2011^57^	11	15	52	8	53	n/a	···	7	47	8	53	32.3±9.9	18–50
Renard et al 2013^58^	29	29	30	16	55	3	3	14	48	15	52	48.0±21.2	33–63
Robertson et al 2016^59^	581	486	38	266	72	n/a	···	370	76	116	24	50±13	n/a
Sherrah et al 2016^60^	119^e^	n/a	n/a	68	76	n/a	···	n/a	···	n/a	···	n/a	···
Takeda et al 2015^61^	9	5	29	4	80	0	0	1	20	4	80	47.8±16.6	32–70
Teixidó‐Turà et al 2014^62^	10	3	8	2	67	1	3	1	33	2	67	46.5±12	38–55
Tortora et al 2017^63^	77	25	26	61	79	n/a	···	25	100	0	0	···	···
Tran‐Fadulo et al 2006^64^	9	21	14	7	33	0	0	4	19	17	81	32.0±12.3	16–55
Tran‐Fadulo et al 2009^65^	49	29	37	17	59	0	0	15	52	14	48	n/a	14–62
Vaughan et al 2001^66^	63	30	45	8	27	n/a	···	n/a	···	n/a	···	···	···
Wang et al 2010^67^	21	10	21	5	50	2	4	0	0	10	100	54.3±20.8	16–78
Wang et al 2013^68^	8	2	20	2	100	0	0	1	50	1	50	n/a	n/a
Ware et al 2014^69^	7	2	20	2	100	0	0	0	0	2	100	17	···
Warnes et al 1985^70^	2	2	33	2	100	0	0	0	0	2	100	35.0±18.4	22–48
Weigang et al 2007^71^	23	9	35	5	56	0	0	3	33	6	67	32^c^	18–47
Yoo et al 2010^72^	6	5	25	1	20	0	0	0	0	5	100	32.5±12.9	20–46
Zhu et al 2006^73^	49	8	16	7	88	n/a	···	5	63	3	38	n/a	n/a
Ziganshin et al 2015^74^, ^f^	15	4	15	2	50	n/a	···	1	25	3	75	n/a	n/a
Ziganshin et al 2015^74^, ^f^	15	7	41	4	57	n/a	···	4	57	3	43	n/a	n/a

n/a indicates not available.

a Percentage calculated in the family pedigree (as per protocol).

b Median available only.

c Data available from 4 families only (TAA288, TAA062, TAA549, TAA395).

d Mean available only.

e Comprehensive of patients affected by bicuspid aortic valve.

f Data of 2 different screened families obtained from the same study.

A total of 893 FDRs, 695 SDRs, and 670 TDRs were identified in the family pedigrees of the included studies (Table S5). Of these, a total of 910 newly affected relatives were detected, with an average among studies of 22 newly diagnosed individuals. The percentage of newly diagnosed relatives was 23% (study range: 6–56%). Newly diagnosed individuals were male in 67% of the cases (study range: 20–100%). Sudden unexplained deaths were reported in 2% of the cases (study range: 0–9%). Detailed data about rates of newly affected and screened FDRs, SDRs, and TDRs are depicted in Figure 1.

The type of aortic diseases (aneurysm and dissection rates), male preponderance rate, and age at dissection are summarized in Table 2 and Table S4. Only 1 study screened the relatives of 53 probands identified as affected by a sporadic NS‐TAD form, identifying 83 of 321 newly affected relatives.^59^

### Results of Genetic Tests

**Figure 2:**
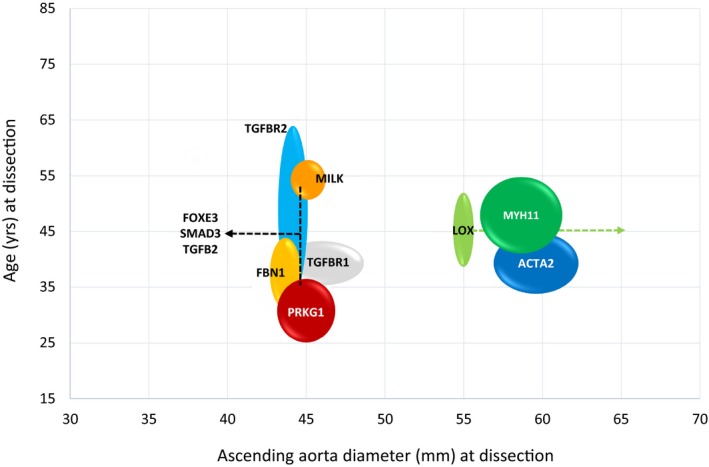
Schematic representation of genetic mutations with age and ascending aorta diameter at dissection. The widening of the circles/lines represents SD in terms of age and diameters. Data are obtained from studies included in the systematic review. No numerical data were available for patients affected by aortic dissection regarding the genes NOTCH1 and MFAP5, and patients with MAT2A mutation did not experience aortic dissections.^1^, ^36^, ^43^

The techniques used in the genetic screening, the identified genes, and genetic mutations are listed in Table S8. Genetic screening was employed as the sole screening modality in 26 (49%) studies and in combination with imaging modalities in 21 (40%). A total of 14 known genes were identified as a causative mutation for NS‐TADs, while 3 mapped loci without an identified gene were also found (Table 3 and Table S11). Single‐gene testing was used in 24 (45%) studies, comprehensive genomic sequencing in 14 (26%), and a combination of the 2 approaches in 7 (13%), respectively (Figure 2, Tables S8 and S10).

**Table 3:** Genetic Mutations and Correlations With Age and Size at Dissection^a^

Study (Author/Y)	Patients Affected (Aneurysm+Dissection)		Aortic Dissection								
	No.	%	Patients, No.	Patients, %	Male No.	Male, %	Age at Dissection, y	Range, y	Size at Dissection, mm	Range, mm	Patients Available for Analysis
ACTA 2											
Chamney et al 2015^24^	6	43	3	50	3	100	49±10.4^e^	37–55	n/a	···	···
Disabella et al 2011^25^	15	41	9	60	5	56	49.3±16.3^e^	29–73	59.1±22.3^e^	41–95	7
Guo et al 2007^32^	53	25	45	85	23	51	37.3±13.9^e^	13–67	61.1±15.0^e^	45–100	12
Morisaki et al 2009^52^	14	30	11	79	9	82	36.8±10.1^e^	25–52	n/a	···	···
Renard et al 2013^58^	26	32	13	79	7	54	40.7±15.4^e^	27–70	n/a	···	···
Ware et al 2014^69^	2	20	2	100	2	100	17	···	53±7.1^e^	48–58	2
Yoo et al 2010^72^	5	25	5	100	1	20	32.5±12.9^e^	20–46	35	···	1
FBN1											
Francke et al 1995^28^	10	38	3	30	2	67	55±14.1^e^	45–65	n/a	···	···
Regalado et al 2016^37^	15	52	8	53	4	50	32.3±9.9^e^	18–50	44	···	1
FOXE3											
Kuang et al 2016^47^	11	28	11	100	11	100	44.3±22.6^e^	9–88	n/a	···	···
LOX											
Guo et al 2016^37^	21	32	4	19	4	100	44.8±15.1^e^	25–60	n/a	···	···
MYH11											
Harakalova et al 2013^40^	15	20	10	67	7	70	46.6±19.5^e^	18–70	58.5±17.3^e^	44–65	4
Khau Van Kien et al 2005^46^	8	9	4	50	3	75	n/a	···	n/a	···	···
Pannu et al 2008^56^	6	22	5	83	4	80	45±8.8^e^	37–56	44	···	1
Renard et al 2013^58^	3	20	2	83	1	50	48.0±21.2^e^	33–63	n/a	···	···
Takeda et al 2015^61^	5	29	4	80	4	100	47.8±16.6^e^	32–70	n/a	···	···
Zhu et al 2006^73^	8	16	3	38	2	67	n/a	···	37.3±7.8^b^	n/a	2
MYLK											
Hannuksela et al 2016^39^	6	13	6	100	5	83	53.2±21.1^e^	23–75	47.5±0.7^e^	47–48	2
Wang et al 2010^66^	10	21	10	100	5	50	54.3±20.8^e^	16–78	40	···	1
Ziganshin et al 2015^74^	4	15	3	75	1	33	n/a	···	n/a	···	···
PRKG1											
Gago‐Diaz et al 2016^30^	11	37	6	55	3	50	34.2±12.9^e^	15–48	43±1.4^e^	42–44	2
Guo et al 2013^35^	37	42	22	59	10	45	31.1±10.3^e^	17–51	47±14.1^e^	37–57	2
SMAD3											
Regalado et al 2011^56^	23	22	14	61	n/a	n/a	42^c^	25–54	50	50	1
TGFB2											
Gago‐Diaz et al 2014^29^	6	19	2	33	2	100	37.5±4.9^e^	34–41	n/a	···	···
TGFBR1											
Dong et al 2014^27^	9	14	3	33	3	100	39±6.9^e^	35–47	51.3±17.9^e^	40–72	3
Tran‐Fadulo et al 2009^64^	29	37	14	48	10	71	25.6±14.3 (male)^d^ 38.6±9.7 (female)	14–62	90.6±42.7^e^	65–140	2
Ziganshin et al 2015^74^	7	41	3	43	2	67	n/a	···	n/a	···	···
TGFBR2											
Hasham et al 2003^41^	17	25	8	47	6	75	45.4±21.5^e^	14–72	n/a	···	···
Pannu et al 2005^53^	59	25	32	54	22	69	46.1±16.3^e^	14–73	n/a	···	···
Tran‐Fadulo et al 2009^65^	n/a	···	n/a	···	n/a	···	42.6±17.8 (male)^d^ 51.3±17.1 (female)	n/a	44±2.8^e^	42–46	2

n/a indicates not available.

a No data available for patients affected by aortic dissection regarding the genes NOTCH1 (reference 22) and MFAP5 (reference 1), and patients with MAT2A mutation did not experience aortic dissections (reference 15).

b Data available for dissection of the descending thoracic aorta only.

c Average age onset of dissection as presented by the authors.

d Derived from the entire cohort of patients with TGFBR1 and TGFBR2 mutations.

e Expressed as mean±SD.

The inheritance mode was essentially autosomal dominant (Table 1). Forty‐one (79%) studies reported on the penetrance of the NS‐TAD. Penetrance varied in relationship to the NS‐TAD form, with an average of 67% (study range: 20–100%) and was lower in females (Table 2). The age at dissection varied according to the underlying NS‐TAD form, with a mean age of presentation of 32 years for the familial thoracic aortic aneurysm and dissection forms associated with the mutations of the PRKG1 gene and of 54 years for those associated with the mutation of the MYLK gene (Figure 2 and Table 3). Ascending aortic diameters at the time of acute dissection were not reported for most of the individuals. Where this was reported, individuals affected by NS‐TADs showed stratification of the diameter of the thoracic aorta (aortic root, mid ascending, or descending aorta) at dissection and in the risk of progression to dissection by genetic mutation: from <4.5 cm for FBN1, FOXE2, MILK, PRKG1, SMAD3, TGFB2, TGFBR1, and TGFBR2, to >5.5 cm for ACTA2, LOX, and MYH11, respectively (Figure 2, Table S10). The identified NS‐TAD forms presented specific characteristics based on the causative genetic mutation (Tables 1 and 3, Table S10, and Figure S2).

### Extra‐Aortic Manifestations of NS‐TAD

Concomitant cardiovascular diseases were diagnosed in 11% of the relatives undergoing screening, while concomitant physical abnormalities were observed in 18% of the cases. Full details of all described external physical features and abnormalities of other organ systems are reported in Table S6.

### Resource Use and Cost‐Effectiveness

No information about resource use and the cost‐effectiveness of screening program in relatives of patients with NS‐TAD was reported in any of the identified studies. No studies address the psychological effect of screening in patients and their relative or its impact on quality of life of these families.

## Discussion

### Main Findings

The present study has identified an area of unmet clinical need with respect to screening of relatives of patients with NS‐TAD: familial NS‐TADs occur more frequently than previously recognized, affecting ≈30% of relatives with a male predominance (3:1). These are primarily inherited as single gene mutations, expressed in an autosomal dominant pattern with incomplete penetrance, which demonstrate variable expression with respect to age of presentation, sex, aneurysmal location, and aortic diameter before dissection. The risk of acute aortic syndrome is determined by the underlying genetic mutation and this risk extends not only to FDRs but also to SDRs and TDRs of patients affected by NS‐TADs. There is an overlap between nonsyndromic and syndromic TADs for some genetic mutations, as well as concomitant cardiovascular pathology in over 10% of screened patients. The review also identified knowledge gaps with respect to the predictive accuracy of commonly used screening tests across NS‐TAD populations, the optimal structure and extent of a screening program across families, and the effectiveness of a screening program with respect to clinical outcomes or cost.

### Clinical Implications

Nonsyndromic aortopathies have poor prognosis if untreated and the lack of relevant physical features precludes identification based on a clinical characteristics alone.^7^, ^75^ As a consequence, NS‐TADs are asymptomatic, alerting clinicians to the underlining aortopathy only when sudden death or an acute aortic dissection occurs.^7^, ^19^, ^72^, ^75^ This review indicates that routine screening and surveillance programs in relatives of patients affected by NS‐TADs, similar to those of syndromic TAD, are likely to identify significant numbers of patients with asymptomatic NS‐TAD.^4^, ^5^, ^76^, ^77^ The overlap in genetic mutations between NS‐TAD and syndromic TAD identified in the review further support this assertion. It follows that diagnosis, surveillance, and treatment of NS‐TADs before clinical presentation, as is the standard of care for syndromic TAD, is likely to reduce premature deaths. The findings of this article also indicate that current guidelines which recommend treatment based predominantly on the aortic diameter are likely to result in the undertreatment of NS‐TADs.^4^, ^5^ Specifically, subtypes of NS‐TADs attributed to specific genetic mutations may progress to aortic dissection without aneurysm formation.^78^ Here, the treatment of affected relatives stratified by NS‐TAD subtype and genetic abnormality are likely to result in further clinical benefits (Figure 2).

**Figure 3:**
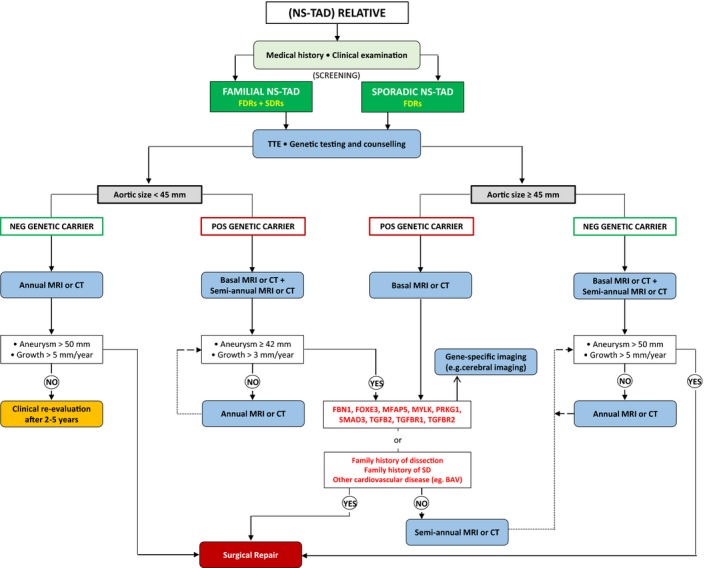
Proposed flow chart for a dedicated screening program for relatives of patients affected by nonsyndromic diseases of the thoracic aorta based on the authors’ extensive literature review. The figure represents the best judgement of the authors. BAV indicates bicuspid aortic valve; CT, computed tomography; FDRs, first‐degree relatives; MRI, magnetic resonance imaging; NS‐TAD, nonsyndromic thoracic aortic disease; SDRs, second‐degree relatives; TTE, transthoracic echocardiogram.

In addition to defining an area of unmet need, the review has identified important knowledge gaps with respect to screening. Specifically, the diagnostic accuracy of existing screening tests, the optimal screening program, and the clinical, societal, or economic benefits of such a screening program in the relatives of patients with sporadic or familial NS‐TAD are unclear. Current guidelines for the diagnosis and treatment of aortic diseases do not specify the details of what screening tests should be used (Table S11).^4^, ^5^, ^77^ The 2014 European Society of Cardiology guidelines recommend investigating FDRs by genetic counseling for family investigation and molecular testing, with a 5‐year interval screening until diagnosis (clinical or molecular) is established or ruled out (class I, level of evidence C).^5^ The corresponding 2010 American guidelines suggest aortic imaging screening for FDRs along with counseling and testing whether a specific mutant gene (FBN1, TGFBR1, TGFBR2, COL3A1, ACTA2, MYH11) is identified in the affected probands (class I, level of evidence C).^4^ These recommendations are based on opinion of the experts and small group studies only.^4^, ^5^ Importantly, specific testing schedules, the requirement for screening of SDRs and TDRs, the need for sequencing of other less‐common mutant genes, the optimal screening interval and modality, or the need to investigate the entire arterial tree other than the thoracic aorta are not specified.^4^, ^5^ The results of the current study suggest that FDRs, SDRs, and possibly TDRs should be offered screening for TAD. Clarification of the family history regarding the location of the aortic disease, the specific nature of “sudden deaths,” or the presence of other concomitant cardiovascular disorders during clinical examination should represent the initial step of screening.^75^ Our results also suggest that genetic testing and cardiac imaging with at least TTE should be offered to all FDRs and SDRs of patients with suspected NS‐TADs. Mutation carriers should undergo further imaging (MRI or CT scan), focusing on thoracic aorta and/or other arterial trees based on the causative gene mutation.^22^, ^23^, ^24^, ^25^, ^26^, ^27^, ^28^, ^29^, ^30^, ^31^, ^32^, ^33^, ^34^, ^35^, ^36^, ^37^, ^38^, ^39^, ^40^, ^41^, ^42^, ^43^, ^44^, ^45^, ^46^, ^47^, ^48^, ^49^, ^50^, ^51^, ^52^, ^53^, ^54^, ^55^, ^56^, ^57^, ^58^, ^59^, ^60^, ^61^, ^62^, ^63^, ^64^, ^65^, ^66^, ^67^, ^68^, ^69^, ^70^, ^71^, ^72^, ^73^, ^74^ For example, ACTA2‐mutation carriers should be monitored for coronary artery disease and occlusive cerebrovascular disease, in addition to the currently recommended routine imaging tests.^32^ We suggest that complete aortic imaging at initial diagnosis and at 6 months for patients with a confirmed genetic aortopathy (eg, FBN1, FOXE3, MFAP5, MYLK, PRKG1, SMAD3, TGFB2, TGFBR1, and TGFBR2) should be obtained to establish whether aortic enlargement is occurring.^4^, ^74^ The final clinical management of patients with a specific gene mutation could be modified on the basis of these data, enabling personalized treatment that is independent of the native aortic diameters.^4^, ^5^, ^41^, ^50^ Only relatives in whom a causal mutation is excluded and the aortic size is within recommended diameters should reasonably undergo clinical and/or imaging screening every 2 to 5 years, until diagnosis is confirmed or ruled out.^5^, ^76^ The appropriate temporal interval for follow‐up imaging, as well as the starting age for aortic surveillance, are also poorly defined. Generally, patients with NS‐TAD are diagnosed on average 10 years older than patients affected by syndromic aortopathies, being also characterized by a lower annual aortic dilatation progression (0.5–0.7 mm/y).^59^, ^60^ It therefore seems reasonable to initiate the screening 15 to 10 years earlier than first aneurysm or when dissection or sudden death is recorded within the family.^60^, ^79^ A screening pathway based on these observations is proposed in Figure 3.

There are several additional factors that may influence our proposed screening algorithm. First, variable penetrance, which often characterizes NS‐TAD forms, is a potential confounder. This results in intrafamilial variability, which is evident not only with reference to the aortopathy itself (severity, age of onset), but also with regard to other phenotypic manifestations.^65^, ^66^, ^67^, ^68^, ^69^, ^70^, ^71^, ^72^, ^73^, ^74^, ^75^, ^76^, ^77^, ^78^, ^79^, ^80^, ^81^ The presence of associated features is certainly suggestive of having inherited the aortic condition along with the predisposition to the aortopathy, but the absence of these associated features does not eliminate the risk of having an underlying aortopathy. Second, women often demonstrate a lower lifetime risk of aortopathy, developing the condition at a later age than men.^81^ This phenomenon, known as sexual dimorphism, explains the apparent paradox of an affected teenager with an affected maternal grandfather but an unaffected mother with normal echocardiographic features. Third, the age at onset of the aortopathy may be important in the natural history of the disease. Ma et al^82^ recently demonstrated that age at onset of aortic dissection is lower in families with a positive history for aortic dissection, therefore suggesting a prompt and more aggressive screening pathway in these families. A positive family history with an aortopathy occurring at younger ages confers a significantly increased risk of developing a new dissection in apparently unaffected family members.^81^ The above findings are all important in guiding the proper screening and surveillance strategies.

### Study Limitations

The most important limitation of the review is the uncertainty regarding the likely yield of new cases if a screening program were to be implemented. The studies identified in our searches were predominantly studies of familial aortopathy, and the prevalence of TAD in these populations will not reflect that for NS‐TAD overall. Conversely, sporadic NS‐TAD, which constitutes the majority (80%) of all cases also has a genetic component.^7^ Roberston et al^59^ investigating 321 NS‐TAD probands who had no family history of TAD identified 27% of newly affected relatives. It is likely that these patients are carriers of a de novo mutation, making these “sporadic” patients founders of a new nonsyndromic aortopathy. For example, recent studies have identified gene deletions and uniparental disomy, and genetic variations in LRP1 and ULK4 in sporadic NS‐TAD.^83^ This suggests that the relatives of patients affected by both familial and sporadic NS‐TADs may benefit from screening. It also argues for use of nontargeted genetic screening tests such as exome or whole genome sequencing that will detect de novo or as‐yet unrecognized common mutations. A second limitation is that there is currently no evidence to inform secondary prevention or intervention strategies in newly diagnosed NS‐TAD, particularly where the aorta is phenotypically normal. Although the evidence presented supports the routine implementation of combined imaging and genetic testing in relatives of patients with NS‐TAD, no study has proven that stratified treatment, independent of the native aortic diameter, will save lives. However, the stratified treatment of syndromic TAD is common practice, as this is known to prevent deaths from aortic disease. We suggest that the results of this review support the extension of similar programs to all patients with TAD. To address these limitations, we propose that further research should first establish the true prevalence of genetic abnormalities and phenotypic disease diagnosed by screening (genetic testing and imaging) all FDRs and SDRs of patients with both familial and sporadic NS‐TAD. Further studies will be required to address uncertainty with respect to effectiveness, psychological impact, and the costs of lifelong screening in these groups. Finally, the heterogeneity of the included studies, the large period of publication across 3 decades, and the familial‐based approach have limited our ability to analyze the impact of region or ethnicity in the risk of aortopathies and the related screening strategy.

## Conclusions

The findings of this review support routine imaging and genetic testing of relatives of patients with nonsyndromic aortopathies. The evidence suggests that screening of FDRs and SDRs of patients affected by familial NS‐TAD and FDRs of those affected by sporadic NS‐TADs will result in significant numbers of patients with otherwise undiagnosed disease. Personalized screening programs determined by the subtype of NS‐TAD and its related genetic mutation have the potential to benefit these patients. However, the diagnostic yield of available screening tests is unclear, as are the details of a screening program, and there is no existing evidence that routine screening and stratified treatment will have clinical or economic benefits. Further studies are required to address these knowledge gaps.

## Author Contributions

Mariscalco and Debiec had full access to all of the data in the study and take responsibility for the integrity of the data and the accuracy of the data analysis. Study concept and design: Mariscalco, Debiec, Samani, and Murphy. Acquisition of data: Mariscalco and Debiec. Analysis and interpretation of data: Mariscalco, Debiec, Samani, and Murphy. Drafting of the article: Mariscalco, Elefteriades, Samani, and Murphy. Critical revision of the article for important intellectual content: Mariscalco, Debiec, Elefteriades, Samani, and Murphy. Article supervision: Mariscalco, Elefteriades, Samani, and Murphy. Statistical analysis: Mariscalco.

## Disclosures

Mariscalco declares support from Vascutek, an aortic prosthesis manufacturer, to attend scientific meetings. Murphy declares support from BHF chair of cardiac surgery, Vascutek for attendance at scientific meetings and financial support for educational activities. The remaining authors have no disclosures to report.

## Supporting information


**Data S1.** Systematic review protocol.
**Table S1.** PRISMA Checklist of Items to Include When Reporting a Systematic Review or Meta‐Analysis*
**Table S2.** PICOS Criteria for Inclusion and Exclusion of Studies Into Meta‐Analysis
**Table S3.** Full Details of the Screened Family Relatives With Number and ID of the Included Families
**Table S4.** Full Details of the Family Pedigree, Eligible, Screened, and Affected Patients and Relatives
**Table S5.** Full Details of the FDRs, SDRs, and TDRs of Evaluated Probands
**Table S6.** Full Details of the Screened Families and Relatives With Reference to Additional Observed Cardiovascular Diseases and Physical Features
**Table S7.** Details of the Adopted Imaging Modalities for the Screening of Relatives
**Table S8.** Details of the Adopted Screening Modalities in the Included Studies
**Table S9.** Quality Assessment of the Included Studies
**Table S10.** Genetic Architecture of Thoracic Aortic Diseases in Nonsyndromic Forms After Screening of the Family Relatives
**Table S11.** Current Guidelines for Diagnosis and Treatment of Aortic Diseases
**Figure S1.** PRISMA (Preferred Reporting Items for Systematic Reviews and Meta‐Analyses) flow diagram of search strategy (through December 31, 2017).
**Figure S2.** Genes with established causative association with nonsyndromic thoracic aortic aneurysms and dissection identified in the present systematic review.
