# Supplementary material for: Systematic Review of Studies That Have Evaluated Screening Tests in Relatives of Patients Affected by Nonsyndromic Thoracic Aortic Disease
Source: J Am Heart Assoc. 2018 Jul 31;7(15):e009302. doi: 10.1161/JAHA.118.009302 (PMC6201478; doi:10.1161/JAHA.118.009302)
Supplement: Supplementary file 1 — Data S1. Systematic review protocol. Table S1. PRISMA Checklist of Items to Include When Reporting a Systematic Review or Meta‐Analysis* Table S2. PICOS Criteria for Inclusion and Exclusion of Studies Into Meta‐Analysis Table S3. Full Details of the Screened Family Relatives With Number and ID of the Included Families Table S4. Full Details of the Family Pedigree, Eligible, Screened, and Affected Patients and Relatives Table S5. Full Details of the FDRs, SDRs, and TDRs of Evaluated Probands Table S6. Full Details of the Screened Families and Relatives With Reference to Additional Observed Cardiovascular Diseases and Physical Features Table S7. Details of the Adopted Imaging Modalities for the Screening of Relatives Table S8. Details of the Adopted Screening Modalities in the Included Studies Table S9. Quality Assessment of the Included Studies Table S10. Genetic Architecture of Thoracic Aortic Diseases in Nonsyndromic Forms After Screening of the Family Relatives Table S11. Current Guidelines for Diagnosis and Treatment of Aortic Diseases Figure S1. PRISMA (Preferred Reporting Items for Systematic Reviews and Meta‐Analyses) flow diagram of search strategy (through December 31, 2017). Figure S2. Genes with established causative association with nonsyndromic thoracic aortic aneurysms and dissection identified in the present systematic review. [file JAH3-7-e009302-s001.pdf]

SUPPLEMENTAL MATERIAL

Data S1. Systematic review protocol

A Systematic Review of Screening of Relatives of Patients with Non-Syndromic Thoracic Aortic Diseases

Version No:	Version 0.5
Date:	30 June 2017
Sponsor:	University of Leicester
Funder:	N/A
Type of study:	**Systematic Review (Qualitative)**
PROSPERO n.	CRD42017064598 (2017)

UNIVERSITY OF LEICESTER

Authors

**Dr Giovanni Mariscalco**<br>Associate Professor in Cardiac Surgery<br>University of Leicester • Glenfield Hospital<br>Clinical Sciences Wing<br>Leicester, LE3 9QP<br>Tel: 0116 258 3019<br>Fax: 0116 287 5792<br>Email: gm247@leicester.ac.uk	**Dr Radoslaw M. Debiec**<br>Clinical Research Fellow in Cardiology<br>University of Leicester • Glenfield Hospital<br>Clinical Sciences Wing<br>Leicester, LE3 9QP<br>Tel: 0116 204 4776<br>Email: rmd24@le.ac.uk
**Prof Gavin J. Murphy**<br>British Heart Foundation,<br>Professor of Cardiac Surgery<br>University of Leicester • Glenfield Hospital<br>Clinical Sciences Wing<br>Leicester, LE3 9QP<br>Tel: 0116 258 3054<br>Email: gjm19@le.ac.uk	

INDEX

**1. PROTOCOL INFORMATION**	**Pag**	**3**
1.1. Contact person		3
1.2. Conflict of interest		3
1.3. Funding sources/Sponsor		3
1.4. Dates		3
1.5. Type of review		3
1.6. Language		3
1.7. Country		3
1.8. Keywords		3
**2. GLOSSARY/ABBREVIATIONS**	**Pag**	**3**
**3. BACKGROUND AND RATIONALE**	**Pag**	**4**
3.1. Rationale		4
3.2. Key Points		4
3.3. Description of the condition and the intervention		4
3.3.1. Epidemiology and Outcomes of TAD		4
3.3.2. Forms of TAD		6
3.3.3. Imaging modality for screening TAD		6
3.3.4. Genetic screening for TAD		7
3.3.5. The knowledge gap		8
3.3.6. Why it is important to do this review		8
**4. OBJECTIVES**	**Pag**	**8**
4.1. Hypothesis		8
4.2. Aims		9
**5. METHODS**	**Pag**	**9**
5.1. Criteria for selecting studies		9
5.1.1. Types of studies		9
5.1.2. Study exclusion criteria		9
5.1.3. Types of participants		9
5.1.4. Variable definitions		9
5.1.5. Exposures of Interest		10
5.1.6. Types of outcome measures		10
5.2. Search methods for identification of studies		10
5.2.1. Search strategy		10
5.2.2. Searching other resources		11
5.2.3. Results of the scoping search		11
5.3. Data collection		11
5.3.1. Selection of studies (screening-eligibility-inclusion)		11
5.3.2. Qualitative analysis		11
5.3.3. Data extraction and management		11
5.5. Measures of treatment effect and data analysis		12
5.5.1. Measures and data representation		12
5.5.2. Data analysis		12
**6. COMPETING INTEREST**	**Pag**	**12**
**8. REFERENCES**	**Pag**	**12**

1. PROTOCOL INFORMATION

1.1. Contact person

Dr Giovanni Mariscalco
Associate Professor in Cardiac Surgery
University of Leicester • Glenfield Hospital
Clinical Sciences Wing
Leicester, LE3 9QP
Tel: 0116 258 3019
Fax: 0116 287 5792
Email: gm247@leicester.ac.uk

1.2. Conflict of interest

None

1.3. Founding Sources/Sponsor

University of Leicester

1.4. Dates

- Start date: 1 January 2017
- Anticipated completion date: 31 July 2017

1.5. Type of review

Epidemiologic; Intervention

1.6. Language

English

1.7. Country

United Kingdom

1.8. Keywords

Systematic review; aorta, thoracic; aortic aneurysms; aortic dissection; relatives; siblings; pedigree; humans; screening; echocardiography; sporadic thoracic aorta; cardiac surgery.

2. GLOSSARY/ABBREVIATIONS

BAV	Bicuspid Aortic Valve
CT	Computed Tomography
LDS	Loeys-Dietz Syndrome
MFS	Marfan Syndrome
MRI	magnetic Resonance Imaging
NS-TAD	Non-syndromic Thoracic Aortic Disease
OMIN	Online Mendelian Inheritance in Man
TAA	Thoracic Aortic Aneurysms
TAD	Thoracic Aortic Disease
TADA	Thoracic Aortic Acute Dissection
TEVAR	Transaortic Endovascular Aortic repair
TOE	Transoesophageal Echocardiogram
TTE	Transthoracic Echocardiogram

3. BACKGROUND AND RATIONALE

3.1. Rationale

Recent guidelines on diagnosis and management of thoracic aorta disease (TAD) have identified a knowledge gap with respect to the most effective screening modality for relatives of patients affected by non-syndromic TAD. Previous research has established a specific and clear screening pathway for syndromic TAD forms, including Marfan (MFS) and Loeys-Dietz (LDS) syndromes, and other similar connective tissue diseases. Considering the incidence of NS-TAD and the impact of prompt diagnosis in improving clinical outcomes in TAD, we attempted to analyse the existing evidence that relates to screening modality and programs in relatives of patients affected by non-syndromic TAD.

3.2. Key points

Thoracic aortic disease is a term that essentially refers to an interrelated collection of pathologies that include thoracic aortic aneurysms (TAA) and aortic dissections (TADA).[1,2] TAA is often silent and commonly present as life-threatening emergencies, referred to as acute aortic syndromes.[1,2] In the United Kingdom (UK), over 6,500 deaths are attributable to TAD every year, and this number is increasing.[3,4] Attempts to formulate consensus statements and relevant guidelines have identified significant gaps in the knowledge with respect to the pathogenesis, appropriate management of, and configuration of clinical services for optimal treatment of aortic disease. This results in high variation in the diagnosis and management approach and regional differences in the quality of care and outcomes.[1,2,4] In particular methodology and modalities for genetic or imaging familial screening sof relatives of patients with non-syndromic forms of TAD (NS-TAD) are not well established.[1,2] To address this knowledge gap, we propose to undertake a systematic review of the existing literature that relates to genetic and/or imaging screening undertaken in relatives of patients with NS-TAD (diagnosed and/or operated on). Secondary aims are also to determine the effectiveness of screening in relatives of NS-TAD patients, and to catalogue existing evidence on genetic association in non-syndromic TAD.

3.3. Description of the condition and the intervention

3.3.1. Epidemiology and outcomes of TAD

The term “thoracic aortic disease” includes a wide range of aortic diseases with variable clinical presentations and prognosis. The Global Burden of Disease 2010 project demonstrated that the overall global death rate from aortic aneurysms and aortic dissection increased from 2.49 per 100000 to 2.78 per 100000 inhabitants between 1990 and 2010, with higher rates for men.[5] At the same time, admissions for thoracic aortic aneurysms have increased from 4.4 to 9.0 per 100000 in the UK, mainly due to an increase in proportion of elderly patients, over 75 years of age.[3] The epidemiology of TAD is difficult to establish since aortic diseases may be diagnosed after a long period of subclinical development or they may have an acute fatal presentation. In addition, the natural history of TAD remains poorly understood, and errors in the diagnostic process may account for deaths otherwise attributed to other diseases such as myocardial infarction or pulmonary embolism. TADs are usually asymptomatic until an acute complication occurs, requiring a prompt diagnosis and treatment in specialized centres. Management of TAD is complex and dictated by the size, extent and location of the disease condition as well as the underlying pathology (aneurysm or dissection). Options include conservative medical therapy (e.g. oral hypotensive agents such as beta blockers, ace-inhibitors, diuretics or statins) open surgical intervention, thoracic endovascular aortic repair (TEVAR), or hybrid procedures including epiaortic vessel debranching.[1,2]

Early and late results also vary across centres and countries. In Europe and the wider world, mortality rates for operated type A acute aortic dissection range from 12% to 42%.[4,6-8] However, in some high-volume USA centres the mortality rate is lower, ranging from to 2 to 10%.[9,10] On the other hand, hospital mortality from elective nondissection surgery on the thoracic aorta ranges from 5% to 10%.[11] For patients suffering from an acute type B aortic dissection, mortality rates for medical treatment approach, endovascular and open surgical repair range from 3% to 20%.[12,13] Table 1 summarizes early and long-term mortality for treated TADs.

Table 1. Epidemiology and outcomes of treated TAD

	Operation/ Disease	Hospital mortality (30-day)	Mortality (Kaplan-Meier)	Some complications	Description	Re-op	Ref
1	Bentall procedure: Composite valve & graft replacement of ascending aorta and aortic valve	0%	9.9%±4.8% at 8 years	9% post-op Thromboembolic event	MFS, n=56, mean age 38	2%	(14)
		2.6%	10.4% ± 3.4% at 10 years	3.7% post-op Thromboembolic event	N= 195, mostly annuloaortic ectasia (54.4%), ascending aortic aneurysm 26.2%		(15)
2	Separate ascending aorta and aortic valve replacements	2%	31% at 5 years		N=50, mean age 65	0	(16)
3	Valve-sparing aortic root reconstruction	0%	0% at 8 years	1% post-op Thromboembolic event	MFS, n=84, mean age 29	6%	(14)
		1.3%	17% ±5% at 8 years	3% post-op Thromboembolic event	N= 151, Aortic root aneurysms	1%	(17)
4	Ascending aorta alone	0%	0% at 5 years		N = 21	0	(16)
5	Acute Type A Aortic Dissection (operated)	26 %	n.a.	The risk of death after surgical repair of acute aortic dissection is strongly influenced by associated stroke, mesenteric ischemia, renal failure, and myocardial ischemia	N=208	n/a	(18)
		22%	5.1% ±1.2% at 5 years 11.9%±2.6 % at 10 years		N=487	n/a	(19)
	Acute Type A Aortic Dissection (not operated)	58%	n/a		N=81	n/a	(18)
	Type B Aortic Dissection (operated)	31.4%	n/a		N=35	n/a	(18)
	Type B Aortic Dissection (not operated)	10.7%	n/a		N=140	n/a	(18)
6	Arch replacement	8.9% (elective 6.0%)	n/a	stroke rate 8.4% (elective 6.9%)	N= 347, (elective 232)	n/a	(20)
7	Descending aorta replacement	7.1%	13% at 1 year 28% at 5 years	unruptured	N=11565	n/a	(21)
		45.6%	74% at 5 yrs	ruptured	N=1307	n/a	(21)
		6.1%	18% at 1 year 62% at 5	Although perioperative mortality is lower with TEVAR, Medicare	N=2433	n/a	(55)

			years	patients selected for TEVAR have worse long-term survival than patients selected for open repair.			
8	TEVAR to descending thoracic aorta (unruptured)	28.4%	77% at 5 years	Although perioperative mortality is lower with TEVAR, Medicare patients selected for TEVAR have worse long-term survival than patients selected for open repair.	N=299	n/a	(21)
9	TEVAR to descending thoracic aorta (ruptured)						

3.3.2. Forms of TAD

Currently TAD can be subdivided in two main entities:

1) Syndromic TAD
2) Non-syndromic TAD (NS-TAD)

Up to 20% of individuals with TAD who do not present pathognomonic features of syndromic forms (especially MFS or LDS), have a family history of TAA and/or TADA.[22] Syndromic forms of TAD are associated with abnormalities of other organs, while those non-syndromic present manifestations limited to the thoracic aorta only. NS-TAD includes two distinct sub-groups: the familial (more than one family member is affected) and the sporadic TAD forms.[22] Table 2 summarizes syndromic and non-syndromic forms of TAD.[23]

Table 2. Syndromic and non-syndromic aneurysms conditions

**Syndromic Aneurysms Conditions**	**Non-syndromic Aneurysm Conditions**
MFS (Marfan syndrome)	FTAAD
LDS (Loeys-Dietz syndrome)	
Vascular Ehlers-Danlos syndrome	Familial TAA
Shprintzen-Goldberg syndrome	
Aneurysms-osteoarthritis syndrome	BAV with thoracic aortic aneurysm
Cutis laxa with aneurysm	

A genetic predisposition to the development of TAD in non-syndromic forms has been documented in 19% of patients, and patients with familial TAD are younger at the time of diagnosis that those with sporadic forms, but older when compared to syndromic TAD forms.[22] Previous studies have also suggested that 20% of NS-TAD patients referred for surgery have first-degree relatives similarly affected.[22,24]

In majority of patients the familial NS-TAD is inherited as an autosomal-dominant disorder with decreased penetrance and variable expression. Several genes have been demonstrated to be involved NS-TAD (Table 3).[23,25,26]

3.3.3. Imaging modality for screening TAD

Imaging techniques play a crucial role in the diagnosis, follow-up and management of TAD. Ultrasound, including transthoracic (TTE) and transoesophageal (TOE) echocardiograms, computed tomography (CT) and magnetic resonance (MR) can be used for the assessment of aneurysms and dissections located in the different segments of the thoracic aorta. All these imaging modalities have their strengths and limitations, and no single imaging modality has a perfect resolution (Table 4).[1,2,27]

The preferred imaging modality for screening of TAD has not yet been recommended in the international guidelines (ESC, AHA), and a variable combinations of imaging modalities at baseline and during follow-up have been reported. In addition, relationship between genetic and imaging screening modalities has not been elucidated in relatives of patients with NS-TAD.[1,2]

**Table 3. genes associated with NS-TAD forms**	
**Gene (protein)**	**OMIN N.**
Extracellular Matrix proteins	
FBN1 (fibrillin-1)	154700
COLA3A1 (Collagen 3 α-1)	130050
LOX (lysyl oxidase)	Unassigned
MFAP5 (microfibrillar associated protein 5)	616166
TGF-β pathway	
TGFBR1 (transforming growth factor-β receptor 1)	609192
TGFBR2 (transforming growth factor-β receptor 2)	610168
SMAD2 (SMAD family member 2)	Unassigned
Cytoskeletal/smooth muscle contraction apparatus proteins	
ACTA2 (α-smooth muscle actin)	611788
MYH11 (smooth muscle myosin)	132900
MYLK (myosin light chain kinase)	613780
PRKG1 (protein kinase, cGMP-dependent, type I)	615436
Neural crest migration	
NOTCH1 (notch1)	109730
Unknown	
MAT2A (methionine adenosyl-transferase II, α)	Unassigned
FOXE3 (forkhead box 3)	Unassigned

3.3.4. Genetic screening for TAD

Establishing a specific genetic cause of NS-TAD is of paramount importance for defining the most appropriate management for the relatives of affected patients. Risk assessment and surveillance as well recommendations for specific medical and surgical management are based on the gene identification. Specific genes have been identified, each of them are involved in specific aortopathy pathways (Table 3). Multi-gene panel, single-gene testing and genomic sequencing all can be utilized as evaluation strategy to identify the genetic cause of NS-TAD formm.[26] For some genes, specific recommendations exist in order to tailor the most appropriate clinical and/or surgical intervention. In patients with ACTA 2 gene mutations, elective surgical repair is advisable when the diameter of the ascending aorta/aortic root reaches 4.5 cm;[28] for carriers of FBN1 gene mutations, operation should be considered when the diameter of the aneurysm reaches 5 cm;[2] fin cases with TGFBR1/TGFRB2 mutations surgical management should be anticipated when the aortic root diameter reaches 4.0 cm.[29]

Table 4. Characteristics of imaging modalities for TAD assessment (adapted from Evagelista A.)[27]				
**Variable**	**TTE**	**TOE**	**CTA**	**MRA**
Readily available	+++	+	+++	+
Quickly performed	+++	++	++	+
Non-invasive	+++	+	+++	+++
No iodinated contrast	+++	+++	-	+++
No radiation	+++	+++	-	+++
Dynamic and functional information	++	++	-	+++
Aortic wall visualization	+	++	+++	+++
Assessment of aortic root/ascending aorta	++	++	+++	+++
Assessment of aortic arch and carotid vessels	-	+	+++	+++
Assessment of descending aorta	-	++	+++	+++
Assessment of aortic valve	+++	+++	-	++
Assessment of left ventricle function	+++	+++	-	-
3D multiplanar and high resolution	-	-	+++	+++
Measurement accuracy	+	+	+++	++
Costs	+++	+++	+	-
Abbreviations: + limited; ++ good; +++ excellent; - bad. CTA, CT angiography, MRA, MR angiography				

3.3.5. The knowledge gap

The 2014 European Society of Cardiology (ESC) Guidelines for the management of NS-TAD include a level I recommendation for the screening of first-degree relatives of patients with TAA and /or TADA to identify those with asymptomatic disease, and for referring the patient to a geneticist for family investigation, once a familial NS-TAD from is recognized.[1] However, the evidence to support these recommendation is level C, based on the consensus of opinion of the experts, small and retrospective studies. Similarly, the American Heart Association (AHA) 2010 guidelines on screening for NS-TAD primarily consist of recommendations based on level C evidence.[2] This contrasts with the evidence-based for the screening modalities of other syndromic TAD conditions. In addition, screening of second-degree relatives of patients affected by NS-TAD and screening of other arterial district are not well established, presenting both a level IIa recommendation only.[1,2] In addition, no data about the effectiveness or cost-effectiveness of a screening program in relatives of NS-TAD patients are present, and indications for genetic analysis are not well established as well the preferred TAD imaging modality.[1,2]

3.3.6. Why it is important to do this review

Compared to syndromic TAD forms (i.e. Marfan or Loeys-Dietz syndromes) which are characterized by relevant physical features, therefore alerting clinicians to the underlying aortopathy, non-syndromic (NS) TAD forms lack of clear external physical signs, and are characterised by silent aneurysm formation and dissection.[22,24] Thoracic aortic disease (TAD) have high mortality, and early recognition is essential in order to establish a prompt clinical and surgical management,[1,2] therefore identifying as early as possible those who would benefit from prompt treatment and preventive measures.

4. OBJECTIVES

The overarching aim of the present review is to determine the effectiveness of screening of asymptomatic relatives of NS-TAD probands, highlighting the incidence and prevalence of TAD in this population. Secondary objectives will be to catalogue all screening modalities (both genetic and imaging) adopted in the above relatives, and to assess the effectiveness or cost-effectiveness of screening.

4.1. Hypothesis

It is our hypothesis that systematic screening of first- and second-degree relatives of patients affected by NS-TAD will provide a substantial benefit in identifying silent TAD and preventing related death.

Furthermore systematic review of the existing evidence may help with clarifying the best cost-effective screening modality or combination of modalities (genetic vs imaging) and/or imaging tools (TTE vs CT vs MRI), and may contribute to create a catalogue with all the known genetic markers associated with TAD.

4.2. Aims

The aims of the present review will be:

1. To summarise published studies that have considered the screening in relatives of patients with by NS-TAD;
2. To estimate the incidence and prevalence of TAD in family members of patients with NS-TAD of silent and undiagnosed disease of thoracic aorta (TAA and TADA);
3. To provide a defined screening strategy to identify potential individuals affected by TAD who will benefit the most from tailored clinical or surgical managements;
4. To provide a comprehensive list of genes, which can be utilized as risk assessment in family members of a proband with NS-TAD.

5. METHODS

5.1. Criteria for Selecting Studies

5.1.1. Types of studies

We will consider clinical studies that have performed genetic and/or imaging evaluation of relatives of patients affected by NS-TAD. The following types of studies will be analysed:

1. Clinical randomised trials;
2. Controlled before-and-after studies;
3. Prospective and retrospective cohort studies;
4. Cross-sectional studies;
5. Case-control studies;
6. Case series.

Study design features will be assessed according to established criteria from the Cochrane Handbook.[30] In addition, inclusion and exclusion criteria for qualitative and quantitative analyses will be presented according to PICOS criteria.

5.1.2. Study exclusion criteria

Exclusion criteria will include:

1. Studies where screening is based on clinical patient evaluation only;
2. Studies where screening does not include genetic patient evaluation and/or patients are not subjected to recognised imaging modality such as TTE/TOE, CT and MRI of the thoracic aorta;
3. Studies where screening is not based on prospective recruitment/analysis of the proband relatives;
4. Studies where screening involved patients without clear differentiation from syndromic forms;
5. Repeat publications of the same analysis or dataset;
6. Conference abstracts;
7. Editorials & opinion pieces;
8. Books or grey literature.

5.1.3. Types of participants

Relatives of probands with a diagnosis of NS-TAD, including aneurysm, aortic rupture, acute/chronic aortic dissection, intramural hematoma, and penetrating ulcer of the thoracic aorta.

5.1.4. Variable definitions

- <u>Familial non-syndromic TAD</u> will be defined as those occurring in patients having 1 or more first-generation relatives with an aortic aneurysm and no history of MFS or any other connective tissue disease (Table 2).[22]
- <u>Sporadic TAD</u> will be defined as those occurring in patients apparently without another relative with TAD.
- <u>Patients affected by TAD</u> will be considered in the entire family pedigree, and will be defined as those individuals having a diagnosis of TAD. Their percentage will be considered in the obtained family pedigree.

- Diagnosis of TAD (phenotype) will be considered if confirmed by imaging (TTE and/or CT and/or MRI), post-mortem examination or intraoperative findings. Sudden deaths will be excluded from TAD diagnosis.
- Percentage (%) of observed TAD will be calculated from the total number of relatives in the entire pedigree.
- Patients defined as eligible for screening (genetic and/or imaging) will include first- and second-degree relatives of a proband with NS-TAD; spouse and deceased patients will be included if blood/tissue samples were available for analysis.
- Patients screened will be defined as those having had prospective genetic screening and imaging studies (TTE and/or CT and/or MRI). Patient deceased will be included in the "patient screened category" if they had blood or tissue collected at the time of operation , which allowed for subsequent genetic analysis.
- Percentage (%) of screened patients will be calculated from the number of patients considered eligible for screening.
- Proband (index patient) will be defined as the first family member affected by NS-TAD. It will be denoted as shaded square (male) or circle (female) in the family pedigree marked by an arrow.
- Penetrance (%) will be defined as: $\frac{\text{n. of patients affected by TAD positive for the gene mutation}}{\text{Subjects with positive gene mutation}}$
- First-degree relatives (FDR) of the proband will include:
    1) Parents (father and mother)
    2) Child (daughter and son)
    3) Siblings (brother and sister).
- Second-degree relatives (SDR) will include:
    1) Grandparent
    2) Grandchild
    3) Aunt and uncle
    4) Nephew and niece.
- Third degree relatives (TDR) will include:
    1) Great-grandparent
    2) Great-grandchild
    3) Cousin.
- Thoracic aortic dissection (TADA) category will include type A and B acute or chronic forms as well as other acute aortic syndromes (rupture, intramural hematoma, penetrating ulcer).

5.1.5. Exposures of Interest

The primary exposure of interest will be a disease of the thoracic aorta (aneurysm and dissection).

5.1.6. Types of outcome measures

• The primary outcome will be new diagnosis of TAD, including aneurysms or dissections, in relatives of patients with NS-TAD forms.

• Secondary outcome will include:

a. Gender TAD preponderance;
b. Rate between TAA and TADA in the NS-TAD form;
c. Age at diagnosis of TADA;
d. Concomitant vascular/cardiac associated diseases;
e. Concomitant associated clinical features;
f. Genetic risk assessment with the penetrance of the NS-TAD form;
g. Cost-effectiveness of adopted imaging modality.

5.2. Search Methods for Identification of Studies

5.2.1. Search strategy

We will search the following databases (from inception to 31 December 2017):

1. Cochrane Library
2. PubMed/MEDLINE (1946 to 31 December 2017);
3. Embase (1974 to 31 December 2017);

No language restriction will be applied. We also anticipate that articles not in English will be translated using Google Translate® which is a free, Web-based program with a reputation for accurate, natural translation.[31,32]

5.2.2. Searching other resources

A systematic search in the Online Mendelian Inheritance in Man (OMIM) database (http://www.omim.org/) will be also performed through December 2017, using similar terms of the below literature search.
Finally, we will check references of all identified studies, relevant review articles, and current treatment guidelines for further literature. These searches will be limited to the 'first generation' reference lists.

5.2.3. Results of the scoping search

A preliminary scoping search (PUBMED) using the terms (aorta, thoracic) or (aortic aneurysm) or (aortic dissection) AND (relatives) or (pedigree) or (siblings) and (screening) and (humans) accounted for 1,022 sources.

5.3. Data collection

5.3.1. Selection of studies (screening-eligibility-inclusion)

Two authors (G.M. and D.R.) will screen all titles and abstracts of papers identified for relevance to the review aims (electronic search). An independent search with the review of all articles will be conducted by a third review (G.J.M.). Studies clearly not meeting the eligibility criteria will be excluded at this stage. Remaining studies will be assessed on the basis of their full text for inclusion or exclusion using the criteria indicated above. At this stage, two reviewers (G.M. and D.R.) will independently assess eligibility. Disagreements will be resolved by consensus in discussion with a third reviewer (G.J.M.). Numbers of studies assessed, included and excluded will be recorded. Duplicate reporting of studies will be carefully assessed and indicated.

5.3.2. Qualitative analysis

Two investigators independently will appraise all articles that will met inclusion criteria, and study quality will be assessed using the Newcastle-Ottawa Scale, and the U.S. Preventive Services Task Force (USPSTF).[33,34] Methdodological quality will be also assessed considering the Cochrane Risk of Bias toll.[35]
Disagreement about critical appraisal will be resolved by discussion. The qualitative analysis will help to explore questions such as how patient selection, treatment and type of study may have influenced the primary effect estimate. In addition, the following questions will be considered for a qualitative analysis:

1. Was the study population well described?
2. Were the outcomes of interest clearly defined?
3. Were the exposures of interest (primary and secondary) well defined?
4. Does the article state both inclusion and exclusion criteria?
5. Were the analysed variables clearly defined?
6. Was the screening prospectively conducted?
7. Were relatives prospectively invited and subject to screening (genetic and/or imaging)?

5.3.3. Data extraction and management

Two authors (G.M. and D.R.) will extract selected data from eligible studies, which will be subsequently checked by a third author (G.J.M.). The following data will be collected and tabulated with Microsoft Excel (Microsoft Corporation, Redmond, WA):

1. Study characteristics:

Author/authors; date of publication; country of origin including the university where the study was mainly carried out; inclusion/exclusion criteria.

2. Population characteristics:

Ethnic origin of the patient population; number of family enrolled in the screening program; identification of the family; number of subjects in the family pedigree; number of eligible individuals for screening purpose; number of screened relatives.

3. Exposures:

Rate of newly diagnosed relatives with TAD and/or TADA

4. Outcomes:

Rate of registered sudden death; age (years) at diagnosis for patients with TADA (mean and range); gender preponderance; rate and type of concomitant associated cardiac or vascular diseases; rate and type of concomitant associated clinical features; penetrance; identification of genetic mutation.

5. Screening modality:

Type of adopted genetic screening; type of imaging modality adopted for screened.

Two authors (G.M. and R.D.) will perform data extraction independently. Data will be extracted onto study specific data extraction form. Disagreements will be resolved by consensus between the authors or by discussion with a third author where necessary (G.J.M.). A second check of all data entry will be performed in order to avoid discrepancies. Missing data will be requested from study authors. If data are unclear, missing, or presented in a form that is unable to be reliably extracted, authors will be contacted to assist in the process. The corresponding author will be initially contacted by email, with the first author (if not the corresponding author) copied into all correspondence. If email addresses are not available, authors will be contacted by phone. Authors will be given seven days to respond to emails, after which they will be followed up with a phone call and an additional email. If no responses are received after an additional seven days, another phone call will be made to contact the author. Other attempt will occur for other seven days; thereafter the authors will be classified as uncontactable.

5.5. Measures of treatment effect and data analysis

5.5.1. Measures and data representation

A narrative synthesis of the included studies will be provided, focusing on the effectiveness of genetic and/or imaging in the new diagnosis of TAD, including aneurysms or dissections, in relatives of patients with NS-TAD forms. Detailed tables of the findings from the included studies will be provided, with reference to the type of study (i.e. randomized, cohort studies, case control studies...), origin (country), the study period (year), the inclusion/exclusion criteria, type of analysed outcomes, and modality of screening adopted. In addition, additional tables will be provided listing relevant characteristics of each study.

5.5.2. Data analysis

All extracted data will be tabulated with Microsoft with Microsoft Excel (Microsoft Corporation, Redmond, WA). Percentage for screened, eligible patients as well subjects affected by TAA and/or TADA will be provided. Percentages of other associated concomitant vascular and cardiac disease will be listed as well concomitant associated clinical features.

6. COMPETING INTERESTS

The authors declare that they have no competing interests.

7. REFERENCES

1. Erbel R, Aboyans V, Boileau C, Bossone E, Bartolomeo RD, Eggebrecht H, Evangelista A, Falk V, Frank H, Gaemperli O, Grabenwöger M, Haverich A, Iung B, Manolis AJ, Meijboom F, Nienaber CA, Roffi M, Rousseau H, Sechtem U, Sirnes PA, Allmen RS, Vrints CJ; ESC Committee for Practice Guidelines . 2014 ESC guidelines on the diagnosis and treatment of aortic diseases: document covering acute and chronic aortic diseases of the thoracic and abdominal aorta of the adult. The Task Force for the Diagnosis and Treatment of Aortic Diseases of the European Society of Cardiology (ESC). *Eur Heart J.* 2014;35:2873–2926.
2. Hiratzka LF, Bakris GL, Beckman JA, Bersin RM, Carr VF, Casey DE Jr., Eagle KA, Hermann LK, Isselbacher EM, Kazerooni EA, Kouchoukos NT, Lytle BW, Milewicz DM, Reich DL, Sen S, Shinn JA, Svensson LG, Williams DM; American College of Cardiology Foundation; American Heart Association Task Force on Practice Guidelines; American Association for Thoracic Surgery; American College of Radiology; American Stroke Association; Society of Cardiovascular Anesthesiologists; Society for Cardiovascular Angiography and Interventions; Society of Interventional Radiology; Society of Thoracic Surgeons; Society for Vascular Medicine . 2010 ACCF/AHA/AATS/ACR/ASA/SCA/SCAI/SIR/STS/SVM Guidelines for the diagnosis and management of patients with thoracic aortic disease. A report of the American College of Cardiology Foundation/American Heart Association Task Force on Practice Guidelines, American Association for

Thoracic Surgery, American College of Radiology, American Stroke Association, Society of Cardiovascular Anesthesiologists, Society for Cardiovascular Angiography and Interventions, Society of Interventional Radiology, Society of Thoracic Surgeons, and Society for Vascular Medicine. *J Am Coll Cardiol.* 2010;55:e27–e129.

3. Von Allmen RS, Anjum A, Powell JT. Incidence of descending aortic pathology and evaluation of the impact of thoracic endovascular aortic repair: a population-based study in England and Wales from 1999 to 2010. *Eur J Vasc Endovasc Surg* 2013;45:154-9.
4. Bottle A, Mariscalco G, Shaw MA, Benedetto U, Saratzis A, Mariani S, Bashir M, Aylin P, Jenkins D, Oo AY, Murphy GJ; UK Aortic Forum. Unwarranted Variation in the Quality of Care for Patients With Diseases of the Thoracic Aorta. *J Am Heart Assoc.* 2017;6(3).
5. Sampson UK, Norman PE, Fowkes FG, Aboyans V, Yanna Song, Harrell FE Jr., Forouzanfar MH, Naghavi M, Denenberg JO, McDermott MM, Criqui MH, Mensah GA, Ezzati M, Murray C. Global and regional burden of aortic dissection and aneurysms: mortality trends in 21 world regions, 1990 to 2010. *Glob Heart.* 2014;9:171–180.
6. Olsson C, Thelin S, Ståhle E, Ekbom A, Granath F. Thoracic Aortic Aneurysm and Dissection. Increasing Prevalence and Improved Outcomes Reported in a Nationwide population-based study of more than 14 000 cases from 1987 to 2002. *Circulation.* 2006;114:2611-8.
7. Russo CF, Mariscalco G, Colli A, Santè P, Nicolini F, Miceli A, De Chiara B, Beghi C, Gerosa G, Glauber M, Gherli T, Nappi G, Murzi M, Molardi A, Merlanti B, Vizzardi E, Bonadei I, Coletti G, Carrozzini M, Gelsomino S, Caiazzo A, Lorusso R. Italian multicentre study on type A acute aortic dissection: a 33-year follow-up. *Eur J Cardiothorac Surg.* 2016;49:125–131.
8. Raghupathy A, Nienaber CA, Harris KM, Myrmel T, Fattori R, Sechtem U, Oh J, Trimarchi S, Cooper JV, Booher A, Eagle K, Isselbacher E, Bossone E; International Registry of Acute Aortic Dissection (IRAD) Investigators . Geographic differences in clinical presentation, treatment, and outcomes in type A acute aortic dissection (from the International Registry of Acute Aortic Dissection). *Am J Cardiol.* 2008;102:1562–1566.
9. Chikwe J, Cavallaro P, Itagaki S, Seigerman M, Diluozzo G, Adams DH. National outcomes in acute aortic dissection: influence of surgeon and institutional volume on operative mortality. *Ann Thorac Surg.* 2013;95:1563–1569.
10. Andersen ND, Ganapathi AM, Hanna JM, Williams JB, Gaca JG, Hughes GC. Outcomes of acute type a dissection repair before and after implementation of a multidisciplinary thoracic aortic surgery program. *J Am Coll Cardiol.* 2014;63:1796–1803.
11. Hughes GC, Zhao Y, Rankin JS, Scarborough JE, O'Brien S, Bavaria JE, Wolfe WG, Gaca JG, Gammie JS, Shahian DM, Smith PK. Effects of institutional volumes on operative outcomes for aortic root replacement in North America. *J Thorac Cardiovasc Surg.* 2013;145:166–170.
12. Liao JM, Bakaeen FG, Cornwell LD, Simpson K, Lemaire SA, Coselli JS, Chu D. Nationwide trends and regional/hospital variations in open versus endovascular repair of thoracoabdominal aortic aneurysms. *J Thorac Cardiovasc Surg.* 2012;144:612–616.
13. Goodney PP, Brooke BS, Wallaert J, Travis L, Lucas FL, Goodman DC, Cronenwett JL, Stone DH. Thoracic endovascular aneurysm repair, race, and volume in thoracic aneurysm repair. *J Vasc Surg.* 2013;57:56–63.
14. Patel ND, Weiss ES, Alejo DE, Nwakanma LU, Williams JA, Dietz HC, Spevak PJ, Gott VL, Vricella LA, Cameron DE. Aortic root operations for Marfan syndrome: a comparison of the Bentall and valve-sparing procedures. *Ann Thorac Surg.* 2008;85:2003-10;discussion 10-1.
15. Kim TS, Na CY, Oh SS, Kim JH. Long-term mortality and morbidity after button Bentall operation. *J Card Surg.* 2013;28:280-4.
16. Svensson LG, Longoria J, Kimmel WA, Nadolny E. Management of aortic valve disease during aortic surgery. *Ann Thorac Surg.* 2000;69:778-83.
17. David TE, Ivanov J, Armstrong S, Feindel CM, Webb GD. Aortic valve-sparing operations in patients with aneurysms of the aortic root or ascending aorta. *Ann Thorac Surg.* 2002;74:S1758-61; discussion S92-9.
18. Hagan PG, Nienaber CA, Isselbacher EM, et al. The International Registry of Acute Aortic Dissection (IRAD): new insights into an old disease. *JAMA.* 2000;283:897-903.
19. Chiappini B, Schepens M, Tan E, Dell' Amore A, Morshuis W, Dossche K, Bergonzini M, Camurri N, Reggiani LB, Marinelli G, Di Bartolomeo R. Early and late outcomes of acute type A aortic dissection: analysis of risk factors in 487 consecutive patients. *Eur Heart J.* 2005;26(2):180-6.

20. Sundt TM, 3rd, Orszulak TA, Cook DJ, Schaff HV. Improving results of open arch replacement. *Ann Thorac Surg.* 2008;86:787-96; discussion -96.

21. Goodney PP, Travis L, Lucas FL, Fillinger MF, Goodman DC, Cronenwett JL, Stone DH. Survival after open versus endovascular thoracic aortic aneurysm repair in an observational study of the Medicare population. *Circulation.* 2011;124(24):2661-9.
22. Coady MA1, Davies RR, Roberts M, Goldstein LJ, Rogalski MJ, Rizzo JA, Hammond GL, Kopf GS, Elefteriades JA. Familial patterns of thoracic aortic aneurysms. *Arch Surg.* 1999;134:361-7.
23. Isselbacher EM, Lino Cardenas CL, Lindsay. Hereditary Influence in Thoracic Aortic Aneurysm and Dissection ME. *Circulation.* 2016;133:2516-28.
24. Biddinger A, Rocklin M, Coselli J, Milewicz DM. Familial thoracic aortic dilatations and dissections: a case control study. *J Vasc Surg.* 1997;25:506-11.
25. Milewicz DM, Chen H, Park ES, Petty EM, Zaghi H, Shashidhar G, Willing M, Patel V. Reduced penetrance and variable expressivity of familial thoracic aortic aneurysms/dissections. *Am J Cardiol.* 1998;82:474-9.
26. Milewicz DM, Regalado E. Heritable Thoracic Aortic Disease Overview. In: Pagon RA, Adam MP, Ardinger HH, Wallace SE, Amemiya A, Bean LJH, Bird TD, Ledbetter N, Mefford HC, Smith RJH, Stephens K, editors. GeneReviews® [Internet]. Seattle (WA): University of Washington, Seattle; 1993-2017.
27. Evangelista A. Imaging aortic aneurysmal disease. *Heart.* 2014;100:909-15.
28. Regalado ES, Guo DC, Prakash S, Bensend TA, Flynn K, Estrera A, Safi H, Liang D, Hyland J, Child A, Arno G, Boileau C, Jondeau G, Braverman A, Moran R, Morisaki T, Morisaki H; Montalcino Aortic Consortium., Pyeritz R, Coselli J, LeMaire S, Milewicz DM. Aortic Disease Presentation and Outcome Associated With ACTA2 Mutations. *Circ Cardiovasc Genet.* 2015;8:457-64.
29. Williams JA, Loeys BL, Nwakanma LU, Dietz HC, Spevak PJ, Patel ND, François K, DeBacker J, Gott VL, Vricella LA, Cameron DE. Early surgical experience with LoeysDietz: a new syndrome of aggressive thoracic aortic aneurysm disease. *Ann Thorac Surg.* 2007;83:S757–63.
30. Higgins JPT, Gree S (editors), Cochrane Handbook of Systemic Reviews of Inteventions. Chichester (UK): John Wiley & Sons, 2011.
31. Google translate. http://translate.google.com. Accessed April 30, 2015.
32. Balk EM, Chung M, Chen ML, Chang LK, Trikalinos TA. Data extraction from machine-translated versus original language randomized trial reports: a comparative study. *Syst Rev.* 2013;2:97.
33. Wells GA, Shea B, O'Connell D, Peterson J, Welch V, Losos M, Tugwell P. The Newcastle-Ottawa Scale (NOS) for assessing the quality of nonrandomised studies in meta-analyses. Ottawa Hospital Research Institute. http://www.ohri.ca/programs/clinical_epidemiology/oxford.asp. Accessed June 30, 2017.
34. Harris RP, Helfand M, Woolf SH, Lohr KN, Mulrow CD, Teutsch SM, Atkins D; Methods Work Group, Third US Preventive Services Task Force. Current methods of the US Preventive Services Task Force: a review of the process. *Am J Prev Med*. 2001;20(3 Suppl):21-35.
35. Higgins JPT, Green S (editors). Cochrane Handbook for systematic reviews of interventions. Chichester, UK: John Wiley and Sons; 2008.

SUPPLEMENTAL METHODS

Literature search strategy

Our keywords and MeSH terms pertinent to the exposure of interest were used in relevant combinations and they are showed below.

**PubMed**	
**Website**	https://www.ncbi.nlm.nih.gov/pubmed
**Access**	December, 31 2017
**Filters**	none
**Fields**	Title, Abstract
**Search terms**	“aorta, thoracic”<br>“aortic aneurysm“<br>“aortic dissection“<br>“aneurysm“<br>“dissecting“<br>“familial aortic aneurysm”<br>“sporadic thoracic aorta”<br>“screening”<br>“screening aortic aneurysm”<br>“screening<br>“first-degree relatives”<br>“relatives”<br>“siblings”<br>“pedigree”<br>"echocardiography"<br>"computed tomography"<br>"magnetic resonance"<br>“gene”<br>“genetic”<br>“linkage analysis”<br>“next-generation sequencing”<br>“mutation”<br>“whole exome”<br>“exome sequencing”<br>“exome sequence”<br>“targeted array”<br>“genome-wide association study”<br>“whole genome sequencing”<br>“whole genome sequence”
Number of articles	**10364** (10094 + 270)

Search 10094

("aorta, thoracic" OR "aortic aneurysm" OR "aortic dissection" OR "aneurysm" OR "dissecting") AND ("screening" OR “screening aortic aneurysm”) AND ("echocardiography" OR "computed tomography" OR "magnetic resonance" OR “next-generation sequencing” OR “next-generation sequence” OR “genetic” OR “genes” OR “gene” OR “mutation” OR “whole-exome” OR “whole exome” OR “exome sequencing” OR “exome sequence” OR “targeted array” OR “genome-wide association study” OR “whole genome sequencing” OR “whole genome sequence” OR “linkage analysis”)

Search 270

("aorta, thoracic" OR "aortic aneurysm" OR "aortic dissection" OR "aneurysm" OR "dissecting") AND ("screening" OR “screening aortic aneurysm”) AND ("relatives" OR "siblings" OR "pedigree" OR "first degree relatives")

EMBASE

**Website**	https://hdas.nice.org.uk/
**Access**	December, 31 2017
**Filters**	none
**Fields**	Title, Abstract
**Search terms**	“thor*”
	“aortic aneurysm“
	“aortic dissection“
	“aneurysm“
	“dissecting“
	“familial aortic aneurysm”
	“sporadic thoracic aorta”
	“screening”
	“screening aortic aneurysm”
	“screening
	“first-degree relatives”
	“relatives”
	“siblings”
	“pedigree”
	"echocardiography"
	"computed tomography"
	"magnetic resonance"
	“gene”
	“genetic”
	“linkage analysis”
	“next-generation sequencing”
	“mutation”
	“whole exome”
	“exome sequencing”
	“exome sequence”
	“targeted array”
	“genome-wide association study”
	“whole genome sequencing”
	“whole genome sequence”

Search **914**

(((("aorta" AND "thor*") OR "aortic aneurysm" OR "aortic dissection" OR "aneurysm" OR "dissecting") AND ("screening" OR "screening aortic aneurysm")) AND ("echocardiography" OR "computed tomography" OR "magnetic resonance" OR "next-generation sequencing" OR "next-generation sequence" OR "genetic" OR "genes" OR "gene" OR "mutation" OR "whole-exome" OR "whole exome" OR "exome sequencing" OR "exome sequence" OR "targeted array" OR "genome-wide association study" OR "whole genome sequencing" OR "whole genome sequence" OR "linkage analysis")).ti,ab

Cochrane Library

**Website**	http://onlinelibrary.wiley.com/cochranelibrary/search
**Access**	December, 31 2017
**Filters**	none
**Search option**	Search Manager
**Search terms**	“thoracic aorta”<br>“thoracic aortic aneurysm”<br>“thoracic aortic dissection”<br>"familial aortic dissection"<br>“screening “<br>“first-degree relatives”<br>“siblings”<br>“pedigree”<br>“echocardiography”<br>“computed tomography”<br>“magnetic resonance”<br>“gene”<br>“genetic”<br>“linkage analysis”<br>“mutation”<br>“exome sequencing”<br>“exome sequence”<br>“genome-wide association scan”<br>"genome wide linkage scan"<br>“whole genome sequencing”<br>“whole genome sequence”

Number of articles **165** (13 + 124 + 24 + 4)

Search 13
("thoracic aorta" OR "thoracic aortic aneurysm" OR "thoracic aortic dissection" OR “familial aortic dissection”) AND ("screening") AND ("first degree relatives" OR "family" OR “pedigree” OR "echocardiography" OR "computed tomography" OR "magnetic resonance" OR "gene" OR “genetic” OR “linkage analysis” OR “mutation” OR “exome sequencing” OR “exome sequence” OR “genome-wide association scan” OR "genome wide linkage scan" OR “whole genome sequencing” OR “whole genome sequence”)

Search 124
("thoracic aorta" OR "thoracic aortic aneurysm" OR "thoracic aortic dissection" OR “familial aortic dissection”) AND ("echocardiography" OR "computed tomography" OR "magnetic resonance" OR "gene" OR “genetic” OR “linkage analysis” OR “mutation” OR “exome sequencing” OR “exome sequence” OR “genome-wide association scan” OR "genome wide linkage scan" OR “whole genome sequencing” OR “whole genome sequence”)

Search 24
("thoracic aorta" OR "thoracic aortic aneurysm" OR "thoracic aortic dissection" OR “familial aortic dissection”) AND ("screening")

Search 4
("thoracic aorta" OR "thoracic aortic aneurysm" OR "thoracic aortic dissection" OR “familial aortic dissection”) AND ("screening") AND ("first degree relatives" OR "family" OR “pedigree”)

OMIM

**Website**	https://www.omim.org		
**Access**	December, 31 2017		
**Filters**	Title		
Entries	2454	for	“thoracic aneurysm-associated genes”
Entries	582	for	“aortic aneurysm, familial thoracic”
Entries	59	for	“thoracic aneurysm/dissection”
Entries, total	3095		
Papers identified	**106**		

Citations identified through “first-generation” reference list

Study (Author/Year)	Ref.N.
Barbier et al. 2014[1]	38
Bee et al. 2012[2]	26
Chamney et al. 2015[3]	9
Disabella et al. 2011[4]	19
Disertori et al. 1991[5]	21
Dong et al. 2014[6]	12
Francke et al. 1995[7]	33
Gago-Diaz et al. 2014[8]	24
Gago-Diaz et al. 2016[9]	13
Guo et al. 2001[10]	28
Guo et al. 2007[11]	30
Guo et al. 2009[12]	33
Guo et al. 2011[13]	20
Guo et al. 2013[14]	21
Guo et al. 2015[15]	40
Guo et al. 2016[16]	27
Hannuksela et al. 2015[17]	14
Hannuksela et al. 2016[18]	29
Harakalova et al. 2013[19]	23
Hasham et al. 2003[20]	26
Kakko et al. 2003[21]	20
Kent et al. 2013[22]	23
Keramati et al. 2010[23]	22
Khau Van Kien et al. 2004[24]	37
Khau Van Kien et al. 2005[25]	33
Kuang et al. 2016[26]	40
Loscalzo et al. 2007[27]	46
Marwick et al. 1987[28]	7
McManus et al. 1987[29]	45
Milewicz et al. 1998[30]	16
Morisaki et al. 2009[31]	21
Pannu et al. 2005[32]	31
Pannu et al. 2007[33]	38
Regalado et al. 2011[34]	23
Regalado et al. 2011[35]	23

Regalado et al. 2011[36]	27
Renard et al. 2013[37]	35
Robertson et al. 2016[38]	36
Sherrah et al. 2016[39]	30
Takeda et al. 2015[40]	9
Teixidó-Turà et al. 2014[41]	6
Tortora et al.[42]	24
Tran-Fadulo et al. 2006[43]	21
Tran-Fadulo et al. 2009[44]	21
Vaughan et al. 2001[45]	35
Wang et al. 2010[46]	29
Wang et al. 2013[47]	35
Ware et al. 2014[48]	19
Warnes et al. 1985[49]	12
Weigang et al. 2007[50]	30
Yoo et al. 2010[51]	15
Zhu et al. 2006[52]	30
Ziganshin et al. 2015[53]	23
Total	**1348**

Table S1. PRISMA checklist of items to include when Reporting a Systematic Review or Meta-analysis*

Section/topic	#	Checklist Item	Reported on Page #
**TITLE**			
Title	1	Identify the report as a systematic review, meta-analysis, or both.	1
**ABSTRACT**			
Structured summary	2	Provide a structured summary including, as applicable: background; objectives; data sources; study eligibility criteria, participants, and interventions; study appraisal and synthesis methods; results; limitations; conclusions and implications of key findings; systematic review registration number.	2
**INTRODUCTION**			
Rationale	3	Describe the rationale for the review in the context of what is already known.	3
Objectives	4	Provide an explicit statement of questions being addressed with reference to participants, interventions, comparisons, outcomes, and study design (PICOS).	3
**METHODS**			
Protocol and registration	5	Indicate if a review protocol exists, if and where it can be accessed (e.g., Web address), and, if available, provide registration information including registration number.	4 (Data S1)
Eligibility criteria	6	Specify study characteristics (e.g., PICOS, length of follow-up) and report characteristics (e.g., years considered, language, publication status) used as criteria for eligibility, giving rationale.	4 (Data S1)
Information sources	7	Describe all information sources (e.g., databases with dates of coverage, contact with study authors to identify additional studies) in the search and date last searched.	4 (Data S1)
Search	8	Present full electronic search strategy for at least one database, including any limits used, such that it could be repeated.	4
Study selection	9	State the process for selecting studies (i.e., screening, eligibility, included in systematic review, and, if applicable, included in the meta-analysis).	4,5 (Data S1)
Data collection process	10	Describe method of data extraction from reports (e.g., piloted forms, independently, in duplicate) and any processes for obtaining and confirming data from investigators.	4,5 (Data S1)
Data items	11	List and define all variables for which data were sought (e.g., PICOS, funding sources) and any assumptions and simplifications made.	4,5 (Table S2)
Risk of bias in individual studies	12	Describe methods used for assessing risk of bias of individual studies (including specification of whether this was done at the study or outcome level), and how this information is to be used in any data synthesis.	6

			(Table S9)
Summary measures	13	State the principal summary measures (e.g., risk ratio, difference in means).	5
Synthesis of results	14	Describe the methods of handling data and combining results of studies, if done, including measures of consistency (e.g., I^2) for each meta-analysis.	6
Risk of bias across studies	15	Specify any assessment of risk of bias that may affect the cumulative evidence (e.g., publication bias, selective reporting within studies).	6
Additional analyses	16	Describe methods of additional analyses (e.g., sensitivity or subgroup analyses, meta-regression), if done, indicating which were pre-specified.	6
**RESULTS**			
Study selection	17	Give numbers of studies screened, assessed for eligibility, and included in the review, with reasons for exclusions at each stage, ideally with a flow diagram.	7
Study characteristics	18	For each study, present characteristics for which data were extracted and provide the citations.	7
Risk of bias within studies	19	Present data on risk of bias of each study and, if available, any outcome level assessment (see item 12).	7
Results of individual studies	20	For all outcomes considered (benefits or harms), present, for each study: (a) simple summary data for each intervention group (b) effect estimates and confidence intervals, ideally with a forest plot.	7-10 (Supplement)
Synthesis of results	21	Present results of each meta-analysis done, including confidence intervals and measures of consistency.	7-10
Risk of bias across studies	22	Present results of any assessment of risk of bias across studies (see Item 15).	7-10
Additional analysis	23	Give results of additional analyses, if done (e.g., sensitivity or subgroup analyses, meta-regression [see Item 16]).	7-10
**DISCUSSION**			
Summary of evidence	24	Summarize the main findings including the strength of evidence for each main outcome; consider their relevance to key groups (e.g., healthcare providers, users, and policy makers).	11
Limitations	25	Discuss limitations at study and outcome level (e.g., risk of bias), and at review-level (e.g., incomplete retrieval of identified research, reporting bias).	11,12
Conclusions	26	Provide a general interpretation of the results in the context of other evidence, and implications for future research.	14
**FUNDING**			
Funding	27	Describe sources of funding for the systematic review and other support (e.g., supply of data); role of funders for the systematic review.	14

*From: Moher D, Liberati A, Tetzlaff J, Altman DG; PRISMA Group. Preferred reporting items for systematic reviews and meta-analyses: the PRISMA statement. BMJ 2009;339:b2535.

Table S2. PICOS criteria for inclusion and exclusion of studies into meta-analysis

Parameter	Inclusion criteria	Exclusion criteria
**Patients**	Patients affected by NS-TAD	Patients affected by syndromic TAD and other cardiac diseases other than TAD
**Intervention**	Screening using the genetic and/or imaging modalities, including transthoracic echocardiography, computed tomography, and magnetic resonance	-
**Comparator**	The screening interventions listed above versus each other or versus no intervention	-
**Outcomes**	Primary: new diagnosis of TAD (aortic aneurysm and dissection) in first-, second-, and third-degree relatives<br>Secondary: effectives of screening modality (eligible vs screened relatives), disease-specific mortality, disease specific genetic mutation, cost-effectiveness, age and aortic diamters at dissection/grow rate	-
**Study design**	Clinical randomised trials<br>Controlled before-and-after studies<br>Prospective and retrospective cohort studies<br>Cross-sectional studies<br>Case-control studies	Repeat publications of the same analysis or dataset<br>Conference abstracts<br>Editorials & opinion pieces<br>Books or grey literature

NS indicates non syndromic; TAD, thoracic aortic disease.

Table S3. Full details of the screened family relatives with number and ID of the included families

Study (Author/Year)	Country	NS-TAD Form	Ethnicity	Exclusion criteria	N. Families screened	N. Relatives (pedigree)	Name (ID) of families screened*
Barbier et al. 2014[1]	France	FTAAD	White European (French)	MFS and other syndromic forms of TAAD	2	40	TAA-9801, TAA-9178
Bee et al. 2012[2]	USA	FTAA	White American (80%)	MFS, LDS, and EDS	9	54	ANS, JNW, SY92, JNE, ANHH, ANO, KNA, ANV, KNK
Chamney et al. 2015[3]	United Kingdom	FTAAD	White European (North Irish)	Ns	1	14	ns
Disabella et al. 2011[4]	Italy	FTAAD	ns	MFS, LDS, EDS, BAV, and known mutations in: FBN1, TGFBR1, TGFBR2, NOTCH1, COL3A1	5	37	ns
Disertori et al. 1991[5]	Italy	FTAAD	White European (Italian)	ns	1	30	ns
Dong et al. 2014[6]	China	FTAAD	Chinese (Han)	ns	1	64	ns
Francke et al. 1995[7]	USA	FTAAD	Americans of European descent	ns	1	26	ns
Gago-Diaz et al. 2014[8]	Spain	FTAAD	White European (Spanish)	ns	1	31	ns
Gago-Diaz et al. 2016[9]	Spain	FTAAD	White European (Spanish)	ns	1	30	ns
Guo et al. 2001[10]	USA†	FTAA	Caucasian (13 pedigrees), Iranian (one pedigree), Japanese (one pedigree)	Excluded linkage to FBN1	15	219	**TAA001, TAA002, TAA003, TAA005**, TAA009, TAA010, TAA011, TAA012, TAA013, TAA014, TAA015, TAA025, TAA030, TAA033, TAA034
Guo et al. 2007[11]	USA†	FTAAD	ns	ns	14	212	TAA015, **TAA020, TAA039, TAA041, TAA105, TAA133**, TAA166, **TAA174, TAA313, TAA327, TAA349, TAA370, TAA377, TAA390**

Table S3 (Continued)							
**Study (Author/Year)**	**Country**	**NS-TAD Form**	**Ethnicity**	**Exclusion criteria**	**N. Families screened**	**N. Relatives (pedigree)**	**Name (ID) of families screened***
Guo et al. 2009[12]	USA†	FTAAD	ns	Known genetic syndrome	20	269	**TAA020, TAA039, TAA041, TAA105, TAA133, TAA174**, TAA252, **TAA313, TAA327, TAA331, TAA349, TAA370, TAA377, TAA390**, TAA441, TAA455, p.R212Q, p.R212Q, p.R258C, pT326N
Guo et al. 2011[13]	USA†	FTAAD/pAA	Americans of (Northern) European descent	ns	1	28	TAA254
Guo et al. 2013[14]	USA†	FTAAD	ns	Mutations in already known genes associated with FTAAD	6	89	TAA165, **TAA216**, TAA292, TAA508, TAA561, TAA690
Guo et al. 2015[15]	USA†	BAV/TAA	ns	ns	1	48	TAA059
Guo et al. 2016[16]	USA†	FTAAD	European-American	ns	6	65	TAA111, TAA271, TAA602, TAA703, TAA-9544, TAA-92291,
Hannuksela et al. 2015[17]	Sweden	FTAAD	White European (Swedish)	ns	7	266	FTAAD1, FTAAD2, FTAAD3, FTAAD4, FTAAD5, FTAAD6, FTAAD7
Hannuksela et al. 2016[18]	Sweden	FTAAD	White European (Swedish)	ns	1	46	ns
Harakalova et al. 2013[19]	Holland	TAAD/PDA	White European (Dutch)	ns	2	75	TAAD01-TAAD02
Hasham et al. 2003[20]	USA†	FTAAD	White European (Swiss-German)	ns	1	69	**TAA035**
Kakko et al. 2003[21]	Finland	FTAAD	White European (Finnish)	Family with <2 TAD affected pts	11	213	1,2,3,4,5,6,7,8,9,10,11
Kent et al. 2013[22]	USA	BAV/TAA	ns	Dysmorphic/connective tissue manifestations	14	129	**A**,**D**,F,**G**,H,**I**,**J**,**K**,**L**,**M**,**Q**,**R**,S,T
Keramati et al. 2010[23]	USA	FTAAD	Iranian	ns	1	23	ns
Khau Van Kien et al. 2004[24]	France	FTAAD/PDA	White European (French)	ns	1	68	**Bourgogne family**

**Table S3 (Continued)**							
**Study (Author/Year)**	**Country**	**NS-TAD Form**	**Ethnicity**	**Exclusion criteria**	**N. Families screened**	**N. Relatives (pedigree)**	**Name (ID) of families screened***
Khau Van Kien et al. 2005[25]	France	FTAAD/PDA	White European (French)	ns	1	87	**Bourgogne family**
Kuang et al. 2016[26]	USA†	FTAAD	White European	Family with <2 TAD affected pts	2	40	TAA337-MS300
Loscalzo et al. 2007[27]	USA	BAV/TAA	ns	ns	13	194	**A,D,G,I,J,K,L,M,N,O,P,Q,R**
Marwick et al. 1987[28]	Australia	FTADiss	Australian	ns	1	17	ns
McManus et al. 1987[28]	USA	FTADiss	White American	ns	1	19	ns
Milewicz et al. 1998[30]	USA†	FTAAD	ns	MFS	6	123	**TAA001, TAA002, TAA003**, TAA004, **TAA005**, TAA006
Morisaki et al. 2009[31]	Japan	FTAAD	Japanese	ns	3	47	1,2,3
Pannu et al. 2005[32]	USA†	FTAAD	White European (Swiss-German)	MFS	4	235	**TAA035**, TAA067,TAA090, TAA150
Pannu et al. 2007[33]	USA†	FTAAD	ns	ns	2[‡]	27	TAA027, TAA069
Regalado et al. 2011[34]	USA†	FTAAD/ICA	ns	Family with < 2 TAD affected pts; MFS, and LDS	13[§]	231	TAA008, TAA059, TAA062, TAA113, TAA175, **TAA258**, TAA287, TAA288, TAA311, TAA395, TAA467, TAA480, **TAA549**
Regalado et al. 2011[35]	USA†	FTAAD/ICA/pAA	ns	Family with <2 TAD affected pts	5	106	TAA071, TA0072, TAA115, TAA365, **TAA549**
Regalado et al. 2011[36]	USA†	FTAAD	ns	Family with <2 TAD affected pts; MFS, and LDS	5[‖]	29	**TAA258**, TAA321, TAA345, TAA394, TAA748
Renard et al. 2013[37]	Belgium	FTAAD	ns	MFS	8	97	1,2,3,4,5,6,7,8
Robertson et al. 2016[38]	Australia	FTAAD	ns	Syndromic TAD, BAV, vasculitis	270	1267	ns
Sherrah et al. 20016[39]	Australia	FTAAD	ns	Patients < 16 or > 60 yrs	ns	ns	ns
Takeda et al. 2015[40]	Japan	FTAAD	Japanese	ns	1	17	ns
Tortora et al. 2017[41]	Italy	BAV/TAA	Ns	Ns	20	97	ns
Teixidó-Turà et al. 2014[42]	Spain	FTAAD	White European (Spanish)	ns	1	36	ns
Tran-Fadulo et al. 2006[43]	USA†	FTAAD	ns	ns	3	153	TAA105, TAA174, **TAA216**

Table S3 (Continued)							
**Study (Author/Year)**	**Country**	**NS-TAD Form**	**Ethnicity**	**Exclusion criteria**	**N. Families screened**	**N. Relatives (pedigree)**	**Name (ID) of families screened***
Tran-Fadulo et al. 2009[44]	USA†	FTAAD	ns	Family with <2 TAD affected pts; MFS, and LDS	4	78	TAA009, TAA023, TAA336, TAA339
Vaughan et al. 2001[45]	USA†	FTAA	Northern European	MFS and EDS	3	67	ANA, ANB, ANF
Wang et al. 2010[46]	USA†	FTADiss	ns	Family with <2 TAD affected pts	2	48	TAA026, TAA400
Wang et al. 2013[47]	China	FTAAD	Chinese (Han)	(MFS included)	1[#]	10	Family 4
Ware et al. 2014[48]	USA	FTAAD	White American	ns	1	7	ns
Warnes et al. 1985[49]	USA	FTAAD	White American	ns	1	6	ns
Weigang et al. 2007[50]	Germany	FTAAD	ns	Syndromic TAD	ns	26	ns
Yoo et al. 2010[51]	Korea	FTAAD	Korean	ns	1	20	ns
Zhu et al. 2006[52]	France	FTAAD/PDA	French and American	ns	2	49	"French" and "American" families
Ziganshin et al. 2015[53,**]	USA	FTAAD	ns	ns	1	27	ns
Ziganshin et al. 2015[53,**]	USA	FTAAD	ns	ns	1	17	ns

BAV indicates bicuspid aortic valve; EDS, Ehlers-Danlos syndrome; FTAA, familial thoracic aortic aneurysm; FTAAD, familial thoracic aortic aneurysm and dissection; FTAD, familial thoracic aortic dissection; ICA, intracranial aneurysm; LSD, Loeys-Dietz syndrome; MFS, Marfan syndrome; ns, not specified; ns, not specified; pAA, peripheral artery aneurysm; PDA, patent ductus arteriosus; TAD, thoracic aortic disease.

*Families analysed multiple studies are underlined in similar colours. †Study performed at University of Texas (USA) only. [‡]96 families considered in total, but data available for 2 (pedigree) families only. [§]48 families considered in total, but data available for 13 (pedigree) families only. [‖]183 families considered in total, but data available for 5 (pedigree) families only. [#]Other 6 families with Marfan syndrome considered, but excluded from the analysis (inclusion criteria as per protocol). [**]Data of two different screened families obtained from the same study (53).

Table S4. Full details of the family pedigree, eligible, screened, and affected patients and relatives

Study (Author/Year)	NS-TAD Form	Family N.	Probands N.	Total. N. subjects from pedigree	Subjects eligible for screening		Subjects screened		Subjects affected (aneurysm+dissection)		Newly diagnosed affected relatives (aneurysm+dissection)	
					N.	%	N.	%	N.	%	N.	%
Barbier et al. 2014[1]	FTAAD	2	2	40	35	88	13	33	9	23	7	18
Bee et al. 2012[2]	FTAA	9	9	54	44	81	32	59	21	39	12	22
Chamney et al. 2015[3]	FTAAD	1	1	14	11	79	6	43	6	43	5	36
Disabella et al. 2011[4]	FTAAD	5	5	37	22	59	29	78	15	41	10	27
Disertori et al. 1991[5]	FTAAD	1	2	30	24	80	14	47	4	13	2	7
Dong et al. 2014[6]	FTAAD	1	1	64	53	83	39	61	9	14	8	13
Francke et al. 1995[7]	FTAAD	1	1	26	22	85	23	88	10	38	9	35
Gago-Diaz et al. 2014[8]	FTAAD	1	1	31	22	71	12	39	7	23	6	19
Gago-Diaz et al. 2016[9]	FTAAD	1	1	30	25	83	14	47	11	37	10	33
Guo et al. 2001[10]	FTAAD	15	n/a	219	141	64	121	55	73	33	n/c	n/c
Guo et al. 2007[11]	FTAAD	14	n/a	212	151	71	130	61	53	25	n/c	n/c
Guo et al. 2009[12]	FTAAD	20	n/a	269	176	65	163	61	66	25	n/c	n/c
Guo et al. 2011[13]	FTAAD/pAA	1	1	28	22	79	18	64	9	32	8	29
Guo et al. 2013[14]	FTAAD	6	6	89	49	55	39	44	37	42	31	35
Guo et al. 2015[15]	BAV/TAA	1	1	48	35	73	34	71	8	17	7	15
Guo et al. 2016[16]	FTAAD	6	6	65	38	58	21	32	21	32	15	23
Hannuksela et al. 2015[17]	FTAAD	7	7	270	135	50	106	40	44	16	37	14
Hannuksela et al. 2016[18]	FTAAD	1	1	46	31	67	19	41	6	13	n/c	n/c
Harakalova et al. 2013[19]	TAAD/PDA	2	2	75	47	63	40	53	15	20	13	17
Hasham et al. 2003[20]	FTAAD	1	1	69	61	88	52	75	17	25	16	23
Kakko et al. 2003[21]	FTAAD	11	n/a	213	150	70	115	54	39	18	n/c	n/c
Kent et al. 2013[22]	BAV/TAA	14	14	129	94	73	93	72	48	37	34	26

Table S4 (Continued)												
Study (Author/Year)	NS-TAD Form	Family N.	Probands N.	Total. N. subjects from pedigree	Subjects eligible for screening		Subjects screened		Subjects affected (aneurysm+dissection)		Newly diagnosed affected relatives (aneurysm+dissection)	
					N.	%	N.	%	N.	%	N.	%
Keramati et al. 2010[23]	FTAAD	1	1	23	20	87	15	65	13	57	12	52
Khau Van Kien et al. 2004[24]	FTAAD/PDA	1	1	68	50	74	49	72	8	12	7	10
Khau Van Kien et al. 2005[25]	FTAAD/PDA	1	1	87	73	84	78	90	8	9	7	8
Kuang et al. 2016[26]	FTAAD	2	n/a	40	28	70	16	40	11	28	n/c	n/c
Loscalzo et al. 2007[27]	BAV/TAA	13	13	194	137	71	138	71	57	29	44	23
Marwick et al. 1987[28]	FTADiss	1	1	17	15	88	4	24	2	12	1	6
McManus et al. 1987[29]	FTADiss	1	1	19	11	58	8	42	6	32	5	26
Milewicz et al. 1998[30]	FTAAD	6	6	123	89	72	n/a	n/a	30	24	24	20
Morisaki et al. 2009[31]	FTAAD	3	3	47	30	64	9	19	14	30	11	23
Pannu et al. 2005[32]	FTAAD	4	4	235	179	76	72	31	58	25	54	23
Pannu et al. 2007[33]	FTAAD	2	2	27	24	89	23	85	6	22	4	15
Regalado et al. 2011[34]	FTAAD/ICA	13	13*	231	126	55	12	5	52	23	43	19
Regalado et al. 2011[35]	FTAAD/ICA/pAA	5	n/a	106	71	67	36	34	23	22	n/c	n/c
Regalado et al. 2011[36]	FTAAD	5	5	29	16	55	11	38	15	52	10	34
Renard et al. 2013[37]	FTAAD	8	8	97	67	69	29	30	29	30	21	22
Robertson et al. 2016[38]	FTAAD	270	270	nc	n/c	n/c	n/c	n/c	611	n/a	n/c	n/c
Sherrah et al. 2016[39]	FTAAD	539	n/a	nc	n/c	n/c	n/c	n/c	658	n/a	n/c	n/c
Takeda et al. 2015[40]	FTAAD	1	1	17	12	71	9	53	5	29	4	24
Teixidó-Turà et al. 2014[41]	FTAAD	1	1	36	25	69	10	28	3	8	2	6
Tortora et al. 2017[42]												
Tran-Fadulo et al. 2006[43]	FTAAD	3	3	153	106	69	9	6	21	14	18	12
Tran-Fadulo et al. 2009[44]	FTAAD	4	4†	78	62	79	49	63	29	37	26	33
Vaughan et al. 2001[45]	FTAA	3	3	67	61	91	63	94	30	45	27	40

Table S4 (Continued)												
**Study (Author/Year)**	**NS-TAD Form**	**Family N.**	**Probands N.**	**Total. N. subjects from pedigree**	**Subjects eligible for screening**		**Subjects screened**		**Subjects affected (aneurysm+dissection)**		**Newly diagnosed affected relatives (aneurysm+dissection)**	
					**N.**	**%**	**N.**	**%**	**N.**	**%**	**N.**	**%**
Wang et al. 2010[46]	FTADiss	2	n/a	48	34	71	21	44	10	21	n/c	n/c
Wang et al. 2013[47]	FTAAD	1	1	10	7	70	8	80	2	20	1	10
Ware et al. 2014[48]	FTAAD	1	2	7	5	71	7	100	2	29	0	0
Warnes et al. 1985[49]	FTAAD	1	2	6	4	67	2	33	2	33	0	0
Weigang et al. 2007[50]	FTAAD	1	n/a	26	23	88	23	88	9	35	n/c	n/c
Yoo et al. 2010[51]	FTAAD	1	1	20	18	90	6	30	5	25	4	20
Zhu et al. 2006[52]	FTAAD/PDA	2	n/a	49	49	100	49	100	8	16	n/c	n/c
Ziganshin et al. 2015[53,‡]	FTAAD	1	1	27	24	89	15	56	4	15	3	11
Ziganshin et al. 2015[53,‡]	FTAAD	1	1	17	8	47	15	59	7	41	6	35

BAV indicates bicuspid aortic valve; FTAA, familial thoracic aortic aneurysm; FTAAD, familial thoracic aortic aneurysm and dissection; FTAD, familial thoracic aortic dissection; ICA, intracranial aneurysm; n/a, not available; n/c, not computable; pAA, peripheral artery aneurysm; PDA, patent ductus arteriosus.

*4 probands not affected by aortic diseases (aortic aneurysm and/or dissections). [†]1 proband not affected by aortic disease (aortic aneurysm and/or dissection). [‡]Data of two different screened families obtained from the same study (53).

Table S5. Full details of the first, second and third degree realatives of evelauated probands

Study (Author/Year)	Newly diagnosed affected relatives (aneurysm+dissection)		FIRST DEGREE RELATIVES			SECOND DEGREE RELATIVES			THIRD DEGREE RELATIVES			Spouse	
	N.	%.	N.	Affected N.	Not Screened*	N.	Affected N.	Not Screened	N.	Affected N.	Not Screened	N.	Screened
Barbier et al. 2014[1]	7	18	14	6	3	14	1	0	0	0	0	10	assessed
Bee et al. 2012[2]	12	22	37	11	9	3	1	0	0	0	0	5	assessed
Chamney et al. 2015[3]	5	36	8	2	2	3	3	0	0	0	0	2	assessed
Disabella et al. 2011[4]	10	27	23	8	4	5	2	2	4	0	0	0	not assessed
Disertori et al. 1991[5]	2	7	13	2	3	15	0	11	0	0	0	0	not assessed
Dong et al. 2014[6]	8	13	5	1	0	9	1	4	30	6	0	19	not sepcified
Francke et al. 1995[7]	9	35	15	8	2	9	1	4	0	0	0	1	not assessed
Gago-Diaz et al. 2014[8]	6	19	3	2	0	10	4	1	13	0	8	4	assessed
Gago-Diaz et al. 2016[9]	10	33	12	6	1	14	4	5	3	0	1	0	not assessed
Guo et al. 2001[10]	n/a	n/a	n/c	n/c	n/c	n/c	n/c	n/c	n/c	n/c	n/c	n/c	assessed
Guo et al. 2007[11]	n/a	n/a	n/c	n/c	n/c	n/c	n/c	n/c	n/c	n/c	n/c	n/c	assessed
Guo et al. 2009[12]	n/a	n/a	n/c	n/c	n/c	n/c	n/c	n/c	n/c	n/c	n/c	n/c	assessed
Guo et al. 2011[13]	8	29	7	2	1	9	4	2	6	2	0	5	assessed
Guo et al. 2013[14]	31	35	40	19	1	18	6	4	12	6	0	13	assessed
Guo et al. 2015[15]	7	15	10	2	1	14	1	8	15	4	0	8	assessed
Guo et al. 2016[16]	15	23	21	3	2	22	6	11	13	6	2	3	assessed
Hannuksela et al. 2015[17]	37	14	60	17	8	89	11	15	55	9	27	59	not assessed
Hannuksela et al. 2016[18]	n/a	n/a	n/c	n/c	n/c	n/c	n/c	n/c	n/c	n/c	n/c	270	not assessed
Harakalova et al. 2013[19]	13	17	6	2	0	15	2	4	34	9	19	18	assessed
Hasham et al. 2003[20]	16	23	4	3	0	5	2	1	39	11	3	20	assessed
Kakko et al. 2003[21]	n/a	n/a	n/c	n/c	n/c	n/c	n/c	n/c	n/c	n/c	n/c	n/c	assessed
Kent et al. 2013[22]	34	26	73	24	17	21	4	12	19	6	8	2	assessed

Table S5 (Continued)													
**Study (Author/Year)**	**Newly diagnosed affected relatives (aneurysm+dissection)**		**FIRST DEGREE RELATIVES**			**SECOND DEGREE RELATIVES**			**THIRD DEGREE RELATIVES**			**Spouse**	
	**N.**	**%.**	**N.**	**Affected N.**	**Not Screened***	**N.**	**Affected N.**	**Not Screened**	**N.**	**Affected N.**	**Not Screened**	**N.**	**Screened**
Keramati et al. 2010[23]	12	52	10	5	2	8	7	1	0	0	0	4	not assessed
Khau Van Kien et al. 2004[24]	7	10	13	4	2	21	1	2	24	2	7	9	assessed
Khau Van Kien et al. 2005[25]	7	8	13	4	2	26	1	2	38	2	7	9	assessed
Kuang et al. 2016[26]	n/c	n/c	n/c	n/c	n/c	n/c	n/c	n/c	n/c	n/c	n/c	n/c	not assessed
Loscalzo et al. 2007[27]	44	23	72	26	7	37	10	10	65	8	27	7	assessed
Marwick et al. 1987[28]	1	6	7	1	2	5	0	0	0	0	0	4	not assessed
McManus et al. 1987[29]	5	26	7	2	1	9	3	1	0	0	0	2	not assessed
Milewicz et al. 1998[30]	24	20	44	15	9	44	8	7	7	1	0	22	not assessed
Morisaki et al. 2009[31]	11	23	10	2	1	6	3	2	27	6	7	1	not assessed
Pannu et al. 2005[32]	54	23	18	9	1	35	12	2	121	33	19	57	not assessed
Pannu et al. 2007[33]	4	15	16	3	2	4	1	0	0	0	0	5	assessed
Regalado et al. 2011[34]	43	19	83	22	19	64	8	33	50	13	5	21	not assessed
Regalado et al. 2011[35]	n/c	n/c	n/c	n/c	n/c	n/c	n/c	n/c	n/c	n/c	n/c	n/c	not assessed
Regalado et al. 2011[36]	10	34	18	9	0	6	1	4	0	0	0	0	not assessed
Renard et al. 2013[37]	21	22	34	12	5	30	6	7	7	3	4	16	not assessed
Robertson et al. 2016[38]	341	56	n/c	255	n/c	n/c	48	n/c	n/c	38	n/c	n/c	not assessed
Sherrah et al. 2016[39]	n/a	n/a	n/c	n/c	n/c	n/c	n/c	n/c	n/c	n/c	n/c	n/c	not assessed
Takeda et al. 2015[40]	4	24	5	2	0	6	2	0	2	0	2	3	assessed
Teixidó-Turà et al. 2014[41]	2	6	8	0	3	5	1	1	15	1	4	n/c	not assessed
Tortora et al. 2017[42]	5	21	77	5	n/c	n/c	n/c	n/c	n/c	n/c	n/c	n/c	not assessed
Tran-Fadulo et al. 2006[43]	18	12	14	6	0	45	6	11	63	6	16	28	not assessed
Tran-Fadulo et al. 2009[44]	26	33	31	13	1	23	9	2	4	4	0	13	assessed

**Table S5 (Continued)**													
**Study (Author/Year)**	**Newly diagnosed affected relatives (aneurysm+dissection)**		**FIRST DEGREE RELATIVES**			**SECOND DEGREE RELATIVES**			**THIRD DEGREE RELATIVES**			**Spouse**	
	**N.**	**%.**	**N.**	**Affected N.**	**Not Screened***	**N.**	**Affected N.**	**Not Screened**	**N.**	**Affected N.**	**Not Screened**	**N.**	**Screened**
Vaughan et al. 2001[45]	27	40	27	17	1	20	9	0	2	1	0	15	assessed
Wang et al. 2010[46]	n/c	n/c	n/c	n/c	n/c	n/c	n/c	n/c	n/c	n/c	n/c	n/c	assessed
Wang et al. 2013[47]	1	10	7	1	0	0	0	0	0	0	0	1	not assessed
Ware et al. 2014[48]	0	0	4	0	0	0	0	0	0	0	0	1	not assessed
Warnes et al. 1985[49]	0	0	4	0	0	0	0	0	0	0	0	1	assessed
Weigang et al. 2007[50]	n/c	n/c	n/c	n/c	n/c	n/c	n/c	n/c	n/c	n/c	n/c	n/c	assessed
Yoo et al. 2010[51]	4	20	7	3	1	7	1	0	0	0	0	5	not assessed
Zhu et al. 2006[52]	n/c	n/c	n/c	n/c	n/c	n/c	n/c	n/c	n/c	n/c	n/c	n/c	assessed
Ziganshin et al. 2015[53,†]	3	11	7	3	1	11	0	8	2	0	2	6	not assessed
Ziganshin et al. 2015[53,†]	6	35	6	4	1	8	2	5	0	0	0	2	not assessed

n/a indicates not available; n/c, not computable.
*Relatives not screened: not available, deceased or not eligible for screening. †Data of two different screened families obtained from the same study.

Table S6. Full details of the screened families and relatives with reference to additional observed cardiovascular diseases and physical features

Study (Author/Year)	NS-TAD Form	Subjects screened			(Associated) Cardiovascular disease			(Associated) Physical features		
		N.	% (pedigree*)	% (eligible†)	Type	N.	%*	Type	N.	%*
Barbier et al. 2014[1]	FTAAD	13	33	37	Arterial tortuosity, MVP, AF	6	15	Pectus, ARAC, High arched palate	5	13
Bee et al. 2012[2]	FTAA	32	59	73	-	-	-	Pectus, Joint hypermobility	14	26
Chamney et al. 2015[3]	FTAAD	6	43	55	-	-	-	Iris flocculi	4	29
Disabella et al. 2011[4]	FTAAD	19	51	83	ICA, Coronary artery dissection	3	8	IH, varicose vein, Iris flocculi, Iris hypoplasia, Myopia, Cornea plana, Spontaneous pneumothorax, Scoliosis, Joint laxity, Pes planus, Livedo reticularis, Cheloid scars	16	43
Disertori et al. 1991[5]	FTAAD	14	47	58	-	-	-	Joint hyperextensibility	14	47
Dong et al. 2014[6]	FTAAD	39	61	74	AAA	1	2	-	-	-
Francke et al. 1995[7]	FTAAD	23	88	100	MVP, atrial myxoma	2	8	Pectus, Joint hyperextensibility, Myopia, Dental crowding	10	38
Gago-Diaz et al. 2014[8]	FTAAD	12	39	55	BAV	1	3	Joint laxity, Scoliosis, Dolichocephaly	3	10
Gago-Diaz et al. 2016[9]	FTAAD	14	47	56	-	-	-	Pectus, Skin striae, Myopia, Scoliosis, Wrist and thumb sign	7	23
Guo et al. 2001[10]	FTAAD	121	55	86	-	-	-	-	ns	-
Guo et al. 2007[11]	FTAAD	130	61	86	PDA, BAV, ICA	10	5	Livedo reticularis, iris flocculi	41	19
Guo et al. 2009[12]	FTAAD	163	61	93	BAV, CAD, Moyamoya disease	33	12	Livedo reticularis	17	6
Guo et al. 2011[13]	FTAAD/pAA	18	64	82	pAA	3	11	-	-	-
Guo et al. 2013[14]	FTAAD	39	44	80	Coronary artery dissection, CAA, tortuosity of aorta	5	6	-	0	0
Guo et al. 2015[15]	BAV/TAA	34	71	97	BAV	4	8	-	4	0

Table S6 (Continued)										
**Study (Author/Year)**	**NS-TAD Form**	**Subjects screened**			**(Associated) Cardiovascular disease**			**(Associated) Physical features**		
		**N.**	**% (pedigree*)**	**% (eligible†)**	**Type**	**N.**	**%***	**Type**	**N.**	**%***
Guo et al. 2016[16]	FTAAD	21	32	55	AAA, BAV	5	8	Pectus, Palatus, Dolichostenomelia, Joint laxity/hypermobility, Skin striae, Dural ectasia	ns	-
Hannuksela et al. 2015[17]	FTAAD	106	40	79	-	-	-	-	-	-
Hannuksela et al. 2016[18]	FTAAD	19	41	61	ICA	2	4	-	-	-
Harakalova et al. 2013[19]	TAAD/PDA	40	53	85	PDA	5	7	-	0	0
Hasham et al. 2003[20]	FTAAD	52	75	85	BAV, Coarc	1	1	Pectus, ARAC, Palatus	6[‡]	9
Kakko et al. 2003[21]	FTAAD	115	54	77	AAA	3	1	ns	0	0
Kent et al. 2013[22]	BAV/TAA	93	72	99	BAV, Coarc, UAV, HLHS, ASD, VSD, TGA, PFO, LCA	25	19	-	0	0
Keramati et al. 2010[23]	FTAAD	15	65	75	-	-	-	-	0	0
Khau Van Kien et al. 2004[24]	FTAAD/PDA	49	72	98	PDA, ICA	13	19	-	0	0
Khau Van Kien et al. 2005[25]	FTAAD/PDA	78	84	96	PDA, ICA	13	15	-	0	0
Kuang et al. 2016[26]	FTAAD	16	40	57	-	-	-	ns	0	0
Loscalzo et al. 2007[27]	BAV/TAA	138	71	92	BAV, Coarc, UAV, HLHS, ASD, VSD, TGA, PFO, LCA	33	17	Mild join hyperextensibility	0	0
Marwick et al. 1987[28]	FTADiss	4	24	27	-	-	-	-	-	-
McManus et al. 1987[29]	FTADiss	8	42	73	-	-	-	IH, Scoliosis, Varicose vein	12	63
Milewicz et al. 1998[30]	FTAAD	ns	-	-	AAA, ICA, BAV	7	6	IH, Scoliosis	15	12
Morisaki et al. 2009[31]	FTAAD	9	19	30	ns	-	-	Iris coloboma	47	100
Pannu et al. 2005[32]	FTAAD	72	31	40	AAA, ICA, RAA, Pulmonary AA	8	3	-	-	-
Pannu et al. 2007[33]	FTAAD	23	85	96	PDA	4	15	ns	27	100
Regalado et al. 2011[34]	FTAAD/ICA	12	5	10	AAA, ICA, RAA	34	15	-	-	-
Regalado et al. 2011[35]	FTAAD/ICA/pAA	36	34	51	AAA, ICA, IAA	10	9	Osteoarthritis, Skeletal, Craniofacial, Skin	25	24

Table S6 (Continued)										
Study (Author/Year)	NS-TAD Form	Subjects screened			(Associated) Cardiovascular disease			(Associated) Physical features		
		N.	% (pedigree*)	% (eligible†)	Type	N.	%*	Type	N.	%*
Regalado et al. 2011[36]	FTAAD	11	38	69	AAA, IAA	1	3	ARAC, Skin striae, Myopia	6	21
Renard et al. 2013[37]	FTAAD	29	30	43	AAA, PDA, PS	7	7	Skin translucency	3	3
Robertson et al. 2016[38]	FTAAD	581	46	58	-	-	-	-	-	-
Sherrah et al. 2016[39]	FTAAD	119	-	-	-	-	-	-	-	-
Takeda et al. 2015[40]	FTAAD	9	53	75	-	-	-	-	-	-
Teixidó-Turà et al. 2014[41]	FTAAD	10	28	40	-	-	-	-	-	-
Tortora et al. 2017[42]	BAV/TAA	77	-	-	-	-	-	-	-	-
Tran-Fadulo et al. 2006[43]	FTAAD	9	6	8	AAA, ICA, PFO	5	3	-	-	-
Tran-Fadulo et al. 2009[44]	FTAAD	49	63	79	AAA, ICA, HAA	7	9	Skeletal	9	12
Vaughan et al. 2001[45]	FTAA	45	67	74	AAA, LSA	-	-	-	4	6
Wang et al. 2010[46]	FTADiss	21	44	62	-	-	-	-	-	-
Wang et al. 2013[47]	FTAAD	7	70	100	-	-	-	-	-	-
Ware et al. 2014[48]	FTAAD	7	100	100	AAA, ICA	1	100	Mydriasis	2	29
Warnes et al. 1985[49]	FTAAD	2	33	50	-	-	-	-	0	0
Weigang et al. 2007[50]	FTAAD	23	88	100	-	-	-	-	0	0
Yoo et al. 2010[51]	FTAAD	6	30	33	-	-	-	ns	0	0
Zhu et al. 2006[52]	FTAAD/PDA	49	100	100	PDA	3	6	-	-	-
Ziganshin et al. 2015[53,§]	FTAAD	10	37	42	-	-	-	-	-	-
Ziganshin et al. 2015[53, §]	FTAAD	15	29	63	-	-	-	-	-	-

AAA indicates abdominal aorta aneurysm; AF, atrial fibrillation; ARAC, arachnodactyly; ASD, atrial septal defect; BAV, bicuspid aortic valve; CAA, coronary artery aneurysm; CAD, coronary artery disease; Coarc, coarctation; FTAA, familial thoracic aortic aneurysm; FTAAD, familial thoracic aortic aneurysm and dissection; FTAD, familial thoracic aortic dissection; HAA, aneurysm of the hepatic artery; HLHS, hypoplastic left heart syndrome; IAA, aneurysm of the iliac artery; ICA, intracranial aneurysm; IH, inguinal hernia; LCA, left cerebral artery aneurysm; MVP, mitral valve prolapse; ns, not specified (in the study); PA, pulmonary artery; pAA, peripheral artery aneurysm; PDA, patent ductus arteriosus; Pectus, pectus excavatum and/or carinatum; PFO, patent foramen ovale; PS, pulmonary stenosis; RAA, aneurysm of the renal artery; TGA, transposition of the great arteries; UAV, unicommissural aortic valve; VSD, ventricular septal defect.
*Percentage calculated considering the number of relatives in the entire family pedigree (as per protocol). †Percentage considered among eligible relatives for screening (as per protocol). [‡]Only six family relatives were evaluated. [§]Data of two different screened families obtained from the same study.

Table S7. Details of the adopted imaging modalities for the screening of relatives

Study (Author/Year)	NS-Form	Screening Type	Imaging modality of the aorta				
			TTE	CT	MR	Aortic size cut-off (mm)*	Location cut-off
Barbier et al. 2014[1]	FTAAD	GENETIC+IMAGING	yes	no	no	ns	ns
Bee et al. 2012[2]	FTAA	GENETIC	no	no	no	-	-
Chamney et al. 2015[3]	FTAAD	GENETIC+IMAGING	ns	ns	ns	-	-
Disabella et al. 2011[4]	FTAAD	GENETIC+IMAGING	yes	yes	no	Z-score value ≥ 2.5 (nomograms by Roman et al.[54])	AA/SV/STJ/Asc/Arch/Desc/ Abd Aorta
Disertori et al. 1991[5]	FTAAD	IMAGING	yes	no	no	Ns	ns
Dong et al. 2014[6]	FTAAD	GENETIC+IMAGING	yes	yes	no	42 mm (adults); z score>2 (children)	AR
Francke et al. 1995[7]	FTAAD	GENETIC+IMAGING	yes	no	no	Ns	AR
Gago-Diaz et al. 2014[8]	FTAAD	GENETIC	no	no	no	Asc Aorta > 21mm/m^2	Asc
Gago-Diaz et al. 2016[9]	FTAAD	GENETIC	no	no	no	-	-
Guo et al. 2001[10]	FTAAD	GENETIC	no	no	no	SV plotted against nomograms derived from Roman et al.[54]	SV
Guo et al. 2007[11]	FTAAD	GENETIC	no	no	no	-	-
Guo et al. 2009[12]	FTAAD	GENETIC	no	no	no	Z-score value > 2 (nomograms by Roman et al.[54])	Asc, STJ, SV
Guo et al. 2011[13]	FTAAD/pAA	GENETIC	no	no	no	≥ 42 mm	AA/SV/STJ/Asc
Guo et al. 2013[14]	FTAAD	GENETIC	no	no	no	-	-
Guo et al. 2015[15]	BAV/TAA	GENETIC	no	no	no	-	-
Guo et al. 2016[16]	FTAAD	GENETIC	no	no	no	-	-
Hannuksela et al. 2015[17]	FTAAD	GENETIC+IMAGING	yes	no	yes	Z-score >2	SV/Asc
Hannuksela et al. 2016[18]	FTAAD	GENETIC+IMAGING	yes	yes	yes	TTE measures plotted against nomograms derived from Mirea et al.[55]; MRI data against nomograms derived from Davis et al.[56]	TTE-SV and widest level of Asc; MRI - Asc and Desc at the level of pulmonary bifurcation
Harakalova et al. 2013[19]	TAAD/PDA	GENETIC	no	no	no	42	SV/Asc
Hasham et al. 2003[20]	FTAAD	GENETIC+IMAGING	yes	no	no	TTE measures plotted against the nomogram derived from Roman et al.[54]	AR/SV/SAR

Table S7 (Continued)							
Study (Author/Year)	NS-Form	Screening Type	Imaging modality of the aorta				
			TTE	CT	MR	Aortic size cut-off (mm)*	Location cut-off
Kakko et al. 2003[21]	FTAAD	GENETIC+IMAGING	yes	no	no	TTE measures plotted against the nomogram derived from Vasan et al.[57]	AR
Kent et al. 2013[22]	BAV/TAA	GENETIC+IMAGING	yes	no	no	z score ≥ 2 (nomograms of Roman et al.[54])	AR/Asc
Keramati et al. 2010[23]	FTAAD	GENETIC+IMAGING	yes	no	yes[†]	36	AR/SV/SAR
Khau Van Kien et al. 2004[24]	FTAAD/PDA	GENETIC+IMAGING	yes	no	yes	TTE measures plotted against the nomogram derived from Roman et al.[54]	SV/STJ/Asc/HA/Isthmus/Dec
Khau Van Kien et al. 2005[25]	FTAAD/PDA	GENETIC+IMAGING	yes	no	Yes[‡]	TTE measures plotted against the nomogram derived from Vasan et al.[57]	SV/STJ/Asc/HA/Isthmus/Dec
Kuang et al. 2016[26]	FTAAD	GENETIC	no	no	no	-	-
Loscalzo et al. 2007[27]	BAV/TAA	GENETIC+IMAGING	yes	no	no	z score ≥ 2 (nomograms of Roman et al.[54])	AA/AR/STJ/Asc
Marwick et al. 1987[28]	FTADiss	IMAGING	yes	no	no	Ns	ns
McManus et al. 1987[29]	FTADiss	IMAGING	yes	yes	no	Ns	ns
Milewicz et al. 1998[30]	FTAAD	GENETIC+IMAGING	yes	no	no	TTE measures plotted against the nomogram derived from Roman et al.[54]	ns
Morisaki et al. 2009[31]	FTAAD	GENETIC	no	no	no	-	-
Pannu et al. 2005[32]	FTAAD	GENETIC+IMAGING	yes	no	no	TTE measures plotted against the nomogram derived from Roman et al.[54]	AR/SV/SAR
Pannu et al. 2007[33]	FTAAD	GENETIC+IMAGING	yes	yes	yes	TTE measures plotted against the nomogram derived from Roman et al.[54]	SV/SAR/Asc
Regalado et al. 2011[34]	FTAAD/ICA	GENETIC	no	no	no	TTE measures plotted against the nomogram derived from Roman et al.[54]	AA/SV/STJ/Asc
Regalado et al. 2011[35]	FTAAD/ICA/pAA	GENETIC	no	no	no	TTE measures plotted against the nomogram derived from Roman et al.[54]	AA/SV/STJ/Asc
Regalado et al. 2011[36]	FTAAD	GENETIC	no	no	no	TTE measures plotted against the nomogram derived from Roman et al.[54]	AA/SV/STJ/Asc
Renard et al. 2013[37]	FTAAD	GENETIC	no	no	no	Z-score >3	SV/Asc
Robertson et al. 2016[38]	FTAAD	IMAGING	yes	yes	yes	Aortic index and Z-score	SV/Asc
Sherrah et al. 20016[39]	FTAAD	IMAGING	yes	yes	yes	TTE measures (z score ≥ 2) plotted against the nomograms from Wolak et al.[58]	SV/Asc
Takeda et al. 2015[40]	FTAAD	GENETIC	no	no	no	-	-

Table S7 (Continued)							
**Study (Author/Year)**	**NS-Form**	**Screening Type**	**Imaging modality of the aorta**				
			**TTE**	**CT**	**MR**	**Aortic size cut-off (mm)***	**Location cut-off**
Teixidó-Turà et al. 2014[41]	FTAAD	GENETIC	no	no	no	-	-
Tortora et al. 2017[42]	BAV/TAA	GENETIC+IMAGING	Yes	no	no	-	-
Tran-Fadulo et al. 2006[43]	FTAAD	GENETIC	no	no	no	TTE measures plotted against the nomogram derived from Roman et al.[54]	SV/AR/SAR/Asc
Tran-Fadulo et al. 2009[44]	FTAAD	GENETIC	no	no	no	TTE measures plotted against the nomogram derived from Roman et al.[54]	SV/AR/SAR/Asc
Vaughan et al. 2001[45]	FTAA	GENETIC+IMAGING	yes	no	no	TTE measures plotted against the nomogram derived from Roman et al.[54]	AA/SV/STJ/Asc/Arch/Desc
Wang et al. 2010[46]	FTADiss	GENETIC	no	no	no	-	-
Wang et al. 2013[47]	FTAAD	GENETIC	no	no	no	-	-
Ware et al. 2014[48]	FTAAD	GENETIC	no	no	no	-	-
Warnes et al. 1985[49]	FTAAD	IMAGING	yes	no	no	Ns	ns
Weigang et al. 2007[50]	FTAAD	GENETIC+IMAGING	yes	yes	yes	Ns	AA/SV/STJ/Asc
Yoo et al. 2010[51]	FTAAD	GENETIC	no	no	no	-	-
Zhu et al. 2006[52]	FTAAD/PDA	GENETIC+IMAGING	yes	no	no	TTE measures plotted against the nomogram derived from Roman et al.[54]	SV/STJ/Asc/HA/Isthmus/Desc
Ziganshin et al. 2015[53]	FTAAD	GENETIC	no	no	no	-	-
Ziganshin et al. 2015[53]	FTAAD	GENETIC	no	no	no	-	-

AA indicates aortic annulus; Abd, abdominal aorta; AR, aortic root; Arch, aortic arch; Asc, ascending thoracic aorta; CT, computed tomography (of the aorta); Desc, descending thoracic aorta; FTAA, familial thoracic aortic aneurysm; FTAAD, familial thoracic aortic aneurysm and dissection; FTAD, familial thoracic aortic dissection; HA, horizontal aorta; ICA, intracranial aneurysm; MR, magnetic resonance(of the aorta); ns, not specified; pAA, peripheral artery aneurysm; PDA, patent ductus arteriosus; SAR, supra-aortic ridge; STJ, sinus tubular junction; SV, sinus of Valsalva; TTE, transthoracic echocardiogram.

*For studies without prospective imaging screening, cut-off aortic size e location of aortic segment provided based on retrospective evaluation of TTE. †Limited number of relatives were subjected to MRI of lumbosacral region. ‡48 subjects undergone cine MR for assessing aortic compliance.

Table S8. Details of the adopted screening modalities in the included studies

Study (Author/Year)	NS-Form	Screening Type	Genetic analysis				
			Techniques used	Gene identified	Genetic mutations	Replicated in an independent cohort	Animal model and/or tissue validation
Barbier et al. 2014[1]	FTAAD	GENETIC+ IMAGING	Whole exome sequencing	MFAP5	c.472C>T (p.Arg158*); c.62G>T (p.Trp21Leu)	Following discover of the MFAP5 mutation in TAA-9801, mutation in MFAP5 were searched in a population of 225 familial and 178 sporadic subjects of French origin and 267 familial subjects of American origin; this led to discover another variant in TAA-9178 co-segregating with TAAD	Effects of mutation were investigated in dermal fibroblasts from affected family members. Mutation led to pure haploinsufficiency of the protein product presumably due to degradation in the endoplasmatic reticulum
Bee et al. 2012[2]	FTAA	GENETIC	Targeted sequencing of ACTA2, MYH11, TGFBR1, and TGFBR2	ACTA2, MYH11, TGFBR2	ACTA2 (p.Gly270Glu, p.Arg118Gln, p.Thr108Met); MYH11 (p.Arg1590Gln, p.Glu1899Asp, intronic 7bp substitution of TGCTTTT>G, 5bp 3' of exon 27); TGFBR2 (p.Ala414Thr, p.His56Asn, p.Asp40Asn)	no	TGFBR2 p.Ala414Thr mutation was shown to have reduced kinase activity in an *in-vitro* gene expression model; TGFBR2 p.His56Asn mutation was associated with delayed downward signalling in a skin fibroblast culture model. Rat myoblasts cells transfected with His56Asn-TGFBR2 or Asp40Asn-TGFBR2 showed reduced downward signalling when stimulated with TGF2
Chamney et al. 2015[3]	FTAAD	GENETIC+ IMAGING	Targeted sequencing	ACTA2	(p.Arg149Cys)	no	no
Disabella et al. 2011[4]	FTAAD	GENETIC+ IMAGING	Targeted sequencing	ACTA2	p.Arg149Cys, p.Asp82Glu, p.Glu243Lys, p.Val45Leu, c.IVS4+1G>A	no	Histological assessment of aortic tissue samples from individuals affected by dissection showed severe medial degeneration, smooth muscle disarray, hyperplasia of the vasa vasorum medial wall smooth muscles

Table S8 (Continued)							
**Study (Author/Year)**	**NS-Form**	**Screening Type**	**Genetic analysis**				
			**Techniques used**	**Gene identified**	**Genetic mutations**	**Replicated in an independent cohort**	**Animal model and/or tissue validation**
Disertori et al. 1991[5]	FTAAD	IMAGING	Not performed	n/a	-	-	-
Dong et al. 2014[6]	FTAAD	GENETIC+ IMAGING	Whole exome sequencing - Sanger sequencing	TGFBR1	c.1459C>T (p.Arg487Trp)	no	no
Francke et al. 1995[7]	FTAAD	GENETIC+ IMAGING	Single strand conformation analysis, allele specific oligonucleotide hybridization detection, targeted sequencing	FBN1	c.3379G>A (p.Gly1127Ser)	Attempt of replication in 64 unrelated individuals with MFS, 30 individuals with MFS-related phenotypes and 84 normal controls did not show presence of this mutation	Cultured skin fibroblasts from affected members revealed reduced fibrillin deposition to the control medium
Gago-Diaz et al. 2014[8]	FTAAD	GENETIC	Multiplex ligation dependent probe amplification - Sanger sequencing - Whole exome sequencing	TGFB2	c.1042C>T (p.Arg348Cys)	no	no
Gago-Diaz et al. 2016[9]	FTAAD	GENETIC	Multiplex ligation dependent probe amplification - Massive parallel sequencing - Whole exome sequencing	PRKG1	c.530G>A; (p.Arg177Gln)	no	no
Guo et al. 2001[10]	FTAAD	GENETIC	Genome wide linkage analysis - Targeted sequencing	Locus 5q13-14 D5S806-D5S641	n/a	no	no

Table S8 (Continued)							
**Study (Author/Year)**	**NS-Form**	**Screening Type**	**Genetic analysis**				
			**Techniques used**	**Gene identified**	**Genetic mutations**	**Replicated in an independent cohort**	**Animal model and/or tissue validation**
Guo et al. 2007[11]	FTAAD	GENETIC	Genome wide linkage analysis - Targeted sequencing	ACTA2	c.492C>t (p.R149C); c.921A>G (p.R292G); c.397A>C (p.N117T); c.664C>G (p.V154A); c.450T>C (p.Y135H); c.820G>A (p.R258C); c.819C>T (p.R258C); c.400G>A (p.R118Q); c.1105C>A (p.T353N)	The initial discover in TAA327 was followed by ACTA2 sequencing in 97 probands from FTAAD families; this led to detection of 14 further families where ACTA2 mutations co-segregated with TAAD. Other 384 healthy control subjects European descendent served as control	Histological examination of aorta specimens obtained from affected individuals revealed proteoglycan accumulation, elastin fragmentation and areas of increased smooth muscle proliferation in the tunica media of vasa vasorum. Analysis of intracellular actin filaments from mutation carriers showed disturbed actin filament stability
Guo et al. 2009[12]	FTAAD	GENETIC	Exome sequencing - Linkage analysis	ACTA2	n/a	ACTA 2 sequencing in a group of 237 sporadic TAAD patients revealed presence of heterozygous mutations in 6 subjects.	192 matched controls used. Thickening of the walls of aortic vasa vasorum vessels was observed in mutation carriers as compared to control subjects. Smooth muscle cells harvested from mutation carriers showed higher proliferation rate than smooth muscle cells harvested from age and sex matched controls
Guo et al. 2011[13]	FTAAD/pAA	GENETIC	Linkage analysis utilising 50K GeneChips Hind Array by Affymetrix - Candidate gene sequencing	Locus 12q13-14 D12S1691-D12S1726	n/a	no	Medial degeneration observed in the aortic samples from affected individual.
Guo et al. 2013[14]	FTAAD	GENETIC	Whole exome sequencing - Linkage analysis	PRKG1	c.530G>A (p.Arg177Gln)	Initial finding from pedigree TAA216 replicated in pedigrees TAA508, TAA690 and TAA292	Human embryonic kidney cells transfected with the c530G>A PRKG1 gene variant showed much higher enzymatic activity of the gene product when compared to the wild type protein (gain of function mutation)

Table S8 (Continued)							
**Study (Author/Year)**	**NS-Form**	**Screening Type**	**Genetic analysis**				
			**Techniques used**	**Gene identified**	**Genetic mutations**	**Replicated in an independent cohort**	**Animal model and/or tissue validation**
Guo et al. 2015[15]	BAV/TAA	GENETIC	Genome wide linkage analysis - Whole exome sequencing	MATA2	c.1031A>C (p.Glu344Ala)	no	447 probands use for comparison. Aortic tissue samples from two affected and mutation positive individuals showed medial degeneration in aortic media (elastin fragmentation and proteoglycan deposition)
Guo et al. 2016[16]	FTAAD	GENETIC	Exome sequencing - Sanger sequencing	LOX	c.839G>T (p.Ser280Arg); c.125G>A (p.Trp42*); c.604G>T (p.Gly202*); c.743C>T (pThr248Ile), c.800A>C (p.Gln267Pro); c1044T>A (p.Ser348Arg)	Exome and Sanger sequencing in an additional 410 unrelated FTAAD probands identified 5 additional rare, disruptive LOX variants	Decreased levels of LOX product's enzymatic activity was confirmed for three missense LOX mutations (p.Thr248Ile, p.Ser280Arg, p.Ser348Arg) in transected HeLa cell culture
Hannuksela et al. 2015[17]	FTAAD	GENETIC+ IMAGING	Targeted analysis of ACTA2, COL3A1, COL5A1, COL5A2, EFEMP2, FBN1, FBN2, GATA5, MYH11, MYLK, NOTCH1, SLCA10, SMAD3, TGFB2, TGFBR1, and TGBFR2	Not identified	-	-	-
Hannuksela et al. 2016[18]	FTAAD	GENETIC+ IMAGING	Whole exome sequencing - Sanger sequencing	MYLK	c3272_3273del (p.Ser1091*)	no	Histopathological assessment of aortic specimens from members of family affected by aortic dissection revealed discontinuation of elastic fibres; no pathological findings were present in histopathological examination of mutation carriers, who underwent prophylactic surgery

Table S8 (Continued)							
**Study (Author/Year)**	**NS-Form**	**Screening Type**	**Genetic analysis**				
			**Techniques used**	**Gene identified**	**Genetic mutations**	**Replicated in an independent cohort**	**Animal model and/or tissue validation**
Harakalova et al. 2013[19]	TAAD/PDA	GENETIC	Targeted sequencing, rare copy number variants detection with comparative genome hybridization, detection of intragenic copy number variants performed by analysis of melting curves using qPCR, genome wide linkage analysis	MYH11	MYH11 c.232A>G (p.Lys78Glu), MYH11 c.3766-68delAAG	no	-
Hasham et al. 2003[20]	FTAAD	GENETIC+ IMAGING	Genome-wide linkage analysis - Targeted sequencing of FBLN2	TAAD2	n/a	no	-
Kakko et al. 2003[21]	FTAAD	GENETIC+ IMAGING	Linkage analysis	Locus 5q13-14	n/a	no	-
Kent et al. 2013[22]	BAV/TAA	GENETIC+ IMAGING	Targeted sequencing of NOTCH1	NOTCH1	c.C3269G (p.Thr1090Ser)	no	-
Keramati et al. 2010[23]	FTAAD	GENETIC+ IMAGING	Genome wide linkage analysis - Targeted sequencing of FBN1	Locus 15q21 (FBN1?)	n/a	no	-
Khau Van Kien et al. 2004[24]	FTAAD/PDA	GENETIC+ IMAGING	Linkage analysis - Targeted sequencing of COL3A1. Seven genes and loci tested (COL3A1, FBN1, 3p24-25 or MFS2/TAAD2, 5q13-q14 and 11q23.2-q24, TFAP2B and 12q24)[a]	Not identified	n/a	no	-
Khau Van Kien et al. 2005[25]	FTAAD/PDA	GENETIC+ IMAGING	Whole genome linkage scan - Targeted sequencing	MYH11	n/a	no	-

Table S8 (Continued)							
**Study (Author/Year)**	**NS-Form**	**Screening Type**	**Genetic analysis**				
			**Techniques used**	**Gene identified**	**Genetic mutations**	**Replicated in an independent cohort**	**Animal model and/or tissue validation**
Kuang et al. 2016[26]	FTAAD	GENETIC	Exome sequencing	FOXE3	c.457G>C (p.Asp153His)	Exome sequencing was performed in a group of 564 unrelated subjects with FATAAD - 7 other rare variants predicted to disrupt the protein variants were found	Knock-out of FOXE3 in zebrafish leads to disruption of aortic arch development. Knock-out of FOXE3 in mouse embryos leads to reduced cell density in aortic media when compared to wild type
Loscalzo et al. 2007[27]	BAV/TAA	GENETIC+ IMAGING	Targeted sequencing of TGFBR1 and TGFBR2	Not identified	-	-	-
Marwick et al. 1987[28]	FTADiss	IMAGING	Not performed	-	-	-	-
McManus et al. 1987[29]	FTADiss	IMAGING	Not performed	-	-	-	-
Milewicz et al. 1998[30]	FTAAD	GENETIC+ IMAGING	Targeted linkage for FBN1 locus and 3p24-25 locus	No linkage to FBN1 or TAAD2	n/a	no	-
Morisaki et al. 2009[31]	FTAAD	GENETIC	Targeted sequencing of ACTA2	ACTA2	c.445C>T (p.Arg.149Cys); c.616+1G>T (p.Gly152_Thr205 del); c.635G>A (p.Arg212Cys)	no	-
Pannu et al. 2005[32]	FTAAD	GENETIC+ IMAGING	Targeted sequencing of TGFBR2, Targeted linkage analysis	TGFBR2	c.1378C>T (p.Arg460Cys); c.1379G>A (p.Arg460His)	yes	-
Pannu et al. 2007[33]	FTAAD	GENETIC+ IMAGING	Targeted sequencing of MYH11	MYH11	c.3791T > C (p.Leu1264Pro); c.3824G > T p.Arg1275Leu)	yes	Cystic medial degeneration was present in aortic tissue of subject with MYH11 mutations
Regalado et al. 2011[34]	FTAAD/ICA	GENETIC	Targeted sequencing of ACTA2, TGFBR1, and TGFBR2	ACTA2, TGFBR1, TGFBR2	ACTA2 p.Arg258Cys, TGFBR1 p.Arg487Trp, TGFBR2 p.Arg460His,	no	-

Table S8 (Continued)							
**Study (Author/Year)**	**NS-Form**	**Screening Type**	**Genetic analysis**				
			**Techniques used**	**Gene identified**	**Genetic mutations**	**Replicated in an independent cohort**	**Animal model and/or tissue validation**
Regalado et al. 2011[35]	FTAAD/ICA/pAA	GENETIC	Whole exome sequencing - Linkage analysis	SMAD3	c.652delA (p.Asn218fs); exone 6 c.836G>A (p.Arg279Lys); exone 6 c.715G>A (p.Glu239Lys); exon 2 c.235C>T (p.Ala112Val)	yes	-
Regalado et al. 2011[36]	FTAAD	GENETIC	Exome sequencing - Sanger sequencing	FBN1	c.7656C>A (p.Cys2552Ter); c.7039_7040delAT (p.Met2347Valfs*19); c.813C>G (p.Cys271Trp); c.6866G>T (p.Cys2289Phe); c.4467T>A (p.Asn1489Lys)	no	-
Renard et al. 2013[37]	FTAAD	GENETIC	Targeted sequencing of ACTA2 and MYH11[†]	ACTA2, MYH11	ACTA2 c.940C>T (p.Arg314X); ACTA2 c.1019_1020delCT(p.Ser340 Cys fs X25); ACTA2 c.124C>A (p.His42Asn); ACTA2 c. 115C>T (p.Arg39Cys); ACTA2 c.145G>A (p.Met49Val), ACTA2 c.112G>A (p.Gly38Arg), ACTA2 c.182A>G (p.Gln61Arg); MYH11 intron 4 IVS32+1G>A	no	Histological examination of tissue samples from patients with ACTA2 and MYH11 mutations revealed medial degeneration. Increased expression of TGFB pathway was observed in individuals with MYH11 mutation
Robertson et al. 2016[38]	FTAAD	IMAGING	Not performed	-	-	-	-
Sherrah et al. 2016[39]	FTAAD	IMAGING	Not performed	-	-	-	-

Table S8 (Continued)							
**Study (Author/Year)**	**NS-Form**	**Screening Type**	**Genetic analysis**				
			**Techniques used**	**Gene identified**	**Genetic mutations**	**Replicated in an independent cohort**	**Animal model and/or tissue validation**
Takeda et al. 2015[40]	FTAAD	GENETIC	Targeted sequencing of ACTA2, FBN1, MYH11, SMAD3, TGFB, TGFBR1, and TGFBR2	MYH11	c.3791T>C(p.Leu1264Pro)	no	-
Tortora et al. 2017[42]	BAV/TAA	GENETIC+ IMAGING	Targeted sequencing of ABCC9, ACTA2, CBL, ELN, FBN1, FBN2, MYH11, MYH7, MILK, NOTCH1, TGFB2, TGFB3, TGFBR1 and TGFBR2		n/a	no	-
Teixidó-Turà et al. 2014[42]	FTAAD	GENETIC	ns	ACTA2	c.253G>A (p.Glu85Lys)	no	-
Tran-Fadulo et al. 2006[42]	FTAAD	GENETIC	TaqMan genotyping, Linkage analysis of FBN1, TAAD1, TAAD2, and FAA1 loci, Targeted sequencing of TGFBR2[‡]	Not identified	n/a	no	-
Tran-Fadulo et al. 2009[44]	FTAAD	GENETIC	Targeted sequencing of TGFBR1	TGFBR1	TGFBR1 exon 9 c.1459C>T (p.Arg487WTrp); TGFBR1 exon 9 c.1457T>C (p.Leu486Ser), TGFBR1 exon 5 c.944A>G, p.His315Arg; TGFBR1 exon5 c.934G>A, (p.Gly312Ser)	yes	-
Vaughan et al. 2001[45]	FTAA	GENETIC+ IMAGING	Linkage analysis of known loci (FBN1, FBN2, COL3A1, MFS2, 5q-TAA, FAA1) - Whole genome linkage analysis - Targeted sequencing of SM22α, HSP73	Locus 11q23.3-q24 D11S1341-AFMB031 WC9 (FAA1?)	n/a	no	-

Table S8 (Continued)							
**Study (Author/Year)**	**NS-Form**	**Screening Type**	**Genetic analysis**				
			**Techniques used**	**Gene identified**	**Genetic mutations**	**Replicated in an independent cohort**	**Animal model and/or tissue validation**
Wang et al. 2010[46]	FTADiss	GENETIC	Targeted sequencing of CALM1, MYLK, MYL6, MYL6B, and MYL9 - Linkage analysis	MYLK	MYLK c.5275T>C (p.Ser1759Pro), MYLK c.4438C>T (p.Arg1480X)	no	Mutant products of the MYLK gene showed reduced affinity to calmodulin in transfected cells. Mice with tamoxifen-induced smooth muscle cell specific MYLK knock out showed accumulation of proteoglycan in the aortic media
Wang et al. 2013[47]	FTAAD	GENETIC	Targeted sequencing of FBN1, TGFBR1 and TGFBR2	Not identified	n/a	yes	-
Ware et al. 2014[48]	FTAAD	GENETIC	Targeted sequencing of ACTA2, FBN1, MYH11, TGFBR1 and TGFBR2	ACTA2	p.Lys328Asn	no	-
Warnes et al. 1985[49]	FTAAD	IMAGING	Not performed	-	-	-	-
Weigang et al. 2007[50]	FTAAD	GENETIC+ IMAGING	PCR	Not identified	Tested for FBN1, negative	no	-
Yoo et al. 2010[51]	FTAAD	GENETIC	Targeted sequencing of ACTA2, FBN1, and TGFBR2	ACTA2	exone 2 c.76G>T (p.Asp26Tyr)	no	-
Zhu et al. 2006[52]	FTAAD/PDA	GENETIC+ IMAGING	Linkage analysis - Targeted sequencing	MYH11	Substitution at a splice donor site of intron 32 (IVS32+1G→T); c.3810-3881del (p.Arg1241-Leu1264del)	no	Analysis of fibroblast culture obtained from mutation careers showed that transcript of a gene with splice donor site substitution led to production of cDNA without exon 32, which led to deletion of a 71 amino acids in the C-terminal region of the protein; aortic tissue samples from affected members revealed cystic medial degeneration, carriers of the mutation showed reduced aortic compliance
Ziganshin et al. 2015[53]	FTAAD	GENETIC	Whole exome sequencing	MYLK	MYLK p.Ser1759Pro	no	-
Ziganshin et al. 2015[53]	FTAAD	GENETIC	Whole exome sequencing	TGFBR1	TGFBR1 p.Gly188Val	no	-

AD indicates autosomal dominant; FTAA, familial thoracic aortic aneurysm; FTAAD, familial thoracic aortic aneurysm and dissection; FTAD, familial thoracic aortic dissection; GEN, genetic; ICA, intracranial aneurysm; IMAG, imaging; n/a, not available; pAA, peripheral artery aneurysm.

*Seven genes and loci tested (COL3A1, FBN1, 3p24-25 or MFS2/TAAD2, 5q13-q14 and 11q23.2-q24, TFAP2B 12q24): negative correlations. †ACTA2 positive in TAAD isolated; MYH11 positive in family with TAAD and PDA. ‡Relatives from family TAA216 tested for TAAD1, TAAD2, FAA1 and FBN1 with negative correlation, other three relatives from families TAA216, TAA105 and TAA174 tested for TGFBR2 with negative correlation.

Table S9. Quality assessment of the included studies

Study (Author/Year)	Newcastle-Ottawa Scale[58]			Cochrane Risk of Bias Analysis[59]					USPSTF design-specific quality criteria[60]
	Selection	Comparability	Outcome	Selction Bias	Perfomance Bias	Detection Bias	Attrition Bias	Reporting Bias	
Barbier et al. 2014[1]	**	**	*	Low	Low	High	High	High	Fair
Bee et al. 2012[2]	*	**	***	High	High	High	High	High	Fair
Chamney et al. 2015[3]	*	*	**	High	Low	Low	High	High	Poor
Disabella et al. 2011[4]	***	**	***	Low	Low	Low	Low	Low	Fair
Disertori et al. 1991[5]	-	-	*	Unclear	High	Unclear	High	Unclear	Poor
Dong et al. 2014[6]	***	**	***	Low	Low	Low	Low	Low	Fair
Francke et al. 1995[7]	**	*	***	Low	Low	Low	Low	Low	Poor
Gago-Diaz et al. 2014[8]	***	**	***	High	High	Low	High	High	Poor
Gago-Diaz et al. 2016[9]	**	**	**	High	High	Low	High	High	Fair
Guo et al. 2001[10]	*	*	***	High	High	High	Low	Low	Fair
Guo et al. 2007[11]	**	*	***	High	High	Low	Low	Low	Fair
Guo et al. 2009[12]	***	*	***	High	High	High	Low	Low	Fair
Guo et al. 2011[13]	***	*	***	High	High	Low	Low	Low	Fair
Guo et al. 2013[14]	***	*	***	High	High	Low	Low	Low	Fair
Guo et al. 2015[15]	***	*	***	High	High	High	Low	Low	Fair
Guo et al. 2016[16]	***	*	**	High	High	Low	High	High	Fair
Hannuksela et al. 2015[17]	***	*	***	Low	Low	High	High	High	Fair
Hannuksela et al. 2016[18]	***	**	**	Low	Low	Low	High	High	Fair
Harakalova et al. 2013[19]	***	**	***	High	High	Low	High	High	Fair
Hasham et al. 2003[20]	***	**	***	Low	Low	Low	Low	Low	Fair
Kakko et al. 2003[21]	***	**	**	Low	Low	Low	High	High	Poor
Kent et al. 2013[22]	**	**	***	Low	Low	High	High	High	Fair
Keramati et al. 2010[23]	**	*	**	Low	Low	High	High	High	Fair
Khau Van Kien et al. 2004[24]	***	**	***	Low	Low	High	High	High	Poor
Khau Van Kien et al. 2005[25]	***	**	***	Low	Low	High	Low	Low	Fair
Kuang et al. 2016[26]	***	**	*	High	High	Low	High	High	Fair
Loscalzo et al. 2007[27]	***	*	***	Low	Low	High	High	High	Fair
Marwick et al. 1987[28]	-	*	*	Unclear	Unclear	Unclear	Unclear	Unclear	Poor

McManus et al. 1987[29]	-	*	**	Unclear	High	Unclear	Unclear	Unclear	Poor
Milewicz et al. 1998[30]	**	*	**	Low	Low	Low	Low	Low	Fair
Morisaki et al. 2009[31]	**	*	*	High	High	Low	High	High	Fair
Pannu et al. 2005[32]	***	**	*	Low	Low	Low	High	High	Fair
Pannu et al. 2007[33]	***	**	***	Low	Low	Low	Low	Low	Fair
Regalado et al. 2011[34]	**	**	*	High	High	Low	High	High	Fair
Regalado et al. 2011[35]	**	**	**	High	High	High	High	High	Fair
Regalado et al. 2011[36]	**	**	**	High	High	Low	High	High	Fair
Renard et al. 2013[37]	**	**	**	High	High	Low	High	High	Fair
Robertson et al. 2016[38]	***	**	***	Unclear	High	Low	Unclear	Unclear	Good
Sherrah et al. 2016[39]	***	**	***	Unclear	High	High	Unclear	Unclear	Fair
Takeda et al. 2015[40]	**	**	**	High	High	Low	High	High	Fair
Teixidó-Turà et al. 2014[41]	*	*	*	High	High	Low	High	High	Fair
Tortora et al. 2017[42]	*	*	*	Unclear	High	High	High	High	Poor
Tran-Fadulo et al. 2006[42]	**	*	**	High	High	Low	High	High	Fair
Tran-Fadulo et al. 2009[43]	**	**	**	High	High	High	High	High	Fair
Vaughan et al. 2001[44]	***	**	***	Low	Low	High	Low	Low	Fair
Wang et al. 2010[45]	**	*	**	High	High	Low	High	High	Fair
Wang et al. 2013[46]	**	**	**	High	High	High	Low	Low	Fair
Ware et al. 2014[47]	*	*	*	High	High	High	Low	Low	Poor
Warnes et al. 1985[48]	-	*	*	Unclear	Unclear	Unclear	High	High	Poor
Weigang et al. 2007[49]	***	**	**	Low	Low	High	Low	Low	Poor
Yoo et al. 2010[50]	**	**	**	High	High	Low	High	High	Fair
Zhu et al. 2006[51]	***	**	**	Low	Low	High	Low	Low	Poor
Ziganshin et al. 2015[52]	**	*	*	High	High	High	High	High	Poor

USPSTF indicates US Preventive Services Task Force.

Table S10. Genetic architecture of thoracic aortic diseases in non-syndromic forms after screening of the family relatives

Locus	Gene			NS-TAD form	OMIM		Associated TAD	Supporting Reference
	Name	LOCUS OMIM n.	Role		Phenotype	n.		
Known genes								
1q41	TGFB2	Unassigned	TGF-β pathway	FTAAD	LDS type 4	614816	LDS type 4	8
1p33	FOXE3	601094	SMC metabolism	FTAA	AAT11	617349	-	26
2p11.2	MAT2A	Unassigned	SMC metabolism	BAV/TAA	-	Unassigned	-	15
3p24-25	TGFBR2	190182	TGF-β pathway	FTAAD	AAT3	610168	LDS type 2	8,32,34
3q21.1	MYLK	600922	Proteins involved in SMC contraction	FTAAD, FTADiss	AAT7	613780	-	18,47,53
5q23	LOX	Unassigned	ECM proteins	FTAAD	AAT10	617168	-	16
9q22.33	TGFBR1	190181	TGF-β pathway	FTAAD	AAT5	609192	LDS type 1	6,34,44,53
9q34.3	NOTCH1	190198	Neural crest migration	BAV/TAA	AVD1	109730	-	22
10q11.2-q21.1	PRKG1	176894	Proteins involved in SMC contraction	FTAAD	AAT8	615436	-	9,14
10q23.31	ACTA2	102620	Proteins involved in SMC contraction	FTAA, FTAAD	AAT6	611788	-	3,11,12,34,37, 41,48,51
12p13.31	MFAP5	601103	ECM protein	FTAAD	AAT9	616166	-	1
15q21	FBN1	154700	ECM protein	FTAAD	-	154700	MFS	7,23,36
15q22.33	SMAD3	603109	TGF-β pathway	FTAAD/ICA/pAA	-	613795	LDS type 3	34
16p13.12	MYH11	160745	Proteins involved in SMC contraction	FTAAD, FTAAD/PDA	AAT4	132900	-	19,25,33,37,40, 52
Mapped loci without identified gene								
5q13-14	-	-	-	FTAAD	AAT2	607087	-	10
11q23.3-24	-	-	-	FTAA	AAT1	607086	-	45
12q13-14	-	-	-	FTAAD/pAA	-	Unassigned	-	13

AOS indicates osteoarthritis syndrome; AVD, aortic valve disease; BAV, bicuspid aortic valve; ECM, extracellular matrix; FTAA, familial thoracic aortic aneurysm; FTAAD, familial thoracic aortic aneurysm and dissection; FTAD, familial thoracic aortic dissection; ICA, intracranial aneurysm; LDS, Loeys-Dietz syndrome; MFS, Marfan syndrome; pAA, peripheral artery aneurysm; PDA, patent ductus arteriosus; SMC, smooth muscle cell; TGF, transforming growth factor.

Table S11. Current guidelines for diagnosis and treatment of aortic diseases

**2010 ACCF/AHA/AATS/ACR/ASA/SCA/SCAI/SIR/STS/SVM guidelines**[62]		
**Recommendations**	**Class**	**Level of evidence**
Familial thoracic aortic aneurysm and dissections		
Aortic imaging is recommended for first-degree relatives of patients with thoracic aortic aneurysm and/or dissection to identify those with asymptomatic disease.	**I**	**B**
If the mutant gene (FBN1, TGFBR1, TGFBR2, COL3A1, ACTA2, MYH11) associated with aortic aneurysm and/or dissection is identified in a patient, first-degree relatives should undergo counseling and testing. Then, only the relatives with the genetic mutation should undergo aortic imaging.	**I**	**C**
If one or more first-degree relatives of a patient with known thoracic aortic aneurysm and/or dissection are found to have thoracic aortic dilatation, aneurysm, or dissection, then imaging of second-degree relatives is reasonable	**IIa**	**B**
Sequencing of the ACTA2 gene is reasonable in patients with a family history of thoracic aortic aneurysms and/or dissections to determine if ACTA2 mutations are responsible for the inherited predisposition	**IIa**	**B**
Sequencing of other genes known to cause familial thoracic aortic aneurysms and/or dissection (TGFBR1, TGFBR2, MYH11) may be considered in patients with a family history and clinical features associated with mutations in these genes	**IIb**	**B**
If one or more first-degree relatives of a patient with known thoracic aortic aneurysm and/or dissection are found to have thoracic aortic dilatation, aneurysm, or dissection, then referral to a geneticist may be considered	**IIb**	**C**
Bicuspid aortic valve and thoracic aortic disease		
First-degree relatives of patients with a bicuspid aortic valve, premature onset of thoracic aortic disease with minimal risk factors, and/or a familial form of thoracic aortic aneurysm and dissection should be evaluated for the presence of a bicuspid aortic valve and asymptomatic thoracic aortic disease	**I**	**B**
**2014 ESC Guidelines**[63]		
Familial thoracic aortic aneurysm and dissections		
It is recommended to investigate first-degree relatives (siblings and parents) of a subject with TAAD to identify a familial form in which relatives all have a 50% chance of carrying the family mutation/disease	**I**	**C**
Once a familial form of TAAD is highly suspected, it is recommended to refer the patient to a geneticist for family investigation and molecular testing	**I**	**C**
Variability of age of onset warrants screening every 5 years of 'healthy' at-risk relatives until diagnosis (clinical or molecular) is established or ruled out	**I**	**C**
In familial non-syndromic TAAD, screening for aneurysm should be considered, not only in the thoracic aorta, but also throughout the arterial tree (including cerebral arteries)	**IIa**	**C**
Bicuspid aortic valve and thoracic aortic disease		
Because of familial occurrence, screening of first-degree relatives should be considered	**IIb**	**C**

Figure S1. PRISMA flow diagram of search strategy (through December 31, 2017)

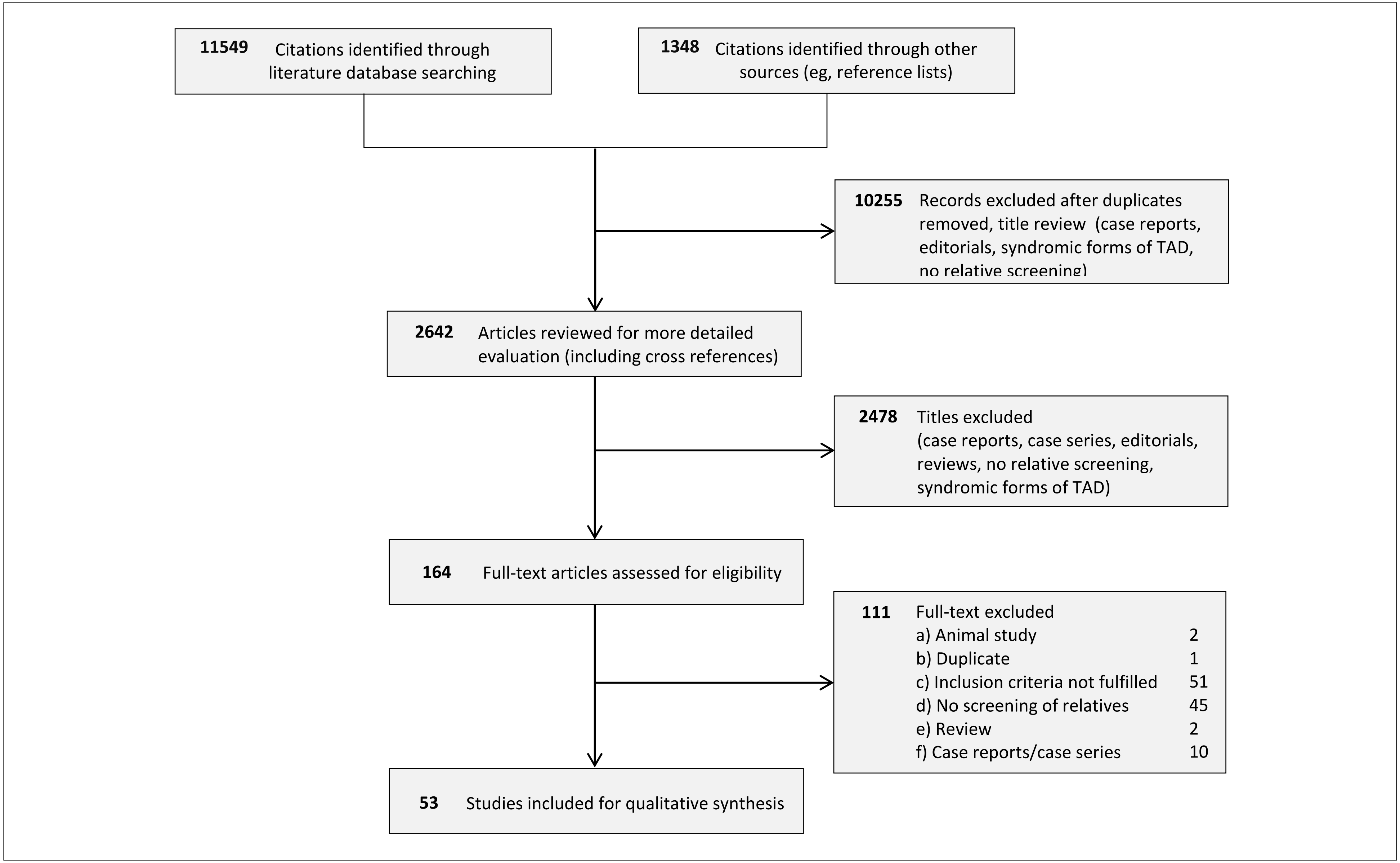

Figure S2. Genes with established causative association with non-syndromic thoracic aortic aneurysms and dissection identified in the present systematic review

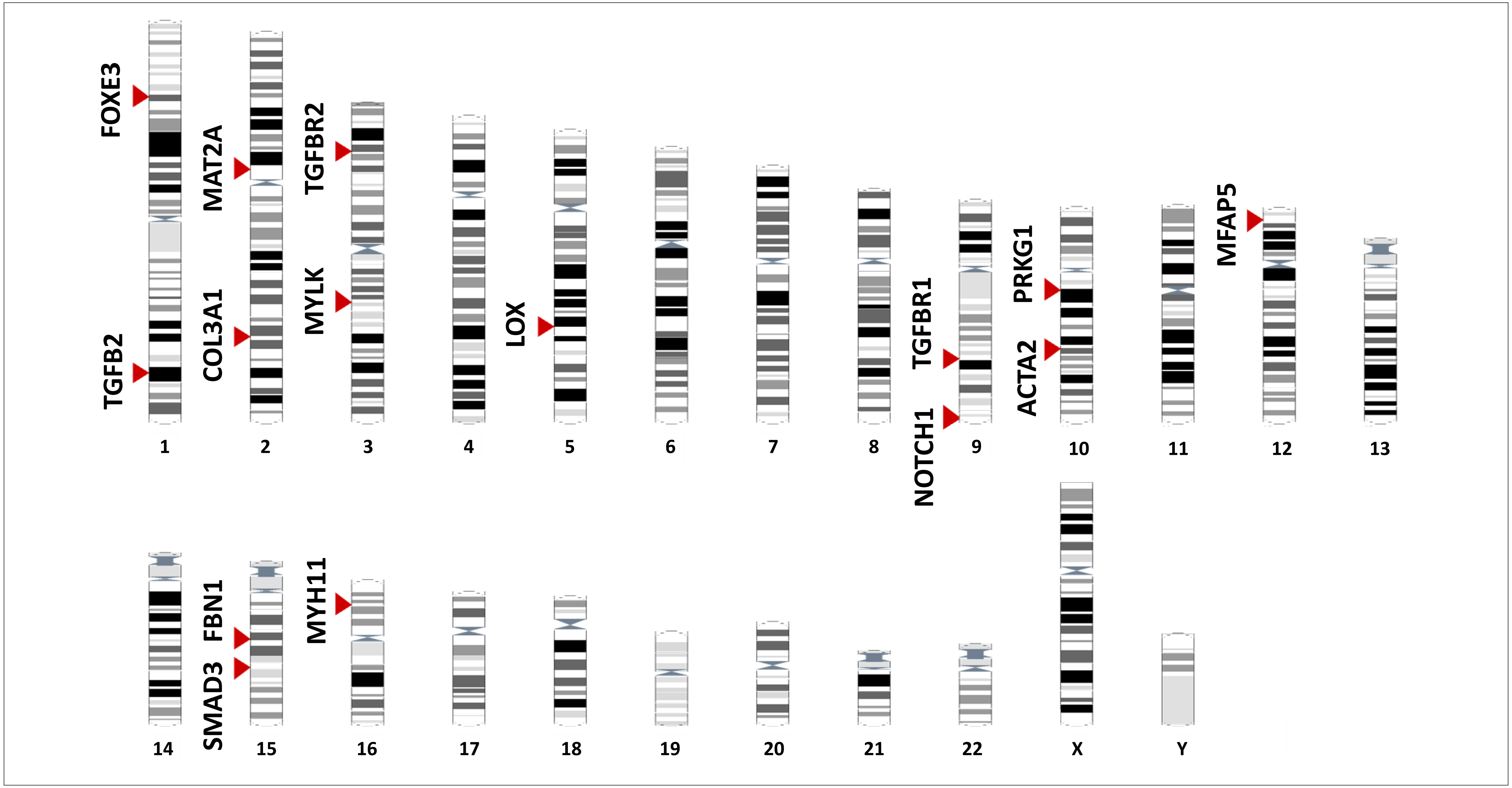

ACTA2 = actin alpha 2; COL3A1 = collagen type III alpha 1 chain; FBN1 = fibrillin 1; FOXE3 = Forkhead box E3; LOX = lysyl oxidase; MAT2A = methionine adenosyltransferase 2A; MFAP5 = microfibrillar associated protein 5; MYH11 = myosin heavy chain 11**;** MYLK = myosin light chain kinase; PRKG1 = protein kinase-cGMP-dependent type I; SMAD3 = SMAD family member 3; TGFB2 = Transforming growth factor beta 2; TGFBR1 = transforming growth factor beta receptor 1; TGFBR2 = transforming growth factor beta receptor 2**.**

Supplemental References:

1. Barbier M, Gross MS, Aubart M, Hanna N, Kessler K, Guo DC, Tosolini L, Ho-Tin-Noe B, Regalado E, Varret M, Abifadel M, Milleron O, Odent S, Dupuis-Girod S, Faivre L, Edouard T, Dulac Y, Busa T, Gouya L, Milewicz DM, Jondeau G, Boileau C. MFAP5 loss-of-function mutations underscore the involvement of matrix alteration in the pathogenesis of familial thoracic aortic aneurysms and dissections. *Am J Hum Genet.* 2014;95:736-43.
2. Bee KJ, Wilkes DC, Devereux RB, Basson CT, Hatcher CJ. TGFβRIIb mutations trigger aortic aneurysm pathogenesis by altering transforming growth factor β2 signal transduction. *Circ Cardiovasc Genet.* 2012;5:621-9.
3. Chamney S, McGimpsey S, McConnell V, Willoughby CE. Iris Flocculi as an ocular marker of ACTA2 mutation in familial thoracic aortic aneurysms and dissections. *Ophthalmic Genet.* 2015;36:86-8.
4. Disabella E, Grasso M, Gambarin FI, Narula N, Dore R, Favalli V, Serio A, Antoniazzi E, Mosconi M, Pasotti M, Odero A, Arbustini E. Risk of dissection in thoracic aneurysms associated with mutations of smooth muscle alpha-actin 2 (ACTA2). *Heart.* 2011;97:321-6.
5. Disertori M, Bertagnolli C, Thiene G, Ferro A, Bonmassari R, Girardini D, Casarotto D. Familial dissecting aortic aneurysm. *G Ital Cardiol.* 1991;21:849-53
6. Dong SB, Zheng J, Ma WG, Chen MJ, Cheng LJ, He L, Xing QH, Sun LZ. Identification and surgical repair of familial thoracic aortic aneurysm and dissection caused by TGFBR1 mutation. *Ann Vasc Surg.* 2014;28:1909-12.
7. Francke U, Berg MA, Tynan K, Brenn T, Liu W, Aoyama T, Gasner C, Miller DC, Furthmayr H. A Gly1127Ser mutation in an EGF-like domain of the fibrillin-1 gene is a risk factor for ascending aortic aneurysm and dissection. *Am J Hum Genet.* 1995;56:1287-96.
8. Gago-Díaz M, Blanco-Verea A, Teixidó-Turà G, Valenzuela I, Del Campo M, Borregan M, Sobrino B, Amigo J, García-Dorado D, Evangelista A, Carracedo A, Brion M. Whole exome sequencing for the identification of a new mutation in TGFB2 involved in a familial case of non-syndromic aortic disease. *Clin Chim Acta.* 2014;437:88-92.
9. Gago-Díaz M, Blanco-Verea A, Teixidó G, Huguet F, Gut M, Laurie S, Gut I, Carracedo Á, Evangelista A, Brion M. PRKG1 and genetic diagnosis of early-onset thoracic aortic disease. *Eur J Clin Invest.* 2016;46:787-94.
10. Guo D, Hasham S, Kuang SQ, Vaughan CJ, Boerwinkle E, Chen H, Abuelo D, Dietz HC, Basson CT, Shete SS, Milewicz DM. Familial thoracic aortic aneurysms and dissections: genetic heterogeneity with a major locus mapping to 5q13-14. *Circulation.* 2001;103:2461-8.
11. Guo DC, Pannu H, Tran-Fadulu V, Papke CL, Yu RK, Avidan N, Bourgeois S, Estrera AL, Safi HJ, Sparks E, Amor D, Ades L, McConnell V, Willoughby CE, Abuelo D, Willing M, Lewis RA, Kim DH, Scherer S, Tung PP, Ahn C, Buja LM, Raman CS, Shete SS, Milewicz DM. Mutations in smooth muscle alpha-actin (ACTA2) lead to thoracic aortic aneurysms and dissections. *Nat Genet.* 2007;39:1488-93.
12. Guo DC, Papke CL, Tran-Fadulu V, Regalado ES, Avidan N, Johnson RJ, Kim DH, Pannu H, Willing MC, Sparks E, Pyeritz RE, Singh MN, Dalman RL, Grotta JC, Marian AJ, Boerwinkle EA, Frazier LQ, LeMaire SA, Coselli JS, Estrera AL, Safi HJ, Veeraraghavan S, Muzny DM, Wheeler DA, Willerson JT, Yu RK, Shete SS, Scherer SE, Raman CS, Buja LM, Milewicz DM. Mutations in smooth muscle alpha-actin (ACTA2) cause coronary artery disease, stroke, and Moyamoya disease, along with thoracic aortic disease. *Am J Hum Genet.* 2009;84:617-27.
13. Guo DC, Regalado ES, Minn C, Tran-Fadulu V, Coney J, Cao J, Wang M, Yu RK, Estrera AL, Safi HJ, Shete SS, Milewicz DM. Familial thoracic aortic aneurysms and dissections: identification of a novel locus for stable aneurysms with a low risk for progression to aortic dissection. *Circ Cardiovasc Genet.* 2011;4:36-42.
14. Guo DC, Regalado E, Casteel DE, Santos-Cortez RL, Gong L, Kim JJ, Dyack S, Horne SG, Chang G, Jondeau G, Boileau C, Coselli JS, Li Z, Leal SM, Shendure J, Rieder MJ, Bamshad MJ, Nickerson DA; GenTAC Registry Consortium; National Heart, Lung, and Blood Institute Grand Opportunity Exome Sequencing Project, Kim C, Milewicz DM. Recurrent gain-of-function mutation in PRKG1 causes thoracic aortic aneurysms and acute aortic dissections. *Am J Hum Genet.* 2013;93:398-404.
15. Guo DC, Gong L, Regalado ES, Santos-Cortez RL, Zhao R, Cai B, Veeraraghavan S, Prakash SK, Johnson RJ, Muilenburg A, Willing M, Jondeau G, Boileau C, Pannu H, Moran R, Debacker J; GenTAC Investigators, National Heart, Lung, and Blood Institute Go Exome Sequencing Project; Montalcino Aortic Consortium, Bamshad MJ, Shendure J, Nickerson DA, Leal SM, Raman CS, Swindell EC, Milewicz DM. MAT2A mutations predispose individuals to thoracic aortic aneurysms. *Am J Hum Genet.* 2015;96:170-7.
16. Guo DC, Regalado ES, Gong L, Duan X, Santos-Cortez RL, Arnaud P, Ren Z, Cai B, Hostetler EM, Moran R, Liang D, Estrera A, Safi HJ; University of Washington Center for Mendelian Genomics, Leal SM, Bamshad MJ, Shendure J, Nickerson DA, Jondeau G, Boileau C, Milewicz DM. LOX mutations predispose to thoracic aortic aneurysms and dissections. *Circ Res.* 2016;118:928-34.

17. Hannuksela M, Stattin EL, Johansson B, Carlberg B. Screening for familial thoracic aortic aneurysms with aortic imaging does not detect all potential carriers of the disease. Aorta (Stamford). 2015;3(1):1-8.
18. Hannuksela M, Stattin EL, Klar J, Ameur A, Johansson B, Sörensen K, Carlberg B. A novel variant in MYLK causes thoracic aortic dissections: genotypic and phenotypic description. *BMC Med Genet.* 2016;17:61.
19. Harakalova M1, van der Smagt J, de Kovel CG, Van't Slot R, Poot M, Nijman IJ, Medic J, Joziasse I, Deckers J, Roos-Hesselink JW, Wessels MW, Baars HF, Weiss MM, Pals G, Golmard L, Jeunemaitre X, Lindhout D, Cuppen E, Baas AF. Incomplete segregation of MYH11 variants with thoracic aortic aneurysms and dissections and patent ductus arteriosus. *Eur J Hum Genet.* 2013;21:487-93.
20. Hasham SN, Willing MC, Guo DC, Muilenburg A, He R, Tran VT, Scherer SE, Shete SS, Milewicz DM. Mapping a locus for familial thoracic aortic aneurysms and dissections (TAAD2) to 3p24-25. *Circulation.* 2003;107:3184-90.
21. Kakko S, Räisänen T, Tamminen M, Airaksinen J, Groundstroem K, Juvonen T, Ylitalo A, Uusimaa P, Savolainen MJ. Candidate locus analysis of familial ascending aortic aneurysms and dissections confirms the linkage to the chromosome 5q13-14 in Finnish families. *J Thorac Cardiovasc Surg.* 2003;126:106-13.
22. Kent KC, Crenshaw ML, Goh DL, Dietz HC. Genotype-phenotype correlation in patients with bicuspid aortic valve and aneurysm. *J Thorac Cardiovasc Surg.* 2013;146:158-165.
23. Keramati AR, Sadeghpour A, Farahani MM, Chandok G, Mani A. The non-syndromic familial thoracic aortic aneurysms and dissections maps to 15q21 locus. *BMC Med Genet.* 2010;11:143.
24. Khau Van Kien P, Wolf JE, Mathieu F, Zhu L, Salve N, Lalande A, Bonnet C, Lesca G, Plauchu H, Dellinger A, Nivelon-Chevallier A, Brunotte F, Jeunemaitre X. Familial thoracic aortic aneurysm/dissection with patent ductus arteriosus: genetic arguments for a particular pathophysiological entity. *Eur J Hum Genet.* 2004;12:173-80.
25. Khau Van Kien P, Mathieu F, Zhu L, Lalande A, Betard C, Lathrop M, Brunotte F, Wolf JE, Jeunemaitre X. Mapping of familial thoracic aortic aneurysm/dissection with patent ductus arteriosus to 16p12.2-p13.13. *Circulation.* 2005;112:200-6.
26. Kuang SQ, Medina-Martinez O, Guo DC, Gong L, Regalado ES, Reynolds CL, Boileau C, Jondeau G, Prakash SK, Kwartler CS, Zhu LY, Peters AM, Duan XY, Bamshad MJ, Shendure J, Nickerson DA, Santos-Cortez RL, Dong X, Leal SM, Majesky MW, Swindell EC, Jamrich M, Milewicz DM. FOXE3 mutations predispose to thoracic aortic aneurysms and dissections. *J Clin Invest.* 2016;126:948-61.
27. Loscalzo ML, Goh DL, Loeys B, Kent KC, Spevak PJ, Dietz HC. Familial thoracic aortic dilation and bicommissural aortic valve: a prospective analysis of natural history and inheritance. *Am J Med Genet A.* 2007 ;143A:1960-7.
28. Marwick TH1, Woodhouse SP, Birchley IN, Strong RW. Management of familial aortic dissection. *Chest.* 1987;92:954-6.
29. McManus BM, Cassling RS, Soundy TJ, Wilson JE, Sears TD, Rogler WC, Buehler BA, Wolford JF, Duggan MJ, Byers PH, Fleming WH, Sanger WG. Familial aortic dissection in absence of ascending aortic aneurysms: a lethal syndrome associated with precocious systemic hypertension. *Am J Cardiovasc Pathol.* 1987;1:55-67.
30. Milewicz DM, Chen H, Park ES, Petty EM, Zaghi H, Shashidhar G, Willing M, Patel V. Reduced penetrance and variable expressivity of familial thoracic aortic aneurysms/dissections. *Am J Cardiol.* 1998;82:474-9.
31. Morisaki H, Akutsu K, Ogino H, Kondo N, Yamanaka I, Tsutsumi Y, Yoshimuta T, Okajima T, Matsuda H, Minatoya K, Sasaki H, Tanaka H, Ishibashi-Ueda H, Morisaki T. Mutation of ACTA2 gene as an important cause of familial and nonfamilial nonsyndromatic thoracic aortic aneurysm and/or dissection (TAAD). *Hum Mutat.* 2009;30:1406-11.
32. Pannu H, Fadulu VT, Chang J, Lafont A, Hasham SN, Sparks E, Giampietro PF, Zaleski C, Estrera AL, Safi HJ, Shete S, Willing MC, Raman CS, Milewicz DM. Mutations in transforming growth factor-beta receptor type II cause familial thoracic aortic aneurysms and dissections. *Circulation.* 2005;26;112:513-20.
33. Pannu H, Tran-Fadulu V, Papke CL, Scherer S, Liu Y, Presley C, Guo D, Estrera AL, Safi HJ, Brasier AR, Vick GW, Marian AJ, Raman CS, Buja LM, Milewicz DM. MYH11 mutations result in a distinct vascular pathology driven by insulin-like growth factor 1 and angiotensin II. *Hum Mol Genet.* 2007;16:2453-62.
34. Regalado E, Medrek S, Tran-Fadulu V, Guo DC, Pannu H, Golabbakhsh H, Smart S, Chen JH, Shete S, Kim DH, Stern R, Braverman AC, Milewicz DM. Autosomal dominant inheritance of a predisposition to thoracic aortic aneurysms and dissections and intracranial saccular aneurysms. *Am J Med Genet A.* 2011;155A:2125-30.
35. Regalado ES, Guo DC, Villamizar C, Avidan N, Gilchrist D, McGillivray B, Clarke L, Bernier F, Santos-Cortez RL, Leal SM, Bertoli-Avella AM, Shendure J, Rieder MJ, Nickerson DA; NHLBI GO Exome Sequencing Project, Milewicz DM. Exome sequencing identifies SMAD3 mutations as a cause of familial thoracic aortic aneurysm and dissection with intracranial and other arterial aneurysms. *Circ Res.* 2011;109:680-6.
36. Regalado ES, Guo DC, Santos-Cortez RL, Hostetler E, Bensend TA, Pannu H, Estrera A, Safi H, Mitchell AL, Evans JP, Leal SM, Bamshad M, Shendure J, Nickerson DA; University of Washington Center for Mendelian

Genomics, Milewicz DM. Pathogenic FBN1 variants in familial thoracic aortic aneurysms and dissections. *Clin Genet.* 2016;89:719-23.

37. Renard M, Callewaert B, Baetens M, Campens L, MacDermot K, Fryns JP, Bonduelle M, Dietz HC, Gaspar IM, Cavaco D, Stattin EL, Schrander-Stumpel C, Coucke P, Loeys B, De Paepe A, De Backer J. Novel MYH11 and ACTA2 mutations reveal a role for enhanced TGFβ signaling in FTAAD. *Int J Cardiol.* 2013;165:314-21.
38. Robertson EN, van der Linde D, Sherrah AG, Vallely MP, Wilson M, Bannon PG, Jeremy RW. Familial non-syndromal thoracic aortic aneurysms and dissections - Incidence and family screening outcomes. *Int J Cardiol.* 2016;220:43-51.
39. Sherrah AG, Andvik S, van der Linde D, Davies L, Bannon PG, Padang R, Vallely MP, Wilson MK, Keech AC, Jeremy RW. Nonsyndromic Thoracic aortic aneurysm and dissection: outcomes with Marfan syndrome versus bicuspid aortic valve aneurysm. *J Am Coll Cardiol.* 2016;67:618-26.
40. Takeda N, Morita H, Fujita D, Inuzuka R, Taniguchi Y, Nawata K, Komuro I. A deleterious MYH11 mutation causing familial thoracic aortic dissection. *Hum Genome Var.* 2015;2:15028.
41. Teixidó-Turà G, Valenzuela I, Gutiérrez L, Borregan M, del Campo M, Evangelista A. Nonsyndromic familial aortic disease: an underdiagnosed entity. *Rev Esp Cardiol (Engl Ed).* 2014;67:861-3.
42. Tran-Fadulu V, Chen JH, Lemuth D, Neichoy BT, Yuan J, Gomes N, Sparks E, Kramer LA, Guo D, Pannu H, Braverman AC, Shete S, Milewicz DM. Familial thoracic aortic aneurysms and dissections: three families with early-onset ascending and descending aortic dissections in women. *Am J Med Genet A.* 2006;140:1196-202.
43. Tortora G, Wischmeijer A, Berretta P, Alfonsi J, Marco L, Barbieri A, Marconi C, Isidori F, Rossi C, Leone O, Di Bartolomeo R, Seri M, Pacini D. Search for genetic factors in bicuspid aortic valve disease: ACTA 2 mutations do not play a major role. *Interact Cardiovasc Thorac Surg.* 2017;25:813-7.
44. Tran-Fadulu V, Pannu H, Kim DH, Vick GW 3rd, Lonsford CM, Lafont AL, Boccalandro C, Smart S, Peterson KL, Hain JZ, Willing MC, Coselli JS, LeMaire SA, Ahn C, Byers PH, Milewicz DM. Analysis of multigenerational families with thoracic aortic aneurysms and dissections due to TGFBR1 or TGFBR2 mutations. *J Med Genet.* 2009;46:607-13.
45. Vaughan CJ, Casey M, He J, Veugelers M, Henderson K, Guo D, Campagna R, Roman MJ, Milewicz DM, Devereux RB, Basson CT. Identification of a chromosome 11q23.2-q24 locus for familial aortic aneurysm disease, a genetically heterogeneous disorder. *Circulation.* 2001;103:2469-75.
46. Wang L, Guo DC, Cao J, Gong L, Kamm KE, Regalado E, Li L, Shete S, He WQ, Zhu MS, Offermanns S, Gilchrist D, Elefteriades J, Stull JT, Milewicz DM. Mutations in myosin light chain kinase cause familial aortic dissections. *Am J Hum Genet.* 2010;87:701-7.
47. Wang WJ, Han P, Zheng J, Hu FY, Zhu Y, Xie JS, Guo J, Zhang Z, Dong J, Zheng GY, Cao H, Liu TS, Fu Q, Sun L, Yang BB, Tian XL. Exon 47 skipping of fibrillin-1 leads preferentially to cardiovascular defects in patients with thoracic aortic aneurysms and dissections. *J Mol Med (Berl).* 2013;91:37-47.
48. Ware SM, Shikany A, Landis BJ, James JF, Hinton RB. Twins with progressive thoracic aortic aneurysm, recurrent dissection and ACTA2 mutation. *Pediatrics.* 2014;134:e1218-23.
49. Warnes CA, Kirkman PM, Roberts WC. Aortic dissection in more than one family member. *Am J Cardiol.* 1985;55:236-8.
50. Weigang E, Chang XC, Munk-Schulenburg S, Richter H, von Samson P, Goebel H, Frydrychowicz A, Geibel A, Ammann S, Schwering L, Brunner T, Severin T, Czerny M, Beyersdorf F. Actual management of patients with familial ascending aortic aneurysms and type-A aortic dissections. *Thorac Cardiovasc Surg*. 2007;55:19-23.
51. Yoo EH, Choi SH, Jang SY, Suh YL, Lee I, Song JK, Choe YH, Kim JW, Ki CS, Kim DK. Clinical, pathological, and genetic analysis of a Korean family with thoracic aortic aneurysms and dissections carrying a novel Asp26Tyr mutation. *Ann Clin Lab Sci.* 2010;40:278-84.
52. Zhu L, Vranckx R, Khau Van Kien P, Lalande A, Boisset N, Mathieu F, Wegman M, Glancy L, Gasc JM, Brunotte F, Bruneval P, Wolf JE, Michel JB, Jeunemaitre X.Mutations in myosin heavy chain 11 cause a syndrome associating thoracic aortic aneurysm/aortic dissection and patent ductus arteriosus. *Nat Genet.* 2006;38:343-9.
53. Ziganshin BA, Bailey AE, Coons C, Dykas D, Charilaou P, Tanriverdi LH, Liu L, Tranquilli M, Bale AE, Elefteriades JA. Routine genetic testing for thoracic aortic aneurysm and dissection in a clinical setting. *Ann Thorac Surg.* 2015;100:1604-11.
54. Roman MJ, Devereux RB, Kramer-Fox R, O'Loughlin J. Two-dimensional echocardiographic aortic root dimensions in normal children and adults. *Am J Cardiol.* 1989;64:507-12.
55. Mirea O, Maffessanti F, Gripari P, et al. Effects of aging and body size on proximal and ascending aorta and aortic arch: inner edge-to-inner edge reference values in a large adult population by two-dimensional transthoracic echocardiography. J Am Soc Echocardiogr 2013;26:419-27.
56. Davis AE, Lewandowski AJ, Holloway CJ, Ntusi NA, Banerjee R, Nethononda R, Pitcher A, Francis JM, Myerson SG, Leeson P, Donovan T, Neubauer S, Rider OJ. Observational study of regional aortic size

referenced to body size: production of a cardiovascular magnetic resonance nomogram. *J Cardiovasc Magn Reson.* 2014;16:9.

57. Vasan RS, Larson MG, Benjamin EJ, Levy D. Echocardiographic reference values for aortic root size: the Framingham Heart Study. *J Am Soc Echocardiogr.* 1995;8:793-800. doi: 10.1016/S0894-7317(05)80003-3.
58. Wolak A, Gransar H, Thomson LE, Friedman JD, Hachamovitch R, Gutstein A, Shaw LJ, Polk D, Wong ND, Saouaf R, Hayes SW, Rozanski A, Slomka PJ, Germano G, Berman DS. Aortic size assessment by noncontrast cardiac computed tomography: normal limits by age, gender, and body surface area. *JACC Cardiovasc Imaging.* 2008;1:200-9.
59. Wells GA, Shea B, O'Connell D, Peterson J, Welch V, Losos M, Tugwell P. The Newcastle-Ottawa Scale (NOS) for assessing the quality of nonrandomised studies in meta-analyses. Ottawa Hospital Research Institute. Available at: http://www.ohri.ca/programs/clinical_epidemiology/oxford.asp. Accessed June 30, 2017.
60. Higgins JPT, Green S (editors). Cochrane Handbook for systematic reviews of interventions. Chichester, UK: John Wiley and Sons; 2008.
61. Harris RP, Helfand M, Woolf SH, Lohr KN, Mulrow CD, Teutsch SM, Atkins D; Methods Work Group, Third US Preventive Services Task Force. Current methods of the US Preventive Services Task Force: a review of the process. *Am J Prev Med.* 2001;20(3 Suppl):21-35.
62. Hiratzka LF, Bakris GL, Beckman JA, Bersin RM, Carr VF, Casey DE Jr, Eagle KA, Hermann LK, Isselbacher EM, Kazerooni EA, Kouchoukos NT, Lytle BW, Milewicz DM, Reich DL, Sen S, Shinn JA, Svensson LG, Williams DM; American College of Cardiology Foundation; American Heart Association Task Force on Practice Guidelines; American Association for Thoracic Surgery; American College of Radiology; American Stroke Association; Society of Cardiovascular Anesthesiologists; Society for Cardiovascular Angiography and Interventions; Society of Interventional Radiology; Society of Thoracic Surgeons; Society for Vascular Medicine. 2010 ACCF/AHA/AATS/ACR/ASA/SCA/SCAI/SIR/STS/SVM Guidelines for the diagnosis and management of patients with thoracic aortic disease. A Report of the American College of Cardiology Foundation/American Heart Association Task Force on Practice Guidelines, American Association for Thoracic Surgery, American College of Radiology, American Stroke Association, Society of Cardiovascular Anesthesiologists, Society for Cardiovascular Angiography and Interventions, Society of Interventional Radiology, Society of Thoracic Surgeons, and Society for Vascular Medicine. *J Am Coll Cardiol*. 2010;55:e27-e129.
63. Erbel R, Aboyans V, Boileau C, Bossone E, Bartolomeo RD, Eggebrecht H, Evangelista A, Falk V, Frank H, Gaemperli O, Grabenwöger M, Haverich A, Iung B, Manolis AJ, Meijboom F, Nienaber CA, Roffi M, Rousseau H, Sechtem U, Sirnes PA, Allmen RS, Vrints CJ; ESC Committee for Practice Guidelines. 2014 ESC Guidelines on the diagnosis and treatment of aortic diseases: Document covering acute and chronic aortic diseases of the thoracic and abdominal aorta of the adult. The Task Force for the Diagnosis and Treatment of Aortic Diseases of the European Society of Cardiology (ESC). *Eur Heart J*. 2014;35:2873-926.
64. Verstraeten A, Luyckx I, Loeys B. Aetiology and management of hereditary aortopathy. *Nat Rev Cardiol.* 2017;14:197-208.
65. Brownstein AJ, Ziganshin BA, Kuivaniemi H, Body SC, Bale AE, Elefteriades JA. Genes Associated with Thoracic Aortic Aneurysm and Dissection. An update and clinical implications. *Aorta (Stamford).* 2017;5:11-20.
